# Hippocampal clock regulates memory retrieval via Dopamine and PKA-induced GluA1 phosphorylation

**DOI:** 10.1038/s41467-019-13554-y

**Published:** 2019-12-18

**Authors:** Shunsuke Hasegawa, Hotaka Fukushima, Hiroshi Hosoda, Tatsurou Serita, Rie Ishikawa, Tomohiro Rokukawa, Ryouka Kawahara-Miki, Yue Zhang, Miho Ohta, Shintaro Okada, Toshiyuki Tanimizu, Sheena A. Josselyn, Paul W. Frankland, Satoshi Kida

**Affiliations:** 1 Department of Bioscience, Faculty of Applied Bioscience, Tokyo University of Agriculture, Tokyo, 156-8502 Japan; 2 CREST, Japan Science and Technology Agency, Saitama, 332-0012 Japan; 3 Graduate School of Agriculture and Life Sciences, The University of Tokyo, Tokyo, 113-8657 Japan; 4 NODAI Genome Research Center, Tokyo University of Agriculture, Tokyo, 156-8502 Japan; 5 Department of Physiology, Faculty of Medicine, University of Toronto, Toronto, ON M5S 1A8 Canada; 6 Program in Neurosciences and Mental Health, Hospital for Sick Children, Toronto, ON Canada

**Keywords:** Hippocampus, Long-term memory

## Abstract

Cognitive performance in people varies according to time-of-day, with memory retrieval declining in the late afternoon-early evening. However, functional roles of local brain circadian clocks in memory performance remains unclear. Here, we show that hippocampal clock controlled by the circadian-dependent transcription factor BMAL1 regulates time-of-day retrieval profile. Inducible transgenic dominant negative BMAL1 (dnBMAL1) expression in mouse forebrain or hippocampus disrupted retrieval of hippocampal memories at Zeitgeber Time 8–12, independently of retention delay, encoding time and Zeitgeber entrainment cue. This altered retrieval profile was associated with downregulation of hippocampal Dopamine-cAMP signaling in dnBMAL1 mice. These changes included decreases in Dopamine Receptors (D1-R and D5-R) and GluA1-S845 phosphorylation by PKA. Consistently, pharmacological activation of cAMP-signals or D1/5Rs rescued impaired retrieval in dnBMAL1 mice. Importantly, GluA1 S845A knock-in mice showed similar retrieval deficits with dnBMAL1 mice. Our findings suggest mechanisms underlying regulation of retrieval by hippocampal clock through D1/5R-cAMP-PKA-mediated GluA1 phosphorylation.

## Abstract (web summary)

The neural mechanisms that lead to a relative deficit in memory retrieval in the afternoon are unclear. Here, the authors show that the circadian - dependent transcription factor BMAL1 regulates retrieval through dopamine and glutamate receptor phosphorylation.

## Introduction

Circadian rhythms regulate many physiological, biological, and behavioral processes in mammals. The primary mammalian circadian clock is thought to be the suprachiasmatic nuclei (SCN) of anterior hypothalamus. The molecular mechanisms mediating circadian rhythm generation in the SCN are well studied: BMAL1 (brain and muscle ARNT-like protein 1, also called MOP3) and CLOCK form a heterodimer and activate the transcription of their target genes, including the *Period* (*Per1*, *Per2*) and the *Cryptochrome* (*Cry1*, *Cry2*)^1–6^. The resulting gene products (PERs and CRYs) inhibit BMAL1/CLOCK-mediated transcription in a negative feedback loop, thereby generating circadian transcriptional rhythms^1,2^.

Interestingly, in addition to the SCN, BMAL1/CLOCK-mediated transcriptional rhythms are observed in other parts of the brain, as well as peripheral tissues, including muscle and liver^5–7^. The SCN generates circadian behavioral rhythms and synchronizes the local clocks in peripheral tissues^1,2^. Importantly, there is increasing evidence that molecular clocks in these extra-SCN regions exert autonomous circadian regulation of tissue-specific functions^1,8–14^. Although it has been known since experiments by Ebbinghaus (1885) that time-of-day influences cognitive performance and memory (wherein performance declines in the late morning to early evening^15^), the mechanisms underlying this effect are not well understood^16–19^. Here we investigated the local role of this molecular clock in the hippocampus in memory processes using mice.

## Results

### Time-of-day changes in memory retrieval performance in wild-type (WT) mice

**Fig. 1:**
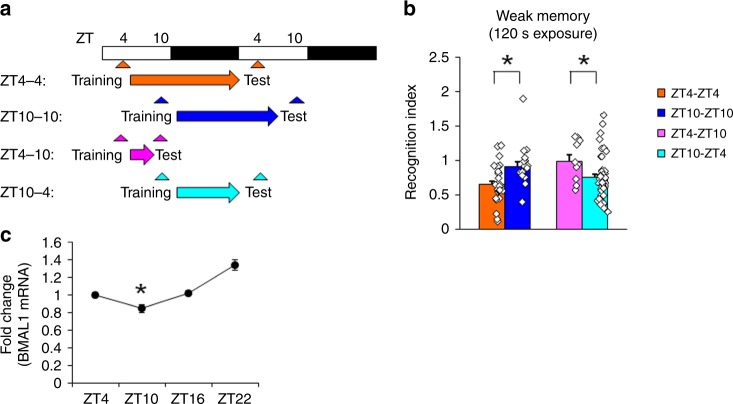
Retrieval of social recognition memory is impaired at ZT10 following weak training in wild-type (WT) mice. **a** Experimental design. **b** Memory retrieval (recognition index) is impaired (higher recognition index) following weak social recognition training (2 min exposure to novel mouse during training) at ZT10, independent of the time of training. **c** BMAL1 mRNA levels in the whole hippocampus are reduced at ZT10 compared to other time points (quantitative RT-PCR). The graph represents fold changes compared to expression levels at ZT4. *p < 0.05, compared to the other time points. All values are mean ± SEM. Individual data points are displayed as dots. *p < 0.05 as determined by two-way (**b**) or one-way (**c**) ANOVA with post hoc test. The results of the statistical analyses are presented in Supplementary Table 2. Source data are provided as a source data file.

We first asked whether mice also show similar time-of-day changes in the memory retrieval performance. We trained WT mice in a hippocampus-dependent social recognition task^20^ (Supplementary Fig. 1A, B). In this task, adult mice are exposed to a novel juvenile mouse and memory assessed in a test session in which the adult mouse is re-exposed to the same juvenile. In the first experiment, adult mice were trained (exposed to a juvenile mouse for 2 min) 4 h after lights on (Zeitgeber Time 4 (ZT4)) and memory assessed 24 h later (ZT4). In mice trained and tested at ZT4, we observed a reduction in exploration of the juvenile mice during this test compared to training, indicating recognition of the juvenile mouse (Fig. 1a, b, Supplementary Fig. 1E). In contrast, recognition was abolished in mice trained and tested at ZT10 (Fig. 1b, Supplementary Fig. 1E). This impairment could reflect a disruption of memory encoding or retrieval (or both). Therefore, we trained an additional group at ZT10 and tested mice at ZT4. These mice showed intact recognition, thereby excluding the possibility that memory encoding is impaired when mice are trained at ZT10. In contrast, mice trained at ZT4 but tested at ZT10 showed impaired recognition. This dissociation indicates that weakly trained WT mice have a memory retrieval deficit at ZT10 (Fig. 1b, Supplementary Fig. 1E). Importantly, strong training (exposure to juvenile mouse for 3 min) alleviates this time-of-day effect on memory retrieval (Supplementary Fig. 1C, D).

Time-of-day oscillations in the transcriptional activity of clock genes (including BMAL1 and CLOCK) in the central pacemaker, the SCN, are essential for generating circadian transcriptional rhythm^1–5^. BMAL1/CLOCK-mediated transcriptional rhythms are also observed outside of the SCN^5–7^ and there is increasing evidence that molecular clocks in extra-SCN regions exert autonomous circadian regulation of tissue-specific functions^1,8,9,14,21^. Therefore, we next examined whether BMAL1 levels in the hippocampal region cycle with memory retrieval performance. We observed that BMAL1 mRNA levels in WT mice were lowest at ZT10 (Fig. 1c), corresponding to the time of day when memory retrieval deficits were also observed.

### Forebrain-specific inhibition of BMAL1 function in transgenic mice

**Fig. 2:**
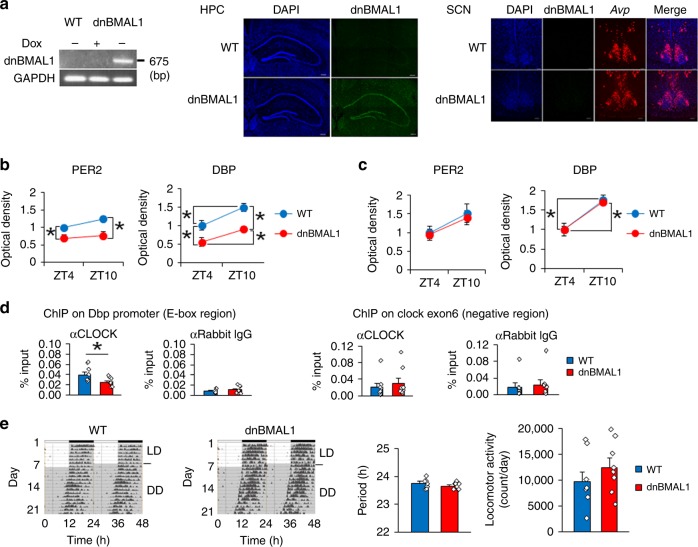
Forebrain-specific inhibition of BMAL1 function. **a** dnBMAL1 mRNA expression in dnBMAL1 mice. Dox-dependent dnBMAL1 mRNA expression in hippocampus (RT-PCR, left). dnBMAL1 mRNA expression in hippocampus (HPC, middle) but not SCN (right) (in situ hybridization). AVP mRNA expression as a marker of SCN. DAPI (nuclear stain, blue), dnBMAL1 (green), AVP (red). Scale bar, 200 μm (HPC) and 100 μm (SCN). **b**, **c** PER2 and expression levels (BMAL1 target genes) are reduced in hippocampal CA1 (**b**) but not in SCN (**c**), in dnBMAL1 mice at both ZT4 and 10. The graph represents fold changes compared to the expression levels in WT at ZT4. **d** dnBMAL1 blocks the CLOCK binding to Dbp promoter in the hippocampus of dnBMAL1 mice at ZT10. Anti-CLOCK antibody, but not anti-IgG, precipitated DBP promoter although DNA regions not containing E-box (*clock* gene exon 6) are comparably precipitated by anti-CLOCK antibody and anti-IgG. **e** Normal circadian locomotor rhythm in dnBMAL1 mice. Mice were housed in a 12 h light:12 h dark (LD) cycle then in constant darkness (DD). (Left) Representative activity records are double-plotted with each horizontal line representing 48 h. Circadian period (Middle) and daily locomotor activity (Right) under DD. All values are mean ± SEM. Individual data points are displayed as dots. *p < 0.05 as determined by two-way (**b**, **c**) or one-way (**d**, **e**) ANOVA with post hoc test. The results of the statistical analyses are presented in Supplementary Table 2. Source data are provided as a source data file.

Our results raise the possibility that levels of BMAL1 modulate the efficiency of retrieval of hippocampal-dependent memories. To directly test this idea, we generated mice in which BMAL1/CLOCK-mediated transcription was reduced exclusively in the forebrain (and not in the SCN). Mutant BMAL1 (R to A at residue 91) heterodimerizes with CLOCK but is unable to bind to E-box elements in the promoter sequences of target genes, thereby creating a dominant-negative BMAL1 (dnBMAL1)^22^. We made two lines of mice that inducibly express dnBMAL1 using a tetracycline-responsive element (TRE)-dependent promoter (TRE-Line and TRE-line B mice)^23,24^. These mice were crossed with transgenic mice that express a tetracycline-dependent transcriptional activator (tTA) under the forebrain-specific αCaMKII promoter^24^. Double transgenic mice (dnBMAL1 Line and Line-B mice; dnBMAL1 and dnBMAL1-B) expressed dnBMAL1 mRNA in the forebrain (including the hippocampus) in the absence of tetracycline [or the derivative, doxycycline (Dox)] and dnBMAL1 mRNA expression was suppressed by Dox administration (Fig. 2a, Supplementary Fig. 2A-D). To minimize potential effects of dnBMAL1 expression during development (see below), transgenic mice (dnBMAL1 mice) were treated with Dox until 8 weeks of age (transgene OFF) at which time Dox was removed (transgene ON) to induce dnBMAL1 expression (Fig. 2a, Supplementary Fig. 2D). Following removal of Dox, the expression of dnBMAL1 blocked binding of CLOCK to the E-box sequence in the hippocampus, suggesting that dnBMAL1 functions on BMAL1/CLOCK-mediated transcription as a dominant-negative mutant in vivo (Fig. 2d, Supplementary Fig. 2K). Consistent with this, both lines of transgenic mice showed lower expression of BMAL1/CLOCK-target genes (PER2 and DBP)^25,26^ in the hippocampus compared to WT littermates (Fig. 2b, Supplementary Fig. 2E, G, I, J). These findings suggest that dnBMAL1 reduces endogenous BMAL1 function but preserves its circadian modulation. Importantly, transgenic mice showed normal expression of these BMAL1/CLOCK-target genes in the SCN (Fig. 2c, Supplementary Fig. 2F, H) and dnBMAL1 mice showed normal circadian rhythm of locomotor activity under constant dark (DD) conditions, a behavior controlled by the SCN circadian clock (Fig. 2e, Supplementary Fig. 2L).

### dnBMAL1 mice show impaired memory retrieval

**Fig. 3:**
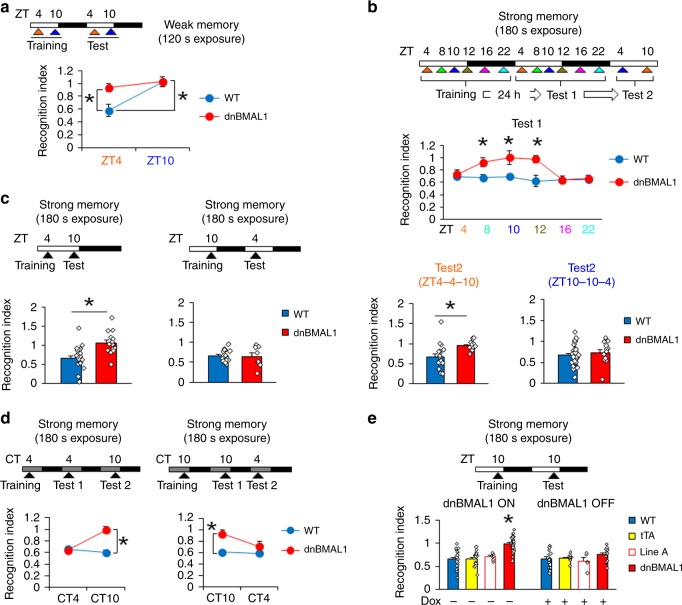
Impaired retrieval of social recognition memory in dnBMAL1 mice from ZT8 to ZT12 following strong training. Experimental designs are illustrated at the top of each panel. **a** Impaired memory both at ZT4 and ZT10 in dnBMAL1 mice but only at ZT10 in WT mice, following weak training at ZT4 or ZT10, respectively. **b** Separate groups are trained at ZT4, ZT8, ZT10, ZT12, ZT16, or ZT22 and tested 24 h later. (Upper) Retrieval at Test 1. Impaired retrieval in dnBMAL1 mice at ZT8–12. (Lower) Mice that are initially tested at ZT4 or are re-tested at ZT10 or ZT4, respectively. Impaired retrieval in dnBMAL1 mice re-tested at ZT10. *p < 0.05, compared to WT mice at each time point. **c** Retrieval impairment at ZT10 but not at ZT4 in dnBMAL1 mice trained at ZT4 or ZT10 and then tested at ZT10 or ZT4, respectively. **d** Under DD, impaired retrieval in dnBMAL1 mice at CT10. **e** Retrieval impairment depends on dnBMAL1 expression (not observed in dnBMAL1 OFF and single transgenic mice). dnBMAL1 ON mice display significantly worse recognition indexes compared to the control groups. *p < 0.05, compared to the other control groups. All values are mean ± SEM. Individual data points are displayed as dots. *p < 0.05 as determined by two-way (**a**, **b**, **d**, **e**) or one-way (**c**) ANOVA with post hoc test. The results of the statistical analyses are presented in Supplementary Table 2. Source data are provided as a source data file.

We previously showed that WT mice trained under weak training conditions showed a memory retrieval deficit in the social recognition task at ZT10, a time when BMAL1 levels are endogenously reduced. Next, we tested whether artificially decreasing BMAL1 function similarly disrupts memory retrieval at all times of the day. dnBMAL1 and WT mice were trained using weak training (2 min interaction with juvenile mice) at ZT4 or ZT10 and tested at ZT4 or ZT10, respectively (i.e., equivalent training conditions to those in Fig. 1b, Supplementary Fig. 1E). Consistent with previous observations, WT mice showed an impaired retrieval of social recognition memory at ZT10 (when endogenous BMAL1 levels are low) but not at ZT4. In contrast, dnBMAL1 mice showed retrieval deficits at both ZT4 and ZT10 (Fig. 3a, Supplementary Fig. 3A). Together, these results suggest that activities of BMAL1 regulate retrieval.

Because in dnBMAL1 mice, endogenous BMAL1 function is reduced but still shows circadian modulation (Fig. 2b), we next examined whether the memory retrieval deficits observed in dnBMAL1 mice could be rescued by strong training if mice were tested at a time of high endogenous BMAL1 (ZT4). We trained dnBMAL1 mice in the social recognition task using a strong training protocol (in which WT mice do not exhibit time-of-day retrieval deficits, Supplementary Fig. 1C, D). dnBMAL1 and WT littermate mice were trained at ZT4, ZT8, ZT10, ZT12, ZT16, or ZT22 and tested 24 h later (Fig. 3b, Supplementary Fig. 3C). Strikingly, dnBMAL1 mice showed normal memory retrieval when tested at ZT4, ZT16, and ZT22 (Fig. 3b, Supplementary Fig. 3C). To test time-of-day dependence of intact memory retrieval in dnBMAL1 mice, we re-tested mice at different ZT times. dnBMAL1 mice that showed intact memory at ZT4 subsequently showed a deficit when re-tested at ZT10. In contrast, dnBMAL1 mice that showed impaired memory when first tested at ZT10 showed normal memory when re-tested at ZT4 (Fig. 3b, Supplementary Fig. 3C). The spared memory we observed in dnBMAL1 mice re-tested at ZT4 in particular suggests that these effects are due to deficient memory retrieval, rather than deficient memory encoding. Using this strong training protocol, we next trained additional groups of mice at ZT4 and tested memory at ZT10 (Fig. 3c, Supplementary Fig. 3D). dnBMAL1, but not WT, mice showed impaired recognition memory at ZT10. In contrast, both dnBMAL1 and WT mice trained at ZT10 but tested at ZT4 showed normal recognition memory, confirming that the deficit observed in dnBMAL1 mice is specific to time-of-retrieval and independent of time-of-encoding (Fig. 3c, Supplementary Fig. 3D).

The above experiments were conducted in light–dark conditions (with lights on at ZT0). We also observed these time-of-day deficits in memory retrieval in mice tested in constant dark (DD) conditions (Fig. 3d, Supplementary Fig. 3E), indicating that the retrieval deficit does not depend on external time-of-day cues but is generated endogenously. We additionally note that we observed equivalent time-of-day retrieval deficits in the second dnBMAL1 line (dnBMAL1-B; Supplementary Fig. 3B). Furthermore, these retrieval deficits were only observed in dnBMAL1 mice in the absence of Dox (dnBMAL1 ON mice with transgene ON, Fig. 3e, Supplementary Figs. 2B, 3F-I). Finally, dnBMAL1 mice displayed normal anxiety-related behavior and normal forebrain anatomy (Supplementary Fig. 3J, K).

### The memory retrieval impairments in dnBMAL1 mice generalize to hippocampus-dependent memories

**Fig. 4:**
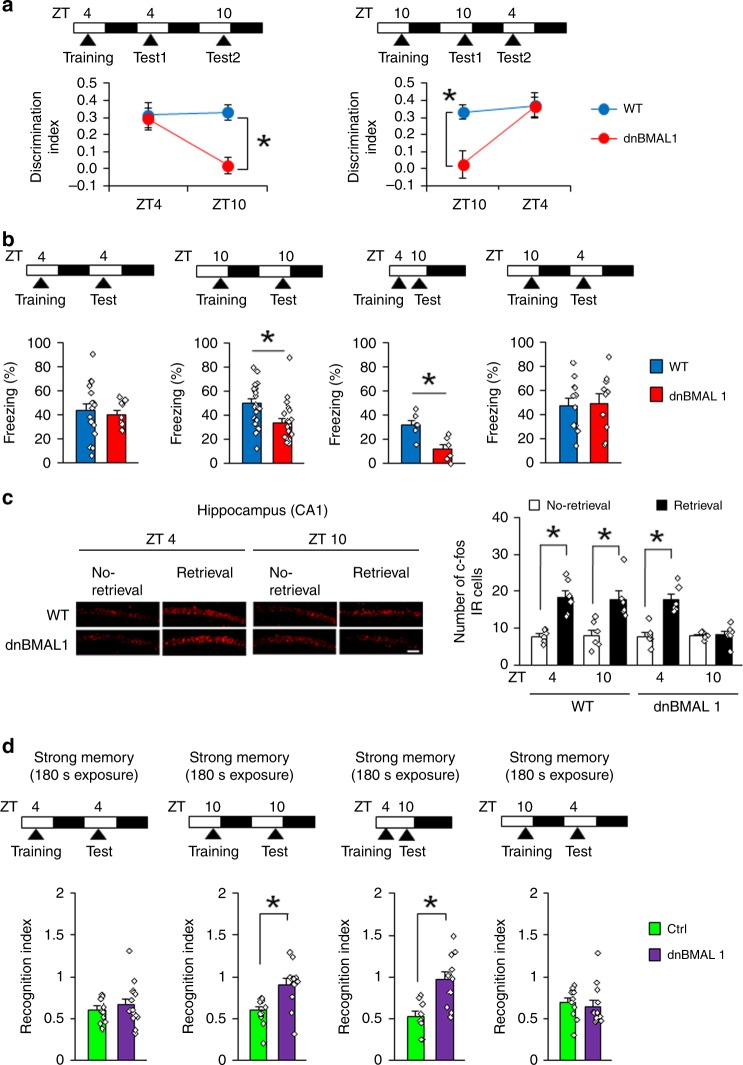
Memory retrieval deficits in dnBMAL1 mice generalize to hippocampus-dependent memory. **a**, **b** Experimental designs are illustrated at the top of each panel. Memory retrieval is impaired at ZT10 in dnBMAL1 mice for **a** novel object recognition task and **b** contextual fear memory. **c** Consistent with impaired memory retrieval at ZT10, dnBMAL1 mice show reduced c-fos expression in CA1 region of hippocampus after retrieval of contextual fear memory. (Left panels) Representative images of c-fos immunohistochemistry. Scale bar, 100 μm. (Right panel) Quantification of the number of c-fos immunoreactive (IR) cells. **d** dnBMAL1 expression in the hippocampus impairs retrieval of social recognition memory at ZT10 but not at ZT4 (Recognition index). Experimental designs are illustrated at the top of each panel. One-way ANOVAs with group reveal significant effect of dnBMAL1 when dnBMAL1 group is tested at ZT10 but not at ZT4. Ctrl control. All values are mean ± SEM. Individual data points are displayed as dots. *p < 0.05 as determined by three-way (**c**), two-way (**a**), or one-way (**b**, **d**) ANOVA with post hoc test. The results of the statistical analyses are presented in Supplementary Table 2. Source data are provided as a source data file.

To examine whether these time-of-day retrieval deficits in dnBMAL1 mice generalize to other forms of memory, we next used an object recognition task. During training at ZT4, mice were presented with two objects. When tested 24 h later (at ZT4), mice were presented with one of the original objects and a novel object. Both WT littermate and dnBMAL1 mice spent more time exploring the novel object (indicating recognition memory). However, when subsequently re-tested at ZT10, whereas WT mice spent more time exploring a new object, dnBMAL1 mice spent similar times exploring both the new and the original object (Fig. 4a). These time-of-day-specific retrieval deficits in dnBMAL1 mice were confirmed in another experiment in which mice were trained at ZT10. dnBMAL1 mice showed impaired retrieval when tested 24 h later (ZT10) but not when re-tested at 18 h later at ZT4 (Fig. 4a).

A similar pattern of results was observed using contextual fear conditioning^27^ (Fig. 4b, Supplementary Fig. 4A–C). Only dnBMAL1 mice trained and tested at ZT10 (and not ZT4) showed impaired recognition of a context previously paired with shock, and as before, these deficits were due to time-of-retrieval, rather than time-of-memory encoding. Consistent with this, retrieval-induced increases in the levels of activity-regulated gene c-fos in the CA1 region of the hippocampus^28^ were absent in dnBMAL1 mice tested at ZT10 but not at ZT4 (Fig. 4c).

### Expression of dnBMAL1 only in hippocampus disrupts memory retrieval

The retrieval impairments of hippocampus-dependent memories by forebrain expression of dnBMAL1 suggests that a hippocampal circadian clock regulates retrieval. To test this possibility, we examined effects of hippocampus-specific expression of dnBMAL1 using adeno-associated virus (AAV). We observed the expression of enhanced green fluorescent protein (EGFP) co-expressed with dnBMAL1 in the dorsal hippocampus and found that dnBMAL1 expression reduced the expression level of PER2 in the CA1 region of hippocampus compared to the control group (Supplementary Fig. 4D), suggesting that similar with dnBMAL1 mice (Fig. 2b), viral expression of dnBMAL1 impairs hippocampal circadian clock. Importantly, mice expressing dnBMAL1, but not the control group, showed impaired social recognition memory at ZT10 but not at ZT4 (Fig. 4d, Supplementary Fig. 4E). In contrast, dnBMAL1 group trained at ZT10 and tested at ZT4 showed normal recognition memory (Fig. 4d, Supplementary Fig. 4E). It is important to note that the dnBMAL1 group showed normal locomotor activity and anxiety-related behavior (Supplementary Fig. 4F). Importantly, we observed similar patterns of changes in retrieval performance by knockdown of BMAL1 expression in the hippocampus (Supplementary Fig. 4H). These observations indicate that hippocampal expression of dnBMAL is sufficient to impair memory retrieval at ZT10, suggesting that retrieval deficits observed in dnBMAL1 mice are due to impaired hippocampal clock.

### Impaired hippocampal Dopamine (DA)-cAMP signals in dnBMAL1 mice

**Fig. 5:**
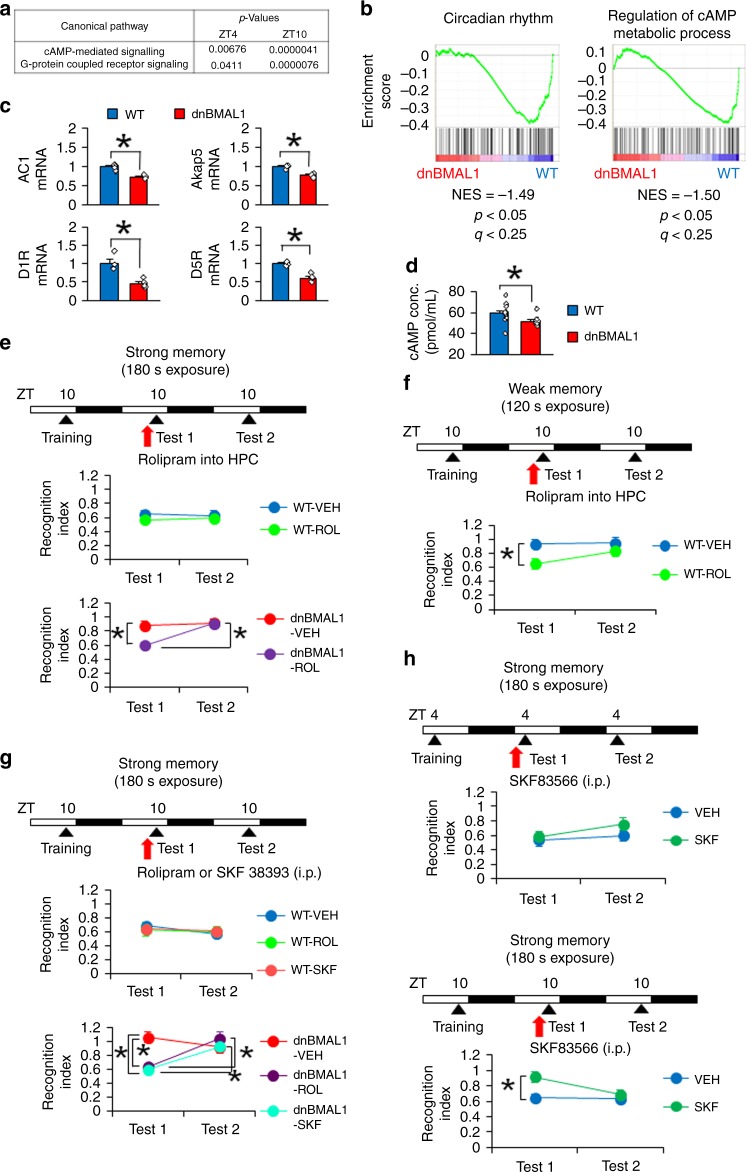
Impaired Dopamine D1/5R-cAMP signal and its pharmacological rescues of memory retrieval deficits. **a** Changes in cAMP-mediated signaling and G-protein coupled receptor signaling in the hippocampus of dnBMAL1 mice (ingenuity pathway analysis). **b** Downregulation of gene set for circadian rhythm (left) and regulation of cAMP metabolic process (right) in the hippocampus of dnBMAL1 mice at ZT10 (Enrichment plot). NES normalized enrichment score. **a**, **b** All groups (*n* = 3). **c**, **d** Reduced AC1, AKAP5, D1R, and D5R mRNAs (**c**) and cAMP (**d**) levels in the hippocampus of dnBMAL1 mice at ZT10. The graph represents fold changes compared to the expression levels in WT (**c**). **e**–**h** Experimental designs at the top of each panel. Retrieval is tested twice (Tests 1 and 2) at ZT10 following strong (**e**, **g**, **h**) or weak (**f**) training at ZT10. Dorsal hippocampal micro-infusion (**e**, **f**) or systemic injection (**g**, **h**) of drug at 30 min before Test 1. Rescues of impaired retrieval by hippocampal rolipram in dnBMAL1 (**e**, strong training) and WT mice (**f**, weak training) and by systemic SKF38393 (D1/5R agonist) or rolipram injection at Test 1 in dnBMAL1 mice (**g**). Impaired retrieval by systemic SKF83566 injection (D1/5R antagonist) in WT mice (**h**, Supplementary Fig. 5). HPC hippocampus, VEH vehicle. All values are mean ± SEM. Individual data points are displayed as dots. *p < 0.05 as determined by three-way (**e**, **g**), two-way (**f**, **h**), or one-way (**c**, **d**) ANOVA with post hoc test. The results of the statistical analyses are presented in Supplementary Table 2. Source data are provided as a source data file.

To understand molecular mechanisms for impaired memory retrieval in the dnBMAL1 mice, we performed RNA-sequencing (RNA-seq) of hippocampus. Ingenuity pathway analysis (IPA) indicated that dnBMAL1 mice exhibit significant downregulation of cAMP-mediated signaling and changes in G-protein coupled receptor signaling. Importantly, these dnBMAL1 effects on cAMP-mediated signaling were more pronounced at ZT10 compared to at ZT4 (Fig. 5a). Furthermore, Gene Set Enrichment Analysis (GSEA) revealed significant decreases in gene expression levels of regulation of cAMP metabolic processes as well as circadian rhythm in the dnBMAL1 mice at ZT10 compared to WT mice (Fig. 5b). Importantly, dnBMAL1 mice showed significant decreases in mRNA levels of adenylate cyclase (AC) 1 (a major AC in the brain), A-kinase Anchor Protein 5 (AKAP5), and DA receptors D1R and D5R (G-protein coupled receptors increasing cAMP level via AC) in the hippocampus at ZT10 compared to WT mice (Fig. 5c). Consistently, we observed reduced hippocampal cAMP levels in dnBMAL1 mice compared to WT mice (Fig. 5d). Thus our findings indicated impaired DA-cAMP-mediated signals in the hippocampus of dnBMAL1 mice.

### Activation of DA-cAMP signaling rescues retrieval deficits in dnBMAL1 mice

Taken together with previous findings that suggested the important roles of cAMP signaling pathways for memory retrieval^29^, we hypothesized that the decrease in cAMP production mediated by DA-cAMP-mediated signal transduction impairs retrieval (Fig. 5d). Therefore, we first asked whether increasing cAMP levels or activation of D1/5R would rescue the retrieval deficits at ZT10 in dnBMAL1 mice. WT and dnBMAL1 mice were trained at ZT10 and tested twice every 24 h (Tests 1 and 2) after the strong social recognition training. Prior to Test 1, mice received a micro-infusion of rolipram^30^ (a PDE4 inhibitor) into dorsal hippocampus (Fig. 5e, Supplementary Fig. 5A). This micro-infusion did not affect memory retrieval at Tests 1 and 2 in WT mice. However, dnBMAL1 mice treated with rolipram showed normal memory retrieval in the presence (Test 1), but not absence (Test 2), of rolipram infusion although these mutant mice treated with vehicle showed retrieval impairments at Tests 1 and 2 (Fig. 5e, Supplementary Fig. 5A). These observations indicate that rolipram rescued the retrieval deficit at ZT10 in dnBMAL1 mice. Furthermore, this micro-infusion of rolipram into dorsal hippocampus, but not into medial prefrontal cortex (mPFC), of WT mice rescued the retrieval deficit of weak social recognition memory at ZT10 (Fig. 5f, Supplementary Fig. 5B-D). A similar pattern of retrieval rescue at ZT10 was observed when dnBMAL1 mice were systemically injected with rolipram (Fig. 5g, Supplementary Fig. 5E). Importantly, pharmacological activation of D1/5R by systemic injection of D1/5R agonist (SKF38393) rescued the retrieval deficit in dnBMAL1 mice (Fig. 5g, Supplementary Fig. 5E). Conversely, the inactivation of D1/5 receptors by D1/5R antagonist (SKF83566) impaired the retrieval in WT mice at ZT10, but not at ZT4 (Fig. 5h, Supplementary Fig. 5F), supporting our conclusion that WT mice show impaired retrieval at ZT10 compared to at ZT4. Taken together, these observations suggest that retrieval deficits observed in dnBMAL1 (strong memory) and WT mice (weak memory) are mediated by impaired DA D1/5R-cAMP signal transduction.

### Phosphorylation of GluA1 S845 is required for enhancement of retrieval

**Fig. 6:**
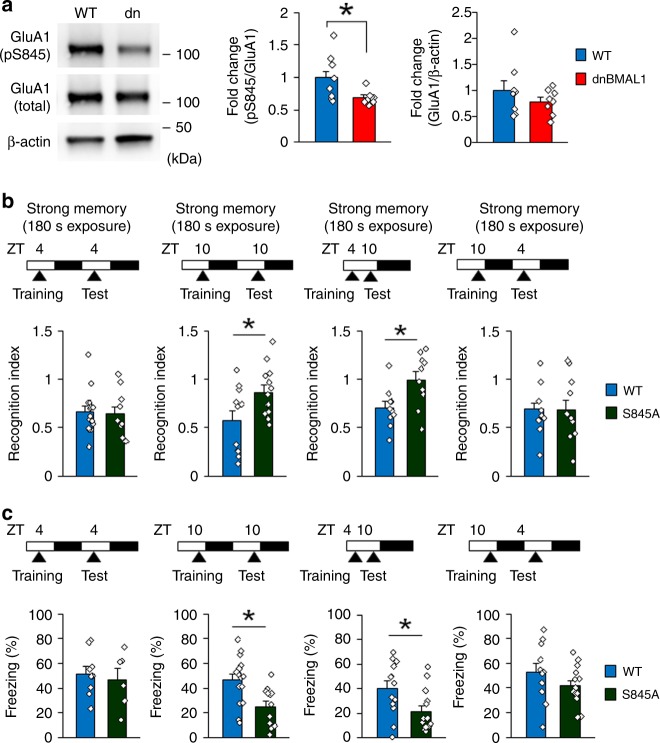
Phosphorylation of GluA1 S845 is required for memory retrieval. **a** Western blotting of the synaptosomal fraction from dorsal hippocampus of dnBMAL1 mice show significant reduction of phosphorylation level of GluA1 S845 (pS845). The graph represents fold changes compared to pS845 levels in WT. **b**, **c** Experimental designs are illustrated at the top of each panel. Memory retrieval is impaired at ZT10 in GluA1 S845A knockin (S845A) mice in **b** social recognition task (recognition index) and **c** contextual fear memory. dn dnBMAL1. All values are mean ± SEM. Individual data points are displayed as dots. *p < 0.05 as determined by one-way ANOVA with post hoc test. The results of the statistical analyses are presented in Supplementary Table 2. Source data are provided as a source data file.

Phosphorylation of the α-amino-3-hydroxy-5-methyl-4-isoxazolepropionicacid (AMPA) receptor subunit GluA1 at Serine 845 (S845) by cAMP-dependent protein kinase A (PKA) regulates trafficking of AMPA receptors and is suggested to play critical roles in learning and memory including memory retrieval^31^. Importantly, this phosphorylation is increased by the activation of D1/5R^32^. Therefore, we examined a role of phosphorylation at GluA1 S845 in memory retrieval. Interestingly, dnBMAL1 mice showed significant reductions of phosphorylated GluA1 S845 in the hippocampus at ZT10 compared to WT mice (Fig. 6a). Similar to dnBMAL1 mice, GluA1 S845A knockin mice^31^ showed impaired social recognition and contextual fear memories when tested at ZT10, but not at ZT4, whenever trained at ZT4 or at ZT10 (Fig. 6b, c, Supplementary Fig. 6), indicating that GluA1 S845A mutation causes retrieval deficit at ZT10. Taken together, our findings suggest that phosphorylation of GluA1 S845 by PKA is required for enhancement of memory retrieval and that therefore reduced phosphorylation of S845 by lowered activity of BMAL1 in the hippocampus impairs retrieval.

## Discussion

Circadian rhythm of gene expression controlled by transcription factor BMAL1/CLOCK is observed in the SCN, thought to be the master circadian clock in mammals^1,2,4,5^, and also in non-SCN regions^5–7^. Recent studies show that local peripheral circadian transcriptional clocks help regulate tissue-specific physiological and biological processes^1,8,9,14,21^. Although the results of several studies are consistent with the notion that circadian transcriptional clocks in the forebrain may also regulate circadian processes involved in memory^16–19,33–35^, functional roles of local brain circadian clocks in memory performance remain unclear. Here we show that fluctuations in forebrain (and in particular hippocampal) levels of the key clock transcription factor regulate retrieval. In WT mice, at times when, perhaps, BMAL1 activity is reduced (e.g., ZT10), memory retrieval is impaired following weak (but not strong) training. Artificially blocking BMAL1 function (by expressing a dnBMAL1) in forebrain impaired memory retrieval at all times of the day following weak training. These deficits were partially reversed when dnBMAL1 mice were trained with a strong protocol but only at time points when, perhaps, BMAL1 activity was elevated (e.g., ZT4). Furthermore, a similar pattern of retrieval deficit (at ZT10) was observed when dnBMAL1 is expressed specifically in the hippocampus. Together, these findings suggest that memory retrieval is regulated by a local circadian clock in the hippocampus.

**Fig. 7:**
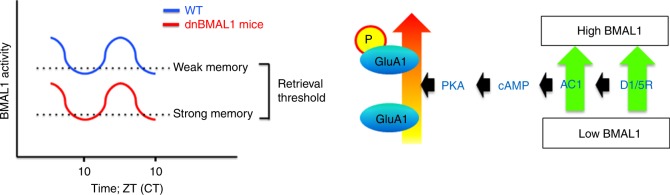
Model of circadian regulation of memory retrieval. Both WT and dnBMAL1 mice show a time-of-day retrieval profile. However, dnBMAL1 mice show worse retrieval performance at any time compared to WT mice; dnBMAL1 mice show impairments in retrieval of weak and strong memories at ZT4 and ZT10, respectively, compared to WT mice. Phosphorylation of GluA1 at S845 is required for maintenance of retrieval performance.

In humans, time-of-day can influence the cognitive processing, including memory retrieval^1^. While this effect has been recognized for over a century, the underlying neurobiological mechanisms are not understood. Strikingly, our findings suggest that the rates of BMAL1-mediated transcription determine retrieval success (Fig. 7). When BMAL1 activity is upregulated (e.g., at ZT4), retrieval is enhanced, whereas when BMAL1 activity are downregulated (e.g., ZT10) retrieval is impaired. These data identify a role for BMAL1 in memory processing and indicate that time-of-day effects on memory retrieval are mediated by local, hippocampal cell-autonomous oscillators.

Importantly, a previous study showed that WT mice could form long-term object recognition memory when trained only at ZT8–16 (but no long-term memory when trained at other ZTs), whereas BMAL1 conditional knockout (cKO) mice showed deficits of this type of memory at ZT4 and ZT16^34^. In contrast, our results indicated that dnBMAL1 mice showed retrieval deficit of object recognition memory at ZT10, but not ZT4, in experimental conditions where WT mice show normal recognition memory at both these time points. In order to reconcile these findings, future work should directly compare WT, dnBMAL1, and BMAL1 cKO mice using stronger and weaker training conditions. Such experiments will help to clarify time-of-day regulation of memory performance (memory formation vs retrieval) by local clocks (brain regions-specific roles).

The molecular mechanisms by which memory retrieval is regulated have remained unclear. Our studies indicate that BMAL1 modulates retrieval performance via regulation of DA/cAMP signaling. Our RNA-seq analyses identified downregulation of cAMP-mediated signaling-related gene expression levels in the hippocampus of dnBMAL1 mice. Consistently, dnBMAL1 mice show reduced AC1 mRNA, target of BMAL1/NPAS2 in the retina^36^, and cAMP levels in the hippocampus of dnBMAL1 mice at ZT10 when memory retrieval is impaired in these mutant mice (Fig. 3). Moreover, mRNA levels of D1R and D5R, activators of cAMP signals, are decreased in the hippocampus of dnBMAL1 mice. Importantly, pharmacologically restoring cAMP levels or D1/5R activities rescued retrieval deficits of strong memory observed in dnBMAL1 mice at ZT10 (following strong training), whereas similarly restoring cAMP levels rescued deficits in weak memory retrieval in WT mice at ZT10 (following weak training). Therefore, our findings suggest that hippocampal BMAL1 improves retrieval by activating DA-D1/5R-cAMP signaling in the hippocampus. These findings are consistent with previous observations that cAMP levels show circadian rhythms in hippocampus^16^ and that cAMP signaling pathway modulates memory retrieval^29^.

The SCN has been suggested to control hippocampal molecular signal transduction pathways including extracellular signal-regulated kinase signals required for memory performance and play critical roles in hippocampus-dependent learning and memory performance^37^. We found that dnBMAL1 mice showed worse retrieval performance than WT mice but still showed oscillation of retrieval performance. Therefore, SCN may control time-of-day changes in retrieval performance of hippocampus-dependent memories; the retrieval performance oscillation remains in dnBMAL1 mice since the function of SCN clock in these mutant mice is intact (Fig. 2).

The PKA signaling pathway regulates trafficking of AMPA receptors through phosphorylation of GluA1 at S845^31,38^. Abundant studies have shown importance of this S845 phosphorylation in synaptic plasticity including long-term potentiation, long-term depression, and homeostatic plasticity^31,39^. However, roles of GluA1 S845 phosphorylation in learning and memory still remain unknown at the behavioral levels. Here we show that dnBMAl1 mice show a decrease in phosphorylated GluA1 S845 and that more importantly GluA1 S845A knock-in mice exhibit similar memory retrieval deficits with dnBMAL1 mice. These findings suggest that retrieval is enhanced via GluA1 phosphorylation at S845 by PKA.

A recent study showed that tyrosine hydroxylase (TH) gene displays a time-of-day change in its expression, thereby leading to the oscillation of DA levels^40^. Levels of TH and DA levels are the lowest at circadian time 8–12, suggesting that the activity of DA signals show the weakness at this time zone^40^. Interestingly, we observed downregulation of mRNA levels of D1/D5R in the hippocampus of dnBMAL1 mice. These findings suggest that crosstalks between D1/D5R and cAMP signals controlled by hippocampal circadian clock have impacts on time-of-day regulation of retrieval.

In conclusion, we found time-of-day regulation of memory retrieval by hippocampal circadian clock. Importantly, BMAL1-mediated DA D1/D5R and cAMP signal transduction contributes to enhancement of retrieval by targeting GluA1 S845 phosphorylation.

## Methods

### Mice

Mice (male and female, at least 8 weeks of age) were housed in cages of 5 or 6, maintained on a 12 h light/dark schedule, and allowed ad libitum access to food and water in their home cages. GluA1 S845A knockin mice were obtained from Jackson Laboratory (stock number 012613)^41,42^. All of the experiments were conducted according to the Guide for the Care and Use of Laboratory Animals, Japan Neuroscience Society and Tokyo University of Agriculture. All the animal experiments were approved by the Animal Care and Use Committee of Tokyo University of Agriculture (authorization number: 280036). All surgical procedures were performed under Nembutal anesthesia, and every effort was made to minimize suffering. All of the experiments were conducted blind to the treatment condition and/or genotype of the mouse. Animal behavior was recorded using a video camera.

### Generation of transgenic mice

Transgenic mice were generated as described previously^43,44^. To introduce NotI restriction enzyme sites at the 5′ and 3′ end of the dnBMAL1 coding region, cDNA encoding dnBMAL1 was amplified by polymerase chain reaction (PCR) using pBS-dnBMAL1^22^ as a template and the following primers; forward primer, GCCGCCACCATGGGCGGCCGCATGTACCCATACGATGTTCCAGATTACGCTAGATCTATGGCGGACCAGAGAATGGA, and reverse primer, GGGGCGGCCGCGCTGACAAGCTTAGATCTTTACAGCGGCCATGGCAAGT. The NotI fragment encoding dnBMAL1 was subcloned into the NotI site of pcisTREmlu^43^, generating pTRE-dnBMAL1. TRE-dnBMAL1 was digested with MluI, and transgenic mice were generated by injecting the purified insert into the pronuclei of C57BL/6N zygotes. TRE-dnBMAL1 founders were crossed with C57BL/6N mice (Charles River Japan, Kanagawa, Japan). Their offsprings were crossed with mice (CaMKII-tTA mice) expressing the tTA transgene under the control of the αCaMKII promoter^24^, generating double transgenic mice (dnBMAL1 mice). Genotyping was performed using southern blotting and PCR. Specific probes for southern blotting of TRE-dnBMAL1 mice and CaMKII-tTA mice were derived from the 0.5-kbp NotI-NdeI fragment containing the TRE-promoter region from pcisTRE^43^ and the 0.6-kbp EcoRI-HindIII fragment encoding TetR region from pcDNA3-TetR-KRAB, respectively^43–48^. The genotyping primers for each transgenic mouse line are listed in Supplementary Table 1.

### Administration of Dox

TRE promoter-dependent transgene expression was regulated using the animal’s (dnBMAL1 and WT mice) drinking water containing 100 μg/mL Dox (Sigma-Aldrich, St Louis, MO, USA) dissolved in 5% sucrose to mask the bitter taste of Dox. To minimize potential effects of dnBMAL1 expression during development, mice were treated with Dox until 8 weeks of age (transgene OFF) at which time Dox was removed (transgene ON) to induce dnBMAL1 expression (dnBMAL1 OFF/ON mice). dnBMAL1 ON/OFF mice were not treated with Dox until 8 weeks of age (transgene ON) at which time Dox treatment was started (transgene OFF). dnBMAL1 ON/OFF/ON mice were treated with Dox from 4 to 8 weeks of age (see Supplementary Fig. 2B).

### RNA analyses

Total RNA was prepared from mouse whole-hippocampus region using the RNeasy Mini Kit (Qiagen, Valencia, CA, USA) or acid guanidinium thiocyanate-phenol-chloroform extraction method^43–48^. For reverse transcription-PCR (RT-PCR) analyses, total RNA (1 μg) was reverse transcribed with an oligo dT primer. The PCR reactions were carried out for 35 cycles (a 1-min cycle at each of the following temperatures: 94, 60, and 72 °C). We used glyceraldehyde 3-phosphate dehydrogenase (GAPDH) as an internal control. The dnBMAL1 transgene contains the 3′-end of BMAL1 open reading frame and the 3′-untranslated region (3′-UTR) from pTRE-dnBMAL1 that produces dnBMAL1 mRNA-specific PCR products. The PCR products were analyzed by electrophoresis on agarose gels stained with ethidium bromide. The primer sequences for RT-PCR analyses are listed in Supplementary Table 1.

To detect dnBMAL1 mRNA and arginine vasopressin (*Avp*) mRNA, double-label fluorescence in situ hybridization (FISH) analysis was performed according to a previously described protocol^43,44,49–51^ with a few modifications. *Avp* was used as a marker for the dorsolateral SCN^52,53^. To generate a specific riboprobe for TRE promoter-dependent transcripts encoding dnBMAL1, the 0.3-kbp HindIII-XbaI fragment containing the 3′-UTR from pTRE-dnBMAL1 was cloned into pBS (pBS-TRE 3′-UTR). To generate a riboprobe for *Avp* mRNA, *Avp* cDNA (cds, 21–495) was amplified by RT-PCR using mouse brain cDNA as a template (and with the following primers: *Avp* forward, GGGAAGCTTCACTACGCTCTCCGCTTGTT; *Avp* reverse, GGGTCTAGAGGGCTTGGCAGAATCCA). The resulting RT-PCR fragments were subcloned into the HindIII-XbaI sites of pBluescript II (SK-) (Agilent Technologies, Santa Clara, CA, USA).

Digoxigenin- or fluorescein-labeled riboprobes were generated using commercially available transcription kits (Ambion MaxiScript T3/T7 Kit; Ambion, Austin, TX, USA) and RNA labeling mixes (Roche Diagnostics, Mannheim, Germany). Sections were hybridized at 56 °C with antisense or sense probes overnight. FISH signal of the fluorescein-labeled dnBMAL1-specific riboprobe was detected with anti-fluorescein horseradish peroxidase (HRP) conjugate (PerkinElmer, Boston, MA, USA) and amplified with a cyanine-3 substrate kit (Cy3 TSA Plus; PerkinElmer). After quenching with H_2_O_2_, FISH signal of the digoxigenin-labeled *Avp* riboprobe was detected with anti-digoxigenin-POD, Fab fragments (Roche) and amplified with a cyanine-5 substrate kit (Cy5 TSA Plus, PerkinElmer). Slides were enclosed with Vectashield mounting medium containing 4,6-diamidino-2-phenylindole (DAPI; Vector Laboratories, Burlingame, CA, USA) for nuclear counterstaining.

Quantitative RT-PCR (qRT-PCR) was performed as described previously^24,44–47^. qRT-PCR was performed in the QuantStudio 3 Real-Time PCR system (Thermo Fisher Scientific, Rockford, IL, USA) using the Power SYBR Green PCR Master Mix (Thermo Fisher Scientific) according to the manufacturer’s protocol. The reaction was first incubated at 50 °C for 2 min, then at 95 °C for 10 min, followed by 40 cycles of 95 °C for 15 s and 60 °C for 1 min. Amplification of a single PCR product was confirmed by monitoring the dissociation curve and electrophoresis on agarose gels stained with ethidium bromide. Amplification curves were visually inspected to set a suitable baseline range and threshold level. The relative quantification method was employed for the quantification of target molecules according to the manufacturer’s protocol, where the ratio between the amount of each target molecule and a reference molecule within the same sample was calculated. All measurements were performed in triplicate. The levels of GAPDH mRNA were used to normalize the relative expression levels of target mRNA. The primer sequences for qRT-PCR analyses are listed in Supplementary Table 1.

### RNA-sequencing

Total RNA from hippocampus was isolated using the RNeasy Mini Kit (Qiagen) according to the manufacturer’s instructions. They were subjected to RNA-seq analysis for each experimental group as biological triplicates (*n* = 3). After RNA quality check (all RIN values > 8.2) with the RNA nano kit (Agilent Technologies) on Agilent Bioanalyzer (Agilent Technologies), 1 μg of total RNA were used for preparing cDNA libraries with the TruSeq RNA Sample Preparation Kit v2 (Illumina, San Diego, CA, USA), following the manufacturer’s instructions. The derived cDNA libraries were analyzed on an Agilent Bioanalyzer with DNA 1000 Kit and quantified by qPCR using the KAPA Library Quantification Kit (KAPA Bio systems, Wilmington, MA, USA). Twelve libraries with a unique barcode were pooled in three lanes and clusters were generated on a cBot (Illumina) to obtain 100 bp single reads in a HiSeq 2500 sequencer (Illumina). Generation of demultiplexed fastq files was performed using bcl2fastq ver. 2.18 (Illumina) and 385 million sequence reads were obtained in total. Filtering, mapping, and differential expression analysis were performed using the CLC Genomics Workbench software ver. 9.5 (Qiagen). The raw sequence reads were filtered to exclude adapter sequences, ambiguous nucleotides, and low-quality sequences and the retained sequences were aligned against the mouse genome (mm10).

Predictive causal analysis was performed using the IPA software (Ingenuity, Redwood City, CA, USA). Differentially expressed genes’ list (genes with <−1.1-fold changes) was imported to IPA analysis and subjected to a core analysis. Pathway enrichment was examined through Fisher's exact test.

GSEA was performed using GSEA ver. 2.0.3^54,55^ for statistical analysis of changes in the gene expression patterns. The gene set collections C2 (curated gene sets: 4731 gene sets) and C5 (GO gene sets: 5917 gene sets) were obtained from Molecular Signature Database (MSigDB) v6.0 [http://software.broadinstitute.org/gsea/msigdb/collections.jsp]. The gene expression values were calculated as read per kilobase of exon per million mapped reads (RPKM)^56^. Expressed genes (RPKM > 1) were ranked according to their RPKM values and imported as input file. Gene sets were considered to be highly enriched if *p* value was <0.05 and false discovery rate-corrected *q* value was <0.25^57^.

RNA sequencing data have been deposited to the DDBJ Sequence Read Archive (DRA) and are available at the accession number DRA006214.

### Chromatin immunoprecipitation (ChIP)

ChIP assays were performed according to a protocol from the Merck Millipore Chromatin Immunoprecipitation Assay Kit (ChIP Assay Kit, catalog number 17–295; Merck Millipore, Darmstadt, Germany) with minor modifications^58–61^. Dissected tissues of hippocampus were minced into 1-mm pieces, immediately frozen on dry ice, and then stored at −80 °C. To generate crosslinks of the protein–DNA complexes, tissues were placed in 1% formaldehyde for 10 min at room temperature. Reaction was quenched by adding glycine at a final concentration of 0.125 M. The tissue was washed three times with ice-cold phosphate-buffered saline (PBS)-containing protease inhibitors (Complete Tab, Roche Diagnostics) and then homogenized in ice-cold PBS using glass homogenizer. The homogenate was centrifuged at 1000 × *g* for 5 min. The pellet was dissolved in the cell lysis buffer [10 mM Tris, 10 mM NaCl, and 0.2% IGEPAL CA-630 (Sigma-Aldrich)], placed for 15 min, and then centrifuged at 1000 × *g* for 5 min. The pellet was dissolved in the sodium dodecyl sulfate (SDS) lysis buffer [1% SDS, 10 mM EDTA, 50 mM Tris, pH 8.1, (Merck Millipore)] and then placed for 10 min. The extracted chromatin was sheared to 200–1000 bp using the bioruptor UCD-250 (Cosmo Bio, Tokyo, Japan); each sample was sonicated at maximal power setting for 40 30-s pulses (30-s pause between pulses) at 4 °C while samples were immersed in an ice bath. Lysates were centrifuged at 20,000 × *g* for 10 min to remove insoluble material and then diluted with ChIP dilution buffer [0.01% SDS, 1.1% Triton X-100, 1.2 mM EDTA, 16.7 mM Tris-HCl, pH 8.1, 167 mM NaCl, (Merck Millipore)] to a final volume of 1 mL. One hundred microliters of the pre-immunoprecipitated lysates were saved as input for later normalization. The resulting lysates was precleared for 30 min at 4 °C using Salmon Sperm DNA/Protein A agarose Slurry (Merck Millipore) and then immunoprecipitated for 48 h at 4 °C using 5 μg of anti-CLOCK antibody (ab3517, Abcam, Cambridge, UK) or anti-rabbit IgG (ab46540, Abcam). Chromatin–antibody complexes were collected by centrifuge and sequentially washed with low salt, high salt, LiCl, and then TE buffers. Chromatin was eluted using NaHCO_3_/SDS buffer (1% SDS, 0.1 M NaHCO_3_). ChIP and input samples were incubated in high-salt conditions at 65 °C overnight for crosslink reversal, treated with proteinase K, and then purified using phenol/chloroform method. Purified DNA samples were subjected to qPCR analyses. Clock exon 6 region was used as a negative control that does not contain functional E-box sequences. The primer sequences for ChIP-qPCR analyses are listed in Supplementary Table 1.

### Cyclic AMP measurement

Dissected tissues of hippocampus were immediately frozen on dry ice and stored at −80 °C until use. Tissue was lysated in 0.1 M HCl. cAMP content was measured using the enzyme-linked immunosorbent assay-based assay kit (ENZO Life Sciences, Farmingdale, NY, USA) following the manufacturer’s instructions.

### Tissue preparation and western blotting

Mouse brains were sliced using a Rodent Brain Matrix (RBM-2000; ASI Instruments, Warren, MI, USA). The dorsal hippocampus (bregma between −1.06 and −2.06 mm) was dissected from the slices and stored at −80 °C. To analyze the levels of phosphorylated GluA1 at Ser845, synaptic membrane fractions were isolated on a discontinuous sucrose gradient^22^. Brain tissues were homogenized in ice-cold homogenization buffer (4 mM HEPES, pH 7.4; 320 mM sucrose) containing a protease inhibitor mixture (Complete; Roche Diagnostics). The homogenized samples were centrifuged twice at 500 × *g* at 4 °C for 2 min to remove nuclei and other debris. The supernatant was centrifuged at 20,000 × *g* at 4 °C for 30 min. The pellets were suspended in a SDS–polyacrylamide gel (SDS-PAGE) loading buffer and analyzed by western blotting. To analyze the expression levels of BMAL1, Per2, and Dbp, brain tissues were homogenized in a SDS-PAGE loading buffer and then centrifuged at 15,000 × *g* at 4 °C for 30 min. The supernatants were analyzed by western blotting. Western blotting was performed using a rabbit polyclonal anti-GluA1 antibody (AB1504, 1:1000; Merck Millipore), anti-GluA1 phospho-Ser845 antibody (AB5849, 1:1000; Merck Millipore), anti-BMAL1 antibody (NB100-2288, 1:1000; Novus Biologicals, Littleton, CO, USA), anti-Per2 antibody (PER21-A, 1:1000; Alpha Diagnostic International, San Antonio, TX, USA), anti-Dbp antibody (PM079, 1:1000; MBL), or mouse monoclonal anti-β-Actin antibody (A1978, 1:10,000; Sigma-Aldrich)^22,62^. Positive antibody binding was visualized using an ImmunoStar LD system (Wako, Japan). Densitometric analysis was performed by the Image Lab software (Bio-Rad) after scanning (ChemiDoc XRS+ system, Bio-Rad). The phosphorylation levels of GluA1 were calculated by normalizing the levels of phosphorylated GluA1 at Ser845 to the total amount of GluA1 (relative phospho-GluA1 [Ser845]/GluA1 levels). Each experiment was independently repeated at least three times. Uncropped images are shown in the Source data file.

### Behavioral experiments

Before behavioral experiments began, mice were individually handled for 2 min each day for 1 week. Behavioral experiments were conducted in an illuminated testing room during the light phase of the cycle or under dim red light conditions (light intensity 4 lux) during the night phase or D/D conditions. Individual mice were used for all experiments. Behavioral experiments were performed as described previously^22,28,44–48,63–70^.

### Social recognition task

The social recognition task was performed as previously described^20,44,46,48,65,70^. Briefly, adult mice were placed into individual plastic cages in an experimental room. The cages were identical to those in which mice were normally housed (plastic, 30 cm × 17 cm × 12 cm). After a period of 60 min, a juvenile mouse was placed into a cage with the adult mouse for a training trial that lasted for 2 min (weak training conditions; Figs. 1b, 3a, and 5f, Supplementary Figs. 1E, 3A, and 5B–D) or 3 min (strong training conditions; Figs. 3b–e, 4d, and 5e, g, h, Supplementary Figs. 1A–D, 3B–I, 4E, and 5A, E, F). The duration of the adult’s social investigation behavior was quantified using a stopwatch. Social investigation was defined as described previously^71^. Memory for social recognition was assessed at different periods of time after initial training (as indicated in each figure). The amount of time the adult subject mouse investigated the same juvenile mouse was recorded (Test 1). When Test 2 was performed, memory was reassessed at the time as indicated after Test 1. To evaluate the differences in the ability to form and retrieve social recognition memory between the groups of mice, we calculated a recognition index (defined as the ratio of the duration of the second or third and first investigation times). For experiments during D/D conditions, L/D-entrained mice were placed into D/D conditions for 3 days.

To examine an effect of increased cAMP levels on memory retrieval, mice were trained at ZT10 using strong (Fig. 5e, g, Supplementary Fig. 5A, E) or weak (Fig. 5f, Supplementary Fig. 5B–D) training conditions and tested twice every 24 h (Tests 1 and 2) after the training. Mice received a systemic injection of rolipram (0.1 mg/kg, intraperitoneal (i.p.)) or micro-infusion into hippocampus or mPFC (hippocampus, 4 μg/side; mPFC, 4 μg) 30 min before Test 1. Rolipram (Tocris Bioscience) was dissolved in dimethyl sulfoxide (DMSO; Wako) and then diluted with distilled water.

To examine an effect of activation or inactivation of DA 1/5 receptor on memory retrieval, mice were trained at ZT10 using strong (Fig. 5e, g, Supplementary Fig. 5A, E) training conditions and tested twice every 24 h (Tests 1 and 2) after the training. Mice received a systemic injection of SKF38393 (8 mg/kg, i.p.) or SKF83566 (0.15 mg/kg, i.p.) 30 min before Test 1. SKF38393 (Tocris Bioscience) and SKF83566 were dissolved and diluted with distilled water.

Comparisons of social investigation times within each group among training and tests are shown in Supplemental information.

### Novel object recognition task

On 3 consecutive days, mice were individually habituated to an open field (40 cm × 40 cm × 40 cm) for 20 min in the absence of objects. For the training and test sessions, two objects were placed in the open field, and the mouse was allowed to explore for 15 min. The training and tests were performed at the times as indicated in each figure. For Test 1, mice were re-exposed to one of the familiar objects and a novel object. When Test 2 was performed, mice were re-exposed to the familiar object used in training and Test 1 together with a novel object (that was not used in training and Test 1). The time spent exploring each object during the training and test sessions was recorded by an observer. Exploration of the object was defined as occurring when the head of the mouse was facing and within 1–2 cm of the object or when any part of the body, excluding the tail, was physically touching the object. The objects were matched for size, weight, and innate interest levels based on preliminary tests with WT mice and were thoroughly cleaned between trials to make sure no olfactory cues were present. Memory was operationally defined as the proportion of time mice spent investigating the novel object minus the proportion spent investigating the familiar one. Discrimination Index, DI = (novel object exploration time/total exploration time) − (familiar object exploration time/total exploration time)^72,73^.

### Contextual fear conditioning task

Mice were trained and tested in conditioning chambers (17.5 cm × 17.5 cm × 15 cm) fitted with a stainless-steel grid floor through which footshocks could be delivered^28,45,46,48,63,66–68^. Training consisted of placing the mice in the chamber and delivering a shock (2 s duration, 0.4 mA) 148 s later. Mice were returned to the home cage 30 s after the shock. Memory was measured as the percentage of time spent freezing during 5 min when replaced in the training context (test). Freezing behavior (defined as the complete lack of movement, except for respiration) was measured automatically as described elsewhere^74^ (O'HARA & CO. LTD., Tokyo, Japan).

### Surgery for drug micro-infusion

Under Somnopentyl (50 mg/kg, i.p., Kyoritsu Seiyaku Corporation, Tokyo) anesthesia and using standard stereotaxic procedures, a stainless-steel guide cannula (22 gauge) was implanted into the dorsal hippocampus or mPFC^28,44,48,64,66,75–78^. Stereotaxic coordinates for dorsal hippocampus or mPFC placement based on the brain atlas of Franklin and Paxinos (1997) were as follows: dorsal hippocampus (−1.8 mm, ±1.8 mm, −1.9 mm) or mPFC (2.7 mm, ±0 mm, −1.6 mm). Mice were allowed to recover for at least 1 week after surgery. Ten minutes before the Test of social recognition task, mice received 0.5 μL infusions of either lidocaine (4%; Sigma-Aldrich) or vehicle (PBS) at a rate of 0.2 μL/min (Supplementary Fig. 1A, B). Thirty minutes before Test 1 of social recognition task, mice received 0.4 μL infusions of either rolipram (10 μg/μL; Tocris Bioscience) or vehicle (DMSO) at a rate of 0.2 μL/min (Fig. 5e, f, Supplementary Fig. 5A–D). The injection cannula (28 gauge) was left in place for 2 min after the infusion and the mice were returned to their individual plastic cages before testing. Only mice with a cannulation tip within the boundaries of the hippocampus or mPFC were included in the data analysis.

### Circadian rhythms of locomotor activity during LD and DD cycles

Mice were placed in individual cages under a fixed 12 h:12 h light–dark (LD12:12) schedule after habituation for a week before recording. After 1 week on LD12:12, the mice were released into constant darkness (DD) for 2 weeks. Locomotor activity was monitored with an infrared locomotor recording apparatus (Biotex, Kyoto, Japan) by recording activity in 10-min bins. To estimate free-running periods in DD, a χ^2^ periodogram analysis was performed using 14 days of data in DD^79,80^. Daily locomotor activity was presented as the average of locomotor activity for 7 days under LD conditions.

### AAV experiments

To generate an AAV vector that bicistronically expresses EGFP protein and dnBMAL1 using a self-cleaving 2A peptide sequence (Thoseaasigna virus 2A; T2A), the BglII fragment encoding dnBMAL1 from pTRE-dnBMAL1 was subcloned into the BglII site of pAAV-cDNA6-EGFP-T2A-V5His (Vector Biorabs, Philadelphia, PA), generating pAAV-CMV-EGFP-T2A-dnBMAL1. AAV9-CMV-EGFP-T2A-dnBMAL1 (titer 5.6 × 10^13^ genome copies (gc)/mL) and control AAV9-CMV-EGFP (6.4 × 10^13^ gc/mL) vectors were produced and purified by Vector Biolabs (Philadelphia, PA, USA).

Viral knockdown of BMAL1 was performed using four BMAL1 short hairpin RNA (shRNA; mouse BMAL1) constructed and packaged by Vigene Biosciences (Rockville, MD, USA). The four BMAL1 shRNA were driven by either H1 or U6 promoter individually in one expression vector and were packaged in AAV9 (1.0 × 10^13^ gc/mL) labeled with EGFP (driven by a CMV promoter). The following four shRNA sequences were employed: BMAL1_sh1, GGACGAACTGAAACACCTAATTCTCTTCAAGAGAGAGAATTAGGTGTTTCAGTTCGTCTTTTTT; BMAL1_sh2, CCGACCCTCATGGAAGGTTAGAATATTCAAGAGATATTCTAACCTTCCATGAGGGTTTTTTT; BMAL1_sh3, GACATAGGCATCGATATGATAGTTCAAGAGACTATCATATCGATGCCTATGTTTTTTT; BMAL1_sh4, GTCTTCAAGATCCTCAATTATATTCAAGAGATATAATTGAGGATCTTGAAGATTTTTT. A commercially available scramble shRNA-expressing EGFP was used as a control (Vector Biolabs).

For virus injections, the stereotaxic injection of AAV vectors was performed in a biological safety cabinet. Mice were anesthetized with Somnopentyl (50 mg/kg, i.p., Kyoritsu Seiyaku Corporation) and placed in a stereotaxic frame. The skull was exposed and a small portion of the skull over dorsal hippocampus was removed bilaterally with a drill. AAVs (0.5 μL/site at a speed of 0.1 μL/min) were injected into the mouse brains using glass capillary pipettes [The anteroposterior (A/P) −1.6 mm, mediolateral (M/L) ±1.6 mm, dorsoventral (D/V) (from Dura) −1.6 mm] pulled with a micropipette puller (P-87, Sutter Instruments, Novato, CA, USA). After the injection, the glass pipettes were left in place for another 5 min before being slowly lifted up and removed. The mice were sutured, and antibiotic ointment was applied. Mice were then kept on a warm heater for recovery. Mice were allowed to recover for 3 weeks after surgery and then subjected to behavioral and immunohistochemical (IHC) analyses. Successful transduction of the hippocampus region was confirmed histologically by native EGFP fluorescence. Only mice showing bilateral EGFP expression in the hippocampus were included in subsequent data analyses.

### Immunohistochemistry

IHC was performed as described previously^22,28,65–70,76–78,81^. Mice were transcardially perfused with 4% paraformaldehyde. Brains were then removed, fixed overnight, transferred to 30% sucrose, and stored at 4 °C. Coronal sections (30 μm) were cut in a cryostat. Sections were pre-treated with 1% hydrogen peroxide. Sections were incubated overnight with the primary antibody, including rabbit polyclonal anti-c-fos (ABE457, 1:1000; Merck Millipore), rabbit polyclonal anti-PER2 (PER21-A, 1:1000; Alpha Diagnostic International, San Antonio, TX, USA), rabbit polyclonal anti-DBP (LS-B3479, 1:1000; Lifespan Bioscience, Seattle, WA, USA), or mouse anti-NeuN (MAB377, 1:500; Merck Millipore). Sections were then incubated with HRP-conjugated secondary antibody (711-036-152, 1:500; Jackson Immunoresearch Laboratories, West Grove, PA, USA) for c-fos and NeuN, biotinylated secondary antibody (711-066-152, 1:500; Jackson Immunoresearch Laboratories) for PER2 and DBP (Fig. 2, Supplementary Fig. 2), or goat anti-rabbit IgG secondary antibody, Alexa Fluor® 555 conjugate (A-21428, 1:500; Thermo Fisher Scientific) for PER2 (Supplementary Fig. 4D) for 60 min at 20 °C. Signals of PER2 and DBP were amplified using the Vectastain Elite ABC Kit (Vector Laboratories), tyramide signal amplification (Fig. 2, Supplementary Fig. 2). Sections incubated with HRP-conjugated or biotinylated secondary antibody were visualized using Alexa-Fluor568-conjugated streptavidin (S11226; Invitrogen, Eugene, OR, USA). Sections were mounted on slides with Permafluor antifade medium (Merck Millipore) or Vectashield mounting medium containing DAPI (Vector Laboratories) (Fig. 2a, Supplementary Figs. 2D and 4D).

### c-fos IHC: contextual fear conditioning

Mice were contextual fear conditioned as described above. The no-conditioned group (conditioned stimulus only) received a training session with no shock. All mice were re-exposed to the contextual chamber for 3 min at the time as indicated after training. c-fos-positive cells were analyzed at 90 min after the re-exposure^22,28,66,67,78^ using a polyclonal rabbit primary antibody for anti-c-fos.

### Microscopy

Fluorescence images were acquired using a confocal microscope TCS SP8 (Leica, Wetzlar, Germany) with a ×10 objective (FISH) or FV300 (Olympus, Tokyo, Japan) with a ×20 objective (IHC) or a fluorescence microscope (BZ-X710; Keyence, Osaka, Japan). Confocal 2-μm *z*-stack images were obtained using the LAS AF software (Leica). Equal cutoff thresholds were applied to all slices. DBP and PER2 signal intensity were calculated using the ImageJ software (National Institute of Health). The number of c-fos immunoreactive cells in the field of view within the each brain region across at least two sections were counted using the WinROOF version 5.6 software (Mitani Corporation, Fukui, Japan)^22,28,48,65–68,70,75–78^.

### Northern blotting

Northern blot analysis using a ^32^P-labeled SV40 poly(A) probe was performed^43–45,48^. The filters were hybridized with a SV40 poly(A) probe and then re-hybridized with β-actin cDNA as an internal control.

### Open field test

Mice were placed into the center of a square open field chamber (50 cm long × 50 cm wide × 40 cm high) that was surrounded by walls. The total length of the path the mouse traveled (Total distance) and the time it spent in a center square (30 cm × 30 cm; % center) were measured over the course of 5 min using an automatic monitoring system (O'Hara Co., Ltd., Tokyo, Japan)^47^.

### Elevated zero maze test

The zero maze consisted of a circular path (5.5 cm width, inner diameter of 25 cm) that had two open and two closed sections (wall was 15 cm high) and was elevated 50 cm above the floor. Mice were initially placed in the closed section and their behavior was observed for 5 min. The length of time that they spent in the open section (Time in open section) and the number of times they entered into the open section with four paws (number of entries into open section) were measured^47^.

### Pain sensitivity test

Mice were placed into the conditioning chamber, and a scrambled electric footshock of rising intensity (starting from 0 mA) was applied^82^. The shock was switched off as soon as the mice jumped. The corresponding shock intensity was defined as pain threshold.

### Data analysis

Data were analyzed with factorial or repeated-measures analysis of variance (ANOVA). One-, two-, or three-way ANOVA followed by post hoc Newman–Keuls or post hoc Bonferroni’s comparisons were used to analyze the effects of groups, genotypes, times, drugs, and retrievals. All values in the text and figure legends represent mean ± SEM. A paired *t* test was used to analyze differences of social investigation times within each group between first and second exposures in the social recognition test.

### Reporting summary

Further information on research design is available in the Nature Research Reporting Summary linked to this article.

## Supplementary information

Supplementary Information

Peer Review

Reporting Summary

## Data Availability

The data supporting the findings of this study are available from the corresponding author upon reasonable request. The source data underlying Figs. 1–6 and Supplementary Figs. 1–6 are provided as a Source Data file.

## Source Data

Source Data
