# Supplementary material for: Hippocampal clock regulates memory retrieval via Dopamine and PKA-induced GluA1 phosphorylation
Source: Nat Commun. 2019 Dec 18;10:5766. doi: 10.1038/s41467-019-13554-y (PMC6920429; doi:10.1038/s41467-019-13554-y)
Supplement: Supplementary file 1 — Supplementary Information [file 41467_2019_13554_MOESM1_ESM.pdf]

Supplementary Information

Hippocampal clock regulates memory retrieval via Dopamine and PKA-induced GluA1 phosphorylation

Hasegawa et al.,

This PDF file includes:

1. **Supplementary Figure 1.** Inactivation of hippocampus blocks retrieval of social recognition memory, related to Figure 1.
2. **Supplementary Figure 2.** Dox-dependent regulation of dnBMAL1 expression in forebrain of dnBMAL1 mice and characterization of these mutant mice, related to Figure 2.
3. **Supplementary Figure 3.** dnBMAL1 expression-dependent deficits in retrieval of social recognition memory, related to Figure 3.
4. **Supplementary Figure 4.** dnBMAL1 expression-dependent deficits in retrieval of hippocampus-dependent memory, related to Figure 4.
5. **Supplementary Figure 5.** Rolipram or D1/5R agonist (SKF38393) rescues retrieval deficit in dnBMAL1 and WT mice at ZT10 under strong or weak, respectively, training condition, related to Figure 5.
6. **Supplementary Figure 6.** Phosphorylation of GluA1 S845 is required for memory retrieval, related to Figure 6.
7. **Supplementary Table 1.** List of all primer sequences used in genotyping, RT-PCR, qRT-PCR and ChIP assay.
8. **Supplementary Table 2.** Statistics and Sample Sizes.

Supplementary Figure 1.

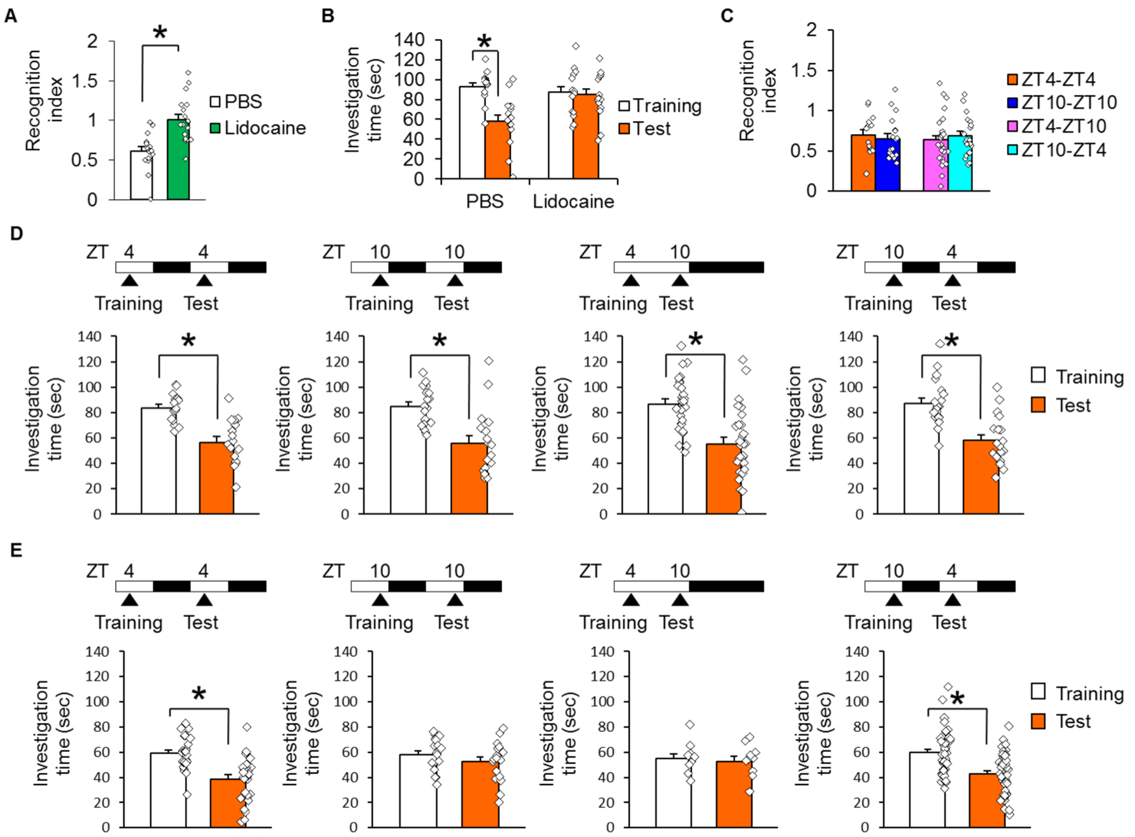

Supplementary Figure 1. Inactivation of hippocampus blocks retrieval of social recognition memory, related to Figure 1.

(A, B) Hippocampus-dependent retrieval of social recognition memory. Micro-infusion of lidocaine into the dorsal hippocampus before Test impaired social recognition memory. Memory retrieval is tested 24 h after the training. (A) Recognition index. Significant effect of Drug. (B) Investigation time. Lidocaine ($p > 0.05$), but not PBS ($p < 0.05$), group fails to reduce investigation time.

(C, D) WT mice show memory retrieval following strong training (3 min exposure during training). (C) Recognition index. (D) Investigation time. Experimental designs are illustrated at top of each panel. All groups significantly reduce investigation time ($p < 0.05$).

(E) Investigation time in Figure 1B; Memory retrieval is impaired in WT mice at ZT10 under weak training condition. WT mice show memory retrieval at ZT4, but not at ZT10, following weak training (2 min exposure during training). Mice tested at ZT4, but not at ZT10, significantly reduce investigation time ($p < 0.05$). All values are mean ± SEM. Individual data points are displayed as dots. * $p < 0.05$ as determined by

two-way (C) or one-way (A) ANOVA with a post hoc test, paired t test (B, D, E). The results of the statistical analyses are presented in Supplementary Table 2. Source data are provided as a source data file.

Supplementary Figure 2.

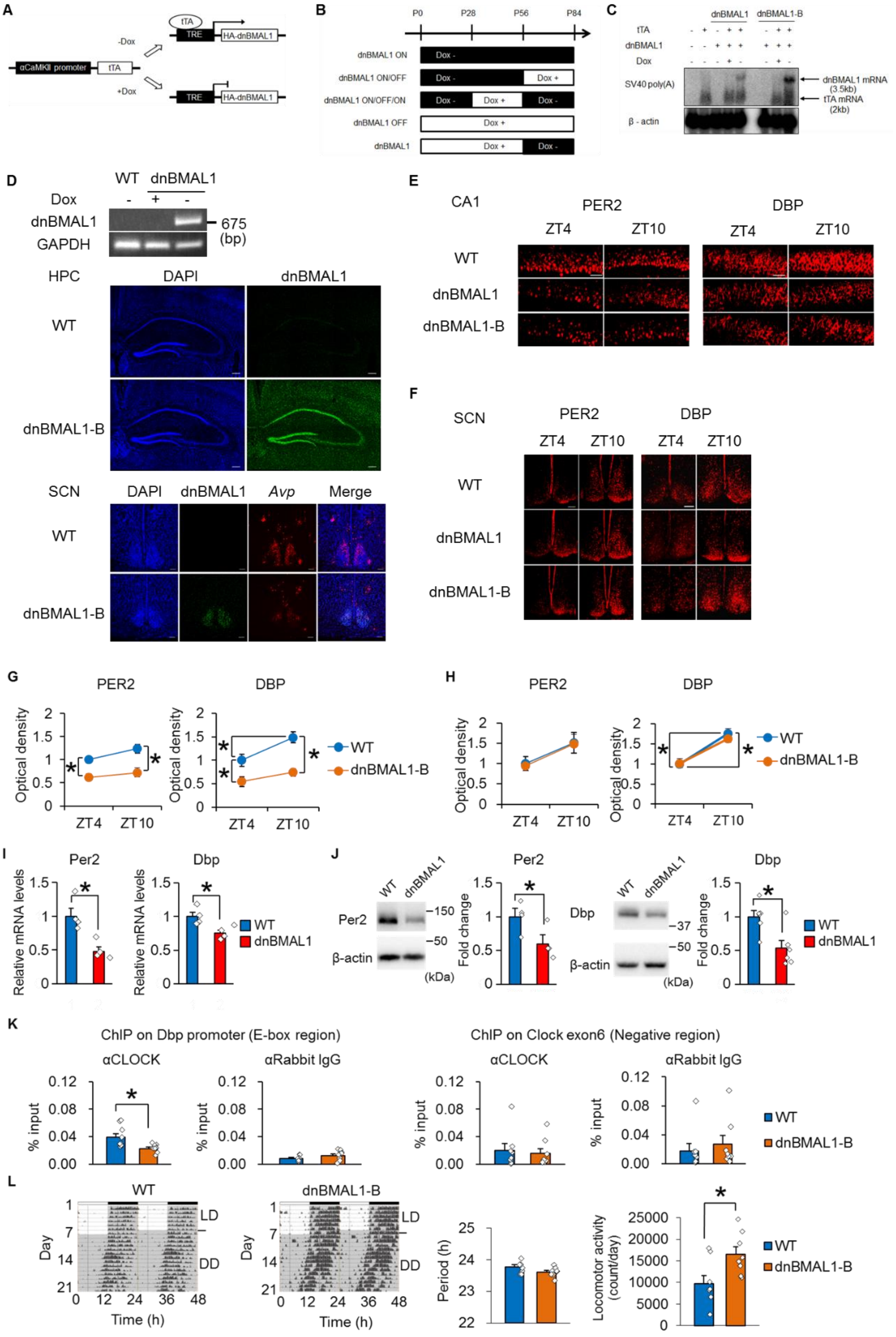

Supplementary Figure 2. Dox-dependent regulation of dnBMAL1 expression in forebrain of dnBMAL1 mice and characterization of these mutant mice, related to Figure 2.

(A) Schematic representation of Dox-dependent regulation of dnBMAL1 expression in forebrain.

(B) Schedule for treatment of Dox.

(C) Dox-dependent expression of dnBMAL1 mRNA in forebrain (excluding SCN) in dnBMAL1 and Line-B ON and OFF mice. Northern blot analyses are performed using SV40 poly(A) probe that enables to detect both dnBMAL1 and tTA mRNAs. Expression of dnBMAL1 mRNA in dnBMAL1-B mice is higher than that in dnBMAL1 mice.

(D) Expression of dnBMAL1 mRNA in dnBMAL1-B mice. Dox-dependent expression of dnBMAL1 mRNA in hippocampus and SCN of dnBMAL1-B mice. (Upper panel) Dox-dependent expression of dnBMAL1 mRNA is observed in hippocampus of dnBMAL1-B mice (RT-PCR). dnBMAL1 mRNA expression is observed in hippocampus (HPC, middle panel) of dnBMAL1-B mice (fluorescence *in situ* hybridization). In contrast to dnBMAL1 mice, weak expression of dnBMAL1 mRNA is observed in SCN of dnBMAL1-B mice (lower panel**)**. Expression of AVP mRNA was used as a marker of SCN. DAPI (blue, nuclear stain), dnBMAL1 mRNA (green), AVP mRNA (red). Scale bar, 200 μm (HPC) and 100 μm (SCN).

(E and F) Representative images of PER2 (left panels) and DBP (right panels) expressions in CA1 region of hippocampus (E) and SCN (F) in WT, dnBMAL1 and dnBMAL1-B mice, related to Figure 2B and C. Scale bar, 50 μm (CA1) and 100 μm (SCN).

(G and H) Quantification of PER2 (left panels) and DBP (right panels) expression in CA1 region of hippocampus (G) and SCN (H). (G) Expressions of PER2 and DBP are reduced in CA1 region of hippocampus in dnBMAL1-B mice at ZT4 and ZT10. The graph represents fold changes compared to expression levels in WT at ZT4. The data of WT mice are same with those used in Figure 2B. (H) Expressions of PER2 and DBP are normal in SCN of dnBMAL1-B mice at ZT4 and ZT10. The graph represents fold changes compared to expression levels in WT at ZT4. The data of WT mice are same with those used in Figure 2C.

(I) Quantification of Per2 and Dbp expression in the hippocampus (quantitative RT-PCR). Expressions of Per2 and Dbp are reduced in the hippocampus in dnBMAL1 mice at ZT10. The graph represents fold changes compared to expression levels in WT at ZT10.

(J) (Left panel) Representative images of Per2 and Dbp expressions in the hippocampus of WT and dnBMAL1 mice (western blotting). (Right panels) Quantification of Per2 and Dbp expressions. One-way ANOVA with group reveals significant decreases of Per2 and Dbp expressions in the hippocampus of dnBMAL1 mice at ZT10 compared to WT mice. The graph represents fold changes compared to expression levels in WT at ZT10.

(K) dnBMAL1 blocks the binding of CLOCK to Dbp promoter region in hippocampus of dnBMAL1-B mice at ZT10. Anti-CLOCK antibody, but not anti-Rabbit IgG, precipitates DBP promoter region although DNA regions not containing E-box (exon 6 of *clock* gene) are comparably precipitated by anti-CLOCK antibody and anti-Rabbit IgG.

(L) (Left panel) Representative activity records (actograms) indicate that circadian locomotor rhythm is normal in dnBMAL1-B mice. Actograms are double-plotted with each horizontal line representing 48 h. Mice were initially housed in a 12 h light:12 h dark (LD) light cycle then in constant darkness (DD). (Right panel) dnBMAL1-B mice show normal circadian period under DD condition. The data of WT mice are same with those used in Figure 2. All values are mean ± SEM. Individual data points are displayed as dots. * $p < 0.05$ as determined by two-way (G, H) or one-way (I-L) ANOVA with a post hoc test. The results of the statistical analyses are presented in Supplementary Table 2. Source data are provided as a source data file.

Supplementary Figure 3.

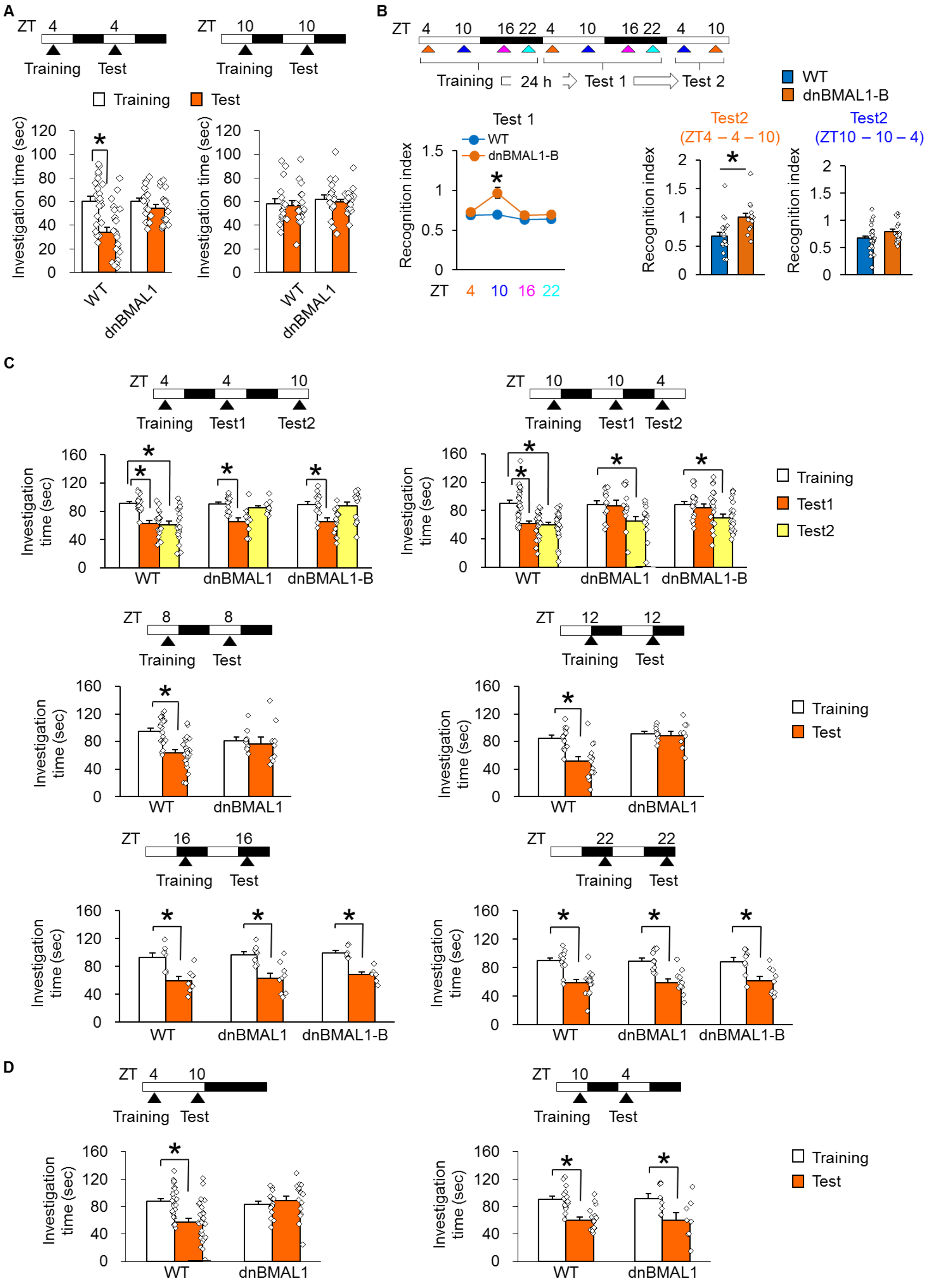

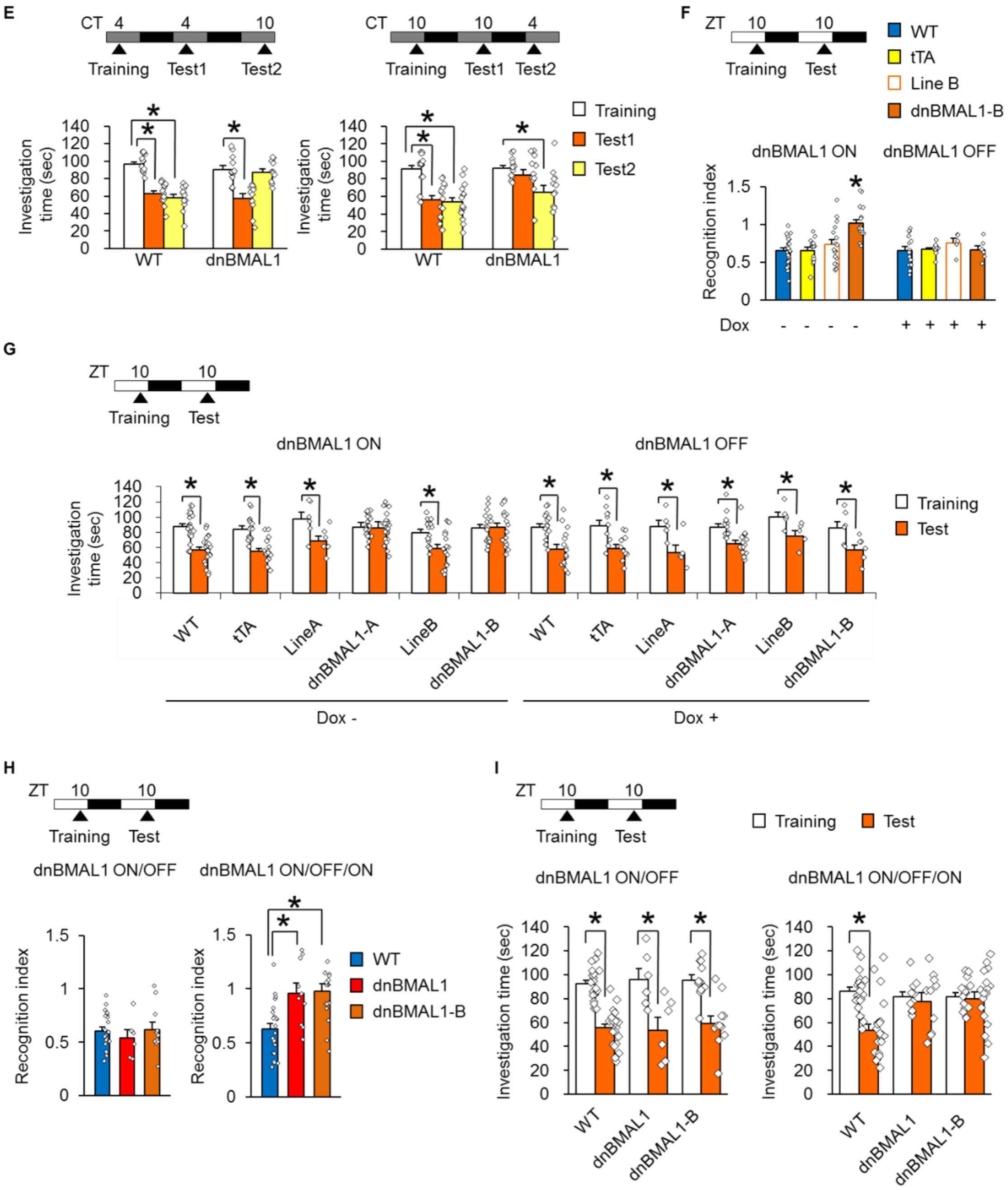
E
CT 4 4 10
Training Test1 Test2
Investigation time (sec)
140 120 100 80 60 40 20 0
WT dnBMAL1
CT 10 10 4
Training Test1 Test2
Training
Test1
Test2
F
ZT 10 10
Training Test
WT
tTA
Line B
dnBMAL1-B
dnBMAL1 ON
dnBMAL1 OFF
Recognition index
1.5 1 0.5 0
Dox - - - - + + + +
G
ZT 10 10
Training Test
dnBMAL1 ON
dnBMAL1 OFF
Investigation time (sec)
Training
Test
WT tTA LineA dnBMAL1-A LineB dnBMAL1-B WT tTA LineA dnBMAL1-A LineB dnBMAL1-B
Dox -
Dox +
H
ZT 10 10
Training Test
dnBMAL1 ON/OFF
dnBMAL1 ON/OFF/ON
Recognition index
WT
dnBMAL1
dnBMAL1-B
I
ZT 10 10
Training Test
Training
Test
dnBMAL1 ON/OFF
dnBMAL1 ON/OFF/ON
Investigation time (sec)
WT dnBMAL1 dnBMAL1-B

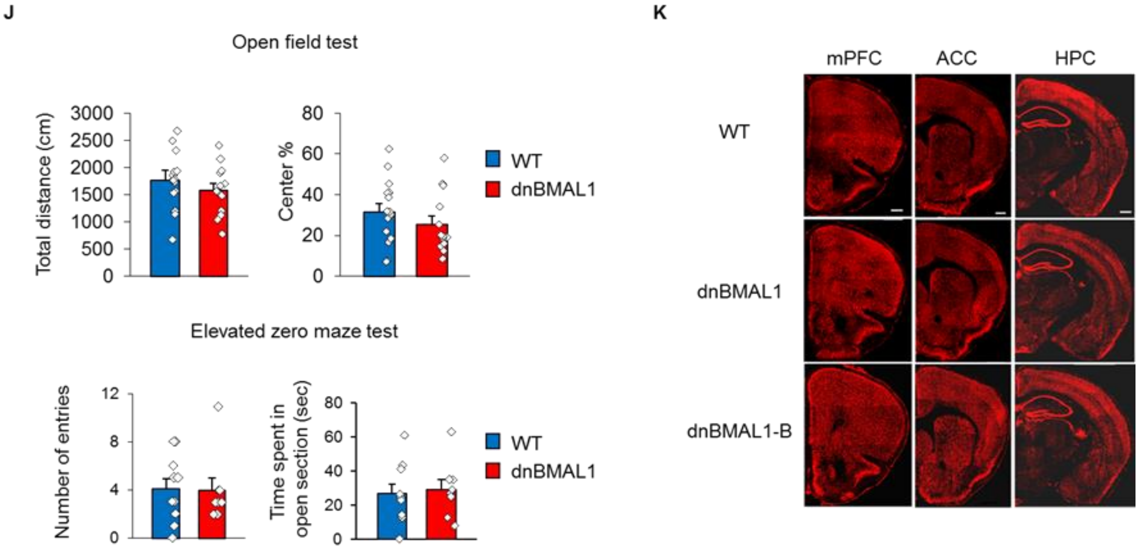

Supplementary Figure 3. dnBMAL1 expression-dependent deficits in retrieval of social recognition memory, related to Figure 3.

Experimental designs are illustrated at top of each panel.

(A) Investigation time in Figure 3A (weak training condition); Memory is impaired in dnBMAL1 mice at ZT4 and ZT10 under weak training condition. (Left panel) WT, but not dnBMAL1, mice significantly reduce investigation time at ZT4 ($p < 0.05$). (right panel) WT and dnBMAL1 mice fail to reduce investigation time at ZT10 ($p > 0.05$).

(B) Retrieval of social recognition memory is impaired in dnBMAL1-B mice at ZT10 under strong training condition (3 min exposure during training). Separate groups of WT and dnBMAL1-B mice are trained at ZT4, ZT10, ZT16 or ZT22 and tested 24 h later, respectively. (Left panel) Recognition index in Test1 compared to Training. dnBMAL1-B mice show impaired memory retrieval at ZT10. * $p < 0.05$, compared to WT mice tested at ZT10. (Right panels) Mice that are initially tested at ZT4 (Test1) are re-tested at ZT10 (Test2). Conversely, mice that are initially tested at ZT10 (Test1) are re-tested at ZT4 (Test2). Only dnBMAL1-B mice re-tested at ZT10 show retrieval deficit. The data of WT mice are same with those used in Figure 3B.

(C) Investigation time in Figure 3B and Figure S3B; Memory retrieval is impaired in dnBMAL1 and dnBMAL1-B mice at ZT10 under strong training condition. (Upper left panel) WT, dnBMAL1 and dnBMAL1-B mice significantly reduce investigation time in Test1 (ZT4, $p < 0.05$). dnBMAL1 and dnBMAL1-B, but not WT, mice fail to reduce investigation time in Test2 (ZT10, $p > 0.05$). (Upper right panel) dnBMAL1 and dnBMAL1-B, but not WT, mice fail to reduce investigation time in Test1 (ZT10, $p >$

0.05). WT, dnBMAL1 and dnBMAL1-B mice significantly reduce investigation time in Test2 (ZT4, $p < 0.05$). (Middle left and right panels) dnBMAL1, but not WT, mice fail to reduce investigation time at ZT8 (left) and ZT12 (right, $p > 0.05$). (Lower left and right panels) WT, dnBMAL1 and dnBMAL1-B mice significantly reduce investigation time at ZT16 (left) and ZT22 (right, $p < 0.05$).

(D) Investigation time in Figure 3C; Memory retrieval is impaired in dnBMAL1 mice at ZT10 under strong training condition. (Left panel) dnBMAL1, but not WT, mice fail to reduce in investigation time at ZT10 ($p > 0.05$). (Right panel) WT and dnBMAL1 mice significantly reduce investigation time at ZT4 ($p < 0.05$).

(E) Investigation time in Figure 3D; Under constant dark (DD), memory retrieval in dnBMAL1 mice is impaired at CT10. (Left panel) WT and dnBMAL1 mice significantly reduce investigation time in Test1 (CT4, $p < 0.05$). dnBMAL1, but not WT, mice fail to reduce investigation time in Test2 (CT10, $p > 0.05$). (Right panel) dnBMAL1, but not WT, mice, fail to reduce investigation time in Test1 (CT10, $p > 0.05$). WT and dnBMAL1 mice significantly reduce investigation time in Test2 (CT4, $p < 0.05$).

(F) Memory retrieval impairment depends on expression of dnBMAL1 [not observed in the presence of Dox in dnBMAL1-B mice, in single transgenic mice (tTA, Line B) or WT mice]. * $p < 0.05$, compared to the other control groups. The data of WT mice and tTA mice are same with those used in Figure 3E.

(G) Investigation time in Figure 3E and Figure S3F; Memory retrieval impairment depends on expression of dnBMAL1. dnBMAL1 and dnBMAL1-B ON mice, but not the other control groups, fail to reduce investigation time ($p > 0.05$).

(H, I) Memory retrieval impairment depends on expression of dnBMAL1. Separate groups of WT and dnBMAL1 mice are trained at ZT10 and tested 24 h later. (H) (Left panel) Normal memory retrieval is observed in dnBMAL1 ON/OFF mice. (Right panel) Memory retrieval is impaired (higher recognition index) in dnBMAL1 and dnBMAL1-B ON/OFF/ON mice. (I) Investigation time. dnBMAL1 and dnBMAL1-B ON/OFF/ON mice, but not the other control groups, fail to reduce investigation time ($p > 0.05$).

(J) dnBMAL1 mice show normal anxiety-related behavior in open field test (upper panel) and elevated zero maze test (lower panel). To further characterize dnBMAL1 mice, we examined anxiety-related behavior of dnBMAL1 mice. dnBMAL1 mice show comparable locomotor activity (total distance) and time spent in the center region of open field compared to WT mice. Similarly, these mutant mice show comparable number of entries into open section and time spent in the open section compared to WT

mice. Thus dnBMAL1 mice show normal anxiety-related behavior, suggesting that forebrain-expression of dnBMAL1 mice does not affect anxiety-related behaviors.

(K) No gross histological abnormalities are observed in forebrain of dnBMAL1 mice. Immunofluorescence analyses of brain of WT mice, dnBMAL1 and -B OFF/ON mice using antibody for NeuN. Scale bar, 400 µm. mPFC, medial prefrontal cortex; ACC, anterior cingulate cortex; HPC, hippocampus. All values are mean ± SEM. Individual data points are displayed as dots. * $p < 0.05$ as determined by two-way (B, F) or one-way (H, J) ANOVA with a post hoc test, paired t test (A, C-E, G, I). The results of the statistical analyses are presented in Supplementary Table 2. Source data are provided as a source data file.

Supplementary Figure 4.

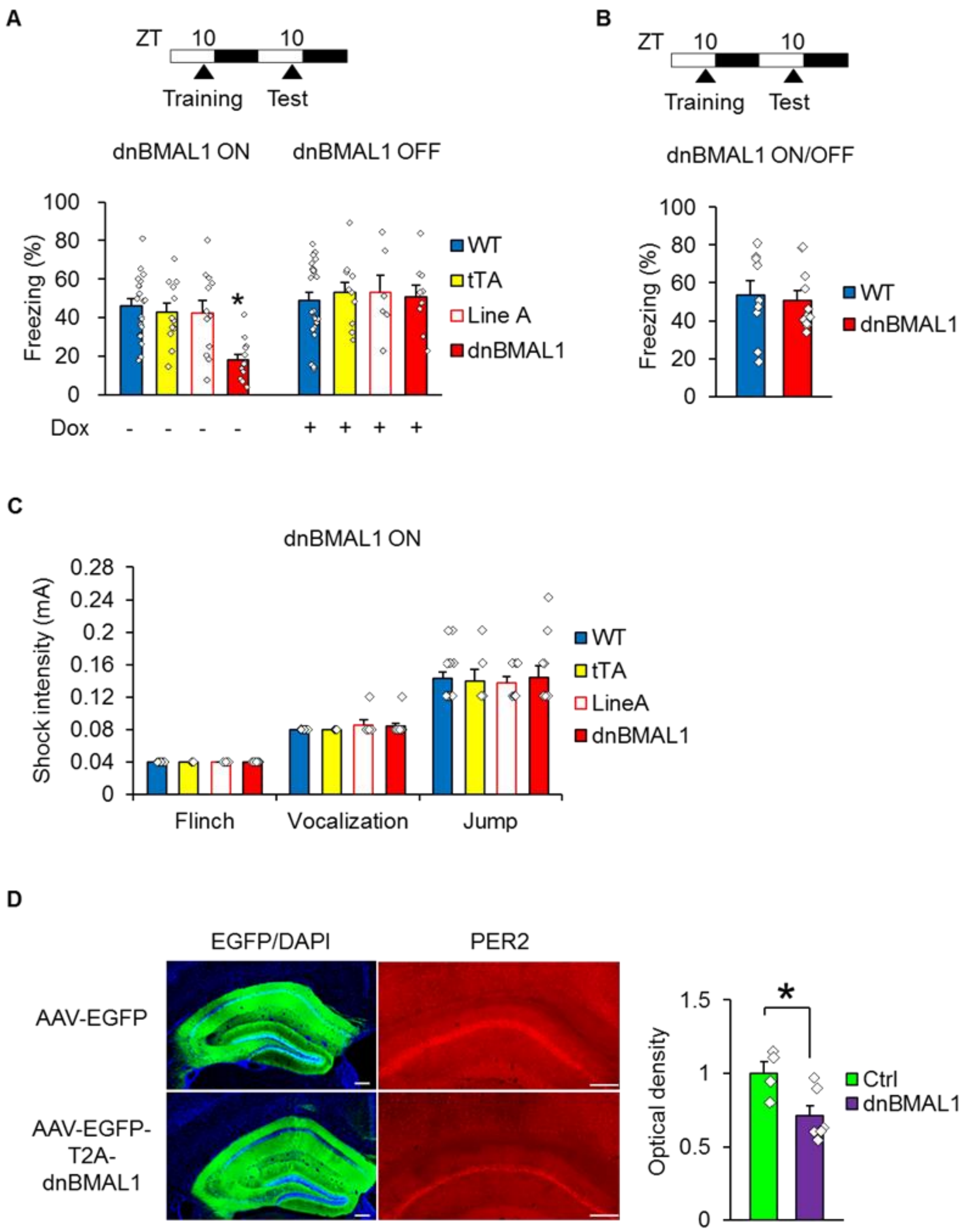

E

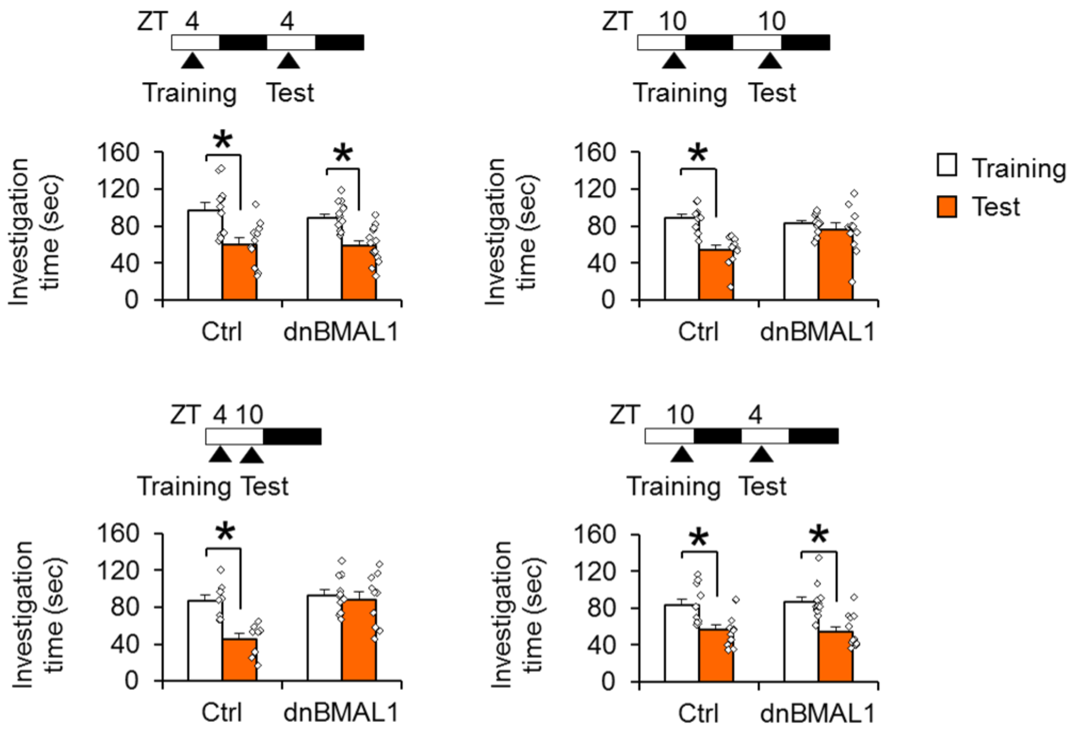
ZT 4 4
Training Test
ZT 10 10
Training Test
Training
Test
Investigation time (sec)
160
120
80
40
0
*
Ctrl
dnBMAL1
ZT 4 10
Training Test
ZT 10 4
Training Test

F

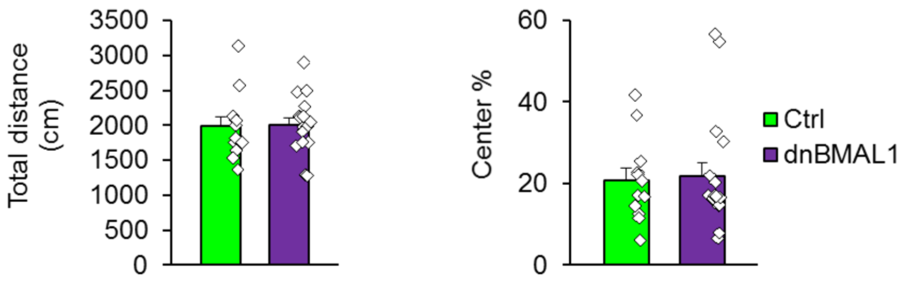
Total distance (cm)
3500
3000
2500
2000
1500
1000
500
0
Center %
60
40
20
0
Ctrl
dnBMAL1

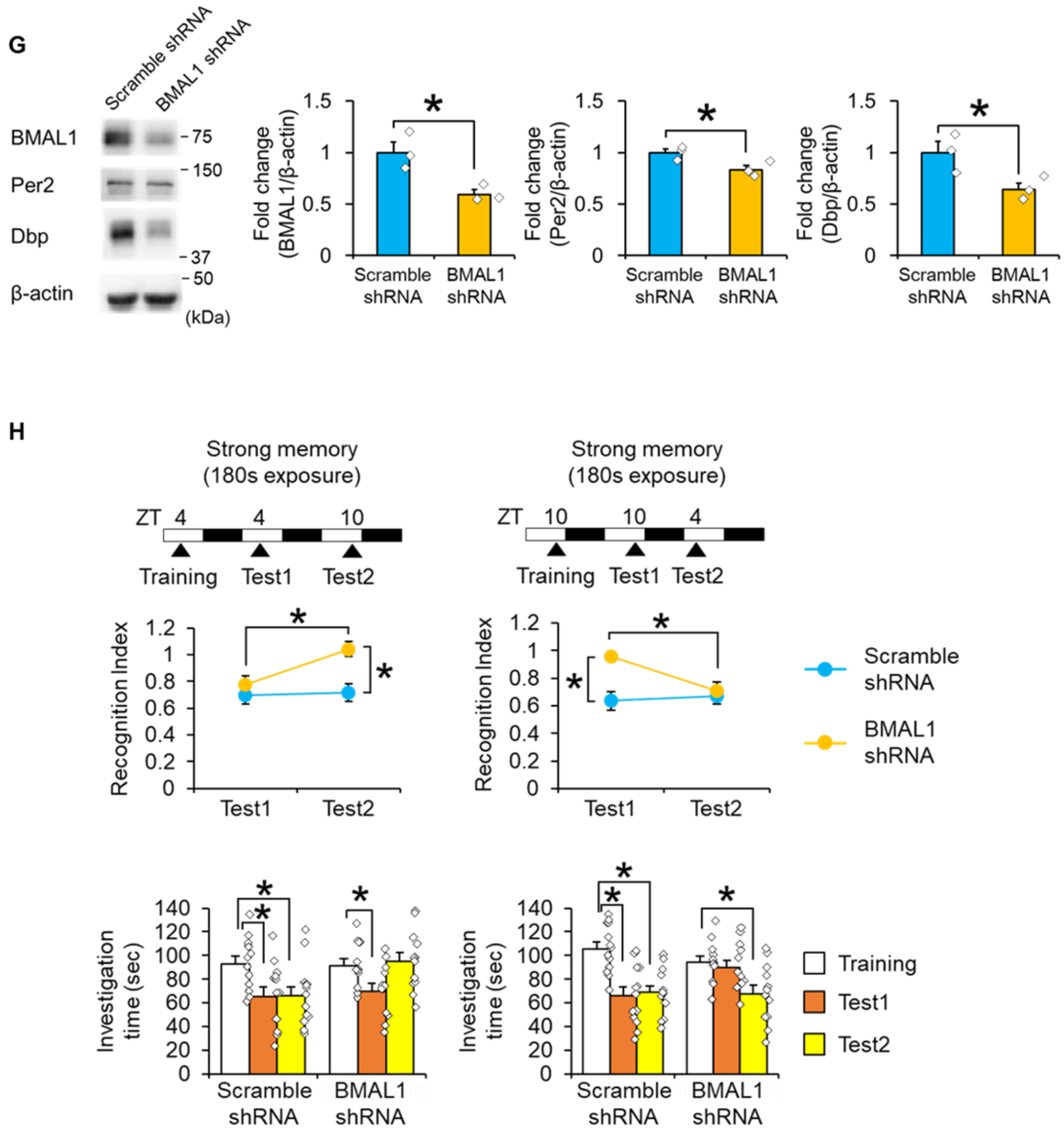

Supplementary Figure 4. dnBMAL1 expression-dependent deficits in retrieval of hippocampus-dependent memory, related to Figure 4.

(A, B, E and H) Experimental designs are illustrated at top of each panel.

(A) Memory retrieval impairment depends on expression of dnBMAL1 [not observed in the presence of Dox in dnBMAL1 mice, in single transgenic mice (tTA, Line A) or WT mice]. * $p < 0.05$, compared to the other control groups.

(B) Memory retrieval impairment is not observed in dnBMAL1 mice when Dox was

withdrawn.

(C) dnBMAL1 ON mice display normal pain sensitivity.

(D) (Left panels) Representative images of AAV-mediated EGFP expression (left, green, EGFP fluorescence; blue, nuclear stain using DAPI) and PER2 (right, immunohistochemistry using anti-PER2 antibody) expressions in the hippocampus of control (AAV-EGFP, upper) and dnBMAL1 (AAV-EGFP-T2A-dnBMAL1, lower) groups 4-5 weeks after AAV injection. Scale bar, 200 μm. (Right panel) Quantification of PER2 expression. One-way ANOVA with group reveal significant decreases of PER2 expression in CA1 region of hippocampus of dnBMAL1 group at ZT10 compared to control group. The graph represents fold changes compared to expression levels in Ctrl group.

(E) Investigation time in Figure 4D; AAV-mediated expression of dnBMAL1 in the hippocampus impairs memory retrieval at ZT10. (Upper left and lower right panel) Ctrl and dnBMAL1 groups significantly reduce investigation time at ZT4 ($p < 0.05$). (Upper right and lower left panel) dnBMAL1, but not Ctrl, group fails to reduce investigation time at ZT10 ($p > 0.05$).

(F) dnBMAL1 group show normal locomotor activity and anxiety-related behavior.

(G) (Left panel) Representative images of BMAL1, Per2 and Dbp expressions in the dorsal hippocampus of control (scramble shRNA) and shBMAL1 (BMAL1 shRNA) groups 4-5 weeks after AAV injection. Cortex region of shBMAL1 group was used as a negative control that does not induce knockdown of BMAL1. (Right panels) Quantification of BMAL1, Per2 and Dbp expressions. One-way ANOVA with group reveal significant decreases of BMAL1, Per2 and Dbp expressions in the dorsal hippocampus of shBMAL1 group at ZT10 compared to control group. The graph represents fold changes compared to expression levels in control group.

(H) (Upper panels) shBMAL1 expression in the hippocampus impairs retrieval of social recognition memory at ZT10, but not ZT4 (Recognition index). (Lower left panel) Control and shBMAL1 mice significantly reduce investigation time in Test1 (ZT4, $p < 0.05$). shBMAL1, but not control, mice fail to reduce investigation time in Test2 (ZT10, $p > 0.05$). (Lower right panel) shBMAL1, but not control, mice fail to reduce investigation time in Test1 (ZT10, $p > 0.05$). Control and shBMAL1 mice significantly reduce investigation time in Test2 (ZT4, $p < 0.05$). Ctrl, control. All values are mean ± SEM. Individual data points are displayed as dots. * $p < 0.05$ as determined by two-way (A, H) or one-way (B-D, F, G) ANOVA with a post hoc test, paired t test (E, H). The results of the statistical analyses are presented in Supplementary Table 2. Source data are provided as a source data file.

Supplementary Figure 5.

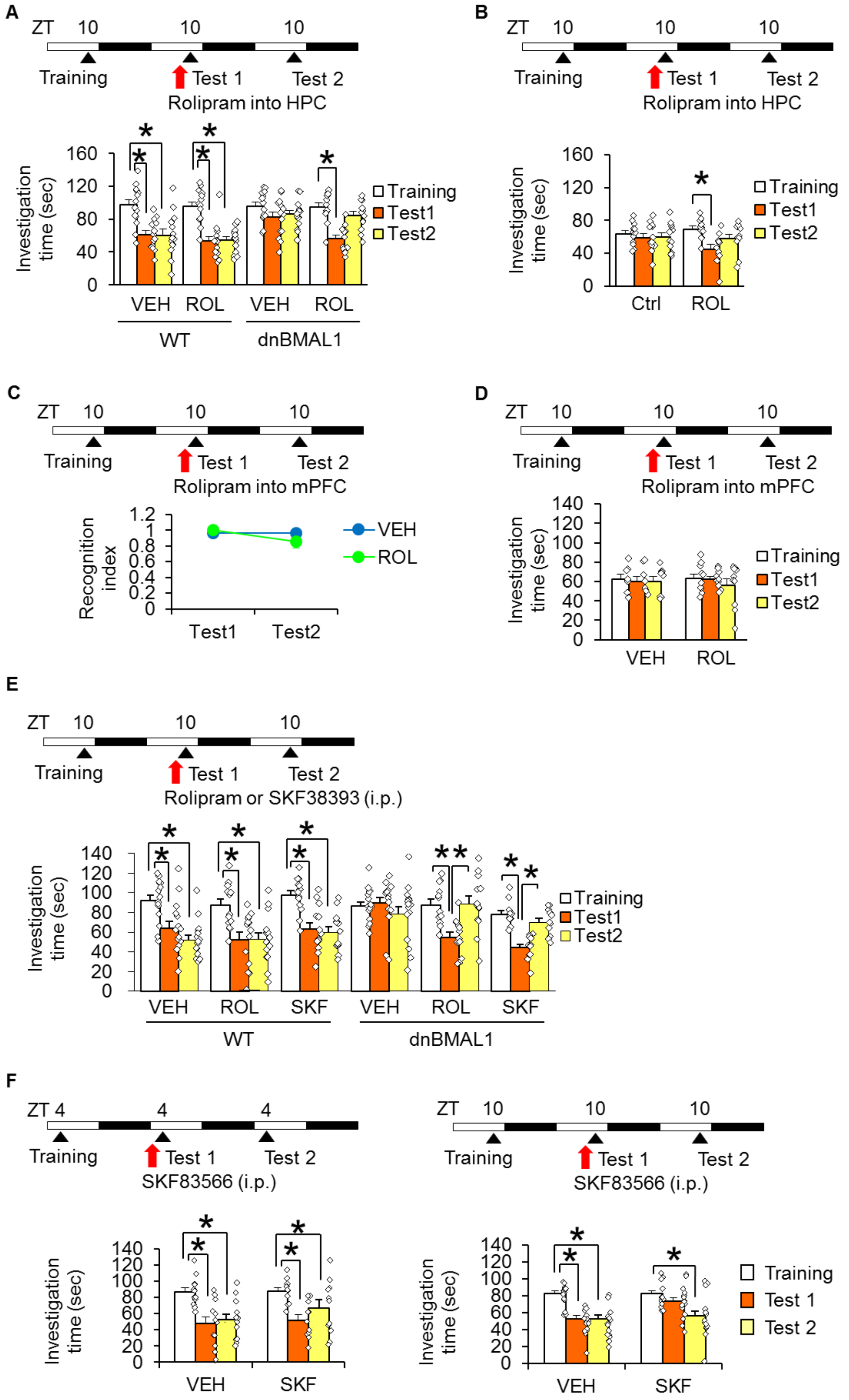

Supplementary Figure 5. Rolipram or D1/5R agonist (SKF38393) rescues retrieval deficit in dnBMAL1 and WT mice at ZT10 under strong or weak, respectively, training condition, related to Figure 5.

Experimental designs are illustrated at top of each panel.

(A) Investigation time in Figure 5E; Rolipram rescues retrieval deficit in dnBMAL1 mice. WT mice significantly reduce investigation time in Test1 and 2 compared to training in the presence and absence of micro-injection of rolipram ($p < 0.05$). dnBMAL1 mice significantly reduce investigation time in Test1 only in the presence of hippocampal-micro-infusion of rolipram ($p < 0.05$).

(B) Investigation time of Figure 5F; Rolipram rescues retrieval deficit in WT mice under weak training condition. Mice significantly reduce investigation time in Test1 only in the presence of micro-infusion of rolipram into the hippocampus (HPC) ($p < 0.05$).

(C) Micro-infusion of rolipram into mPFC, before Test1does not affect memory retrieval at Test1 (ZT10) in WT mice under weak training condition.

(D) Investigation time of Figure S5C. Micro-infusion of rolipram into mPFC does not affect memory retrieval in WT mice. Mice fail to reduce investigation time at Test1 even in the presence of rolipram infusion ($p > 0.05$).

(E) Investigation time of Figure 5G. Rolipram or SKF38393 rescue retrieval deficit in dnBMAL1 mice. WT mice significantly reduce investigation time in Test 1 and 2 compared to Training in the presence and absence of systemic injection of rolipram or SKF38393 ($p < 0.05$). dnBMAL1 mice significantly reduce investigation time in Test1 only in the presence of systemic injection of rolipram or SKF38393 ($p < 0.05$).

(F) Investigation time of Figure 5H. Systemic injection of low dose of SKF83566 impairs memory retrieval at ZT10, but not at ZT4, in WT mice under strong training condition. Mice fail to reduce investigation time in Test1 when tested at ZT10, only in the presence of SKF83566 ($p > 0.05$). VEH, vehicle; HPC, hippocampus; mPFC, medial prefrontal cortex. All values are mean ± SEM. Individual data points are displayed as dots. * $p < 0.05$ as determined by two-way (C), paired t test (A, B, D-F). The results of the statistical analyses are presented in Supplementary Table 2. Source data are provided as a source data file.

Supplementary Figure 6.

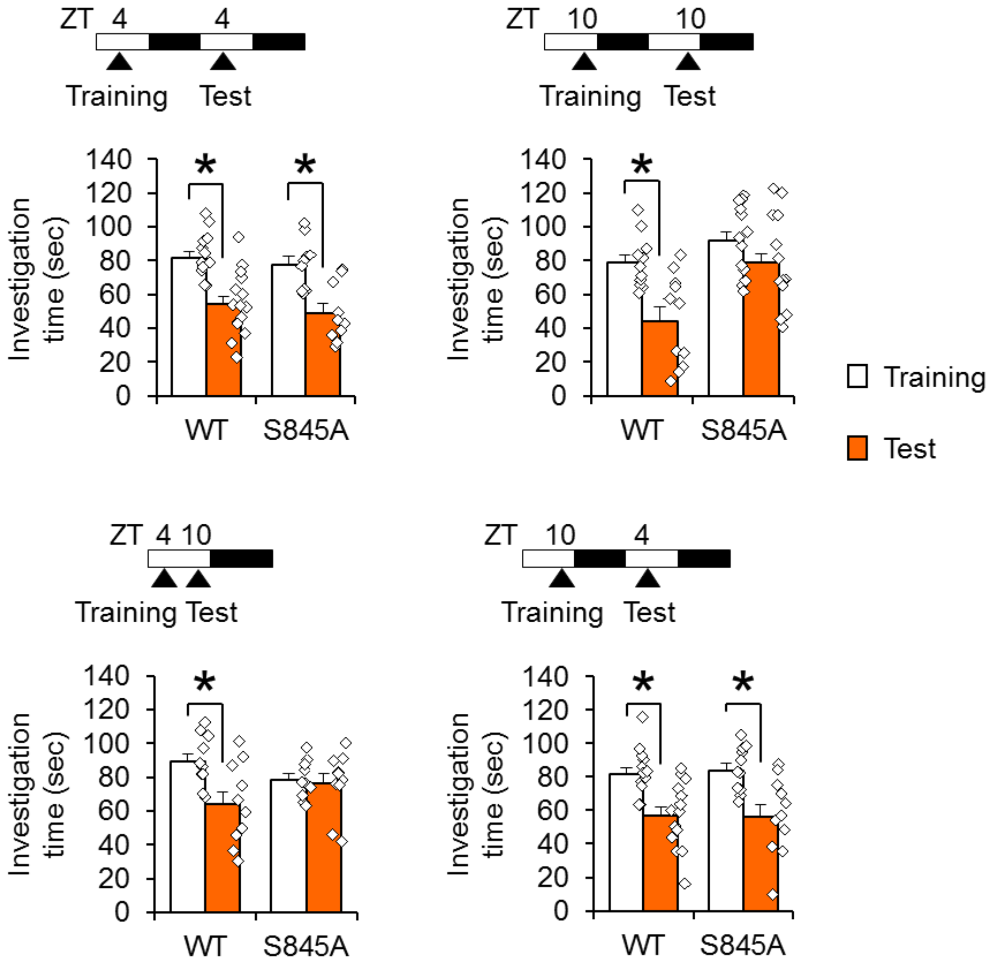

Supplementary Figure 6. Phosphorylation of GluA1 S845 is required for memory retrieval, related to Figure 6.

Experimental designs are illustrated at top of each panel. Investigation time in Figure 6B; S845A mice show retrieval deficit when tested at ZT10. (Upper left and lower right panels) WT and S845A mice significantly reduce investigation time when tested at ZT4 ($p < 0.05$). (Upper right and lower left panels) S845A, but not WT, mice fail to reduce investigation time when tested at ZT10 ($p > 0.05$). All values are mean ± SEM. Individual data points are displayed as dots. * $p < 0.05$ as determined by paired t test. The results of the statistical analyses are presented in Supplementary Table 2. Source data are provided as a source data file.

Supplementary Table 1.

List of all primer sequences used in genotyping, RT-PCR, qRT-PCR and ChIP assay.

Experiment	Genotype	Sequence (5' to 3')
Genotyping and RT-PCR	TRE-dnBMAL1	F: GCTGTCATCATGAGCCTCTTG
		R: CAGCAGTAGCCTCATCATC
	aCaMKII-tTA	F: CGCTGTGGGGCATTTTACTTTAG
		R: CATGTCCAGATCGAAATCGTC
	WT	F: CAAATGTTGCTTGTCTGGTG
		R: GTCAGTCGAGTGCACAGTTT
	GluA1 S845A	F: CCCAGGTCCTTGGTAATGATTGC
		R: AATGAGATAACACGGGGCTTGGTTCCTAAC

Experiment	Gene	Sequence (5' to 3')
qRT-PCR	*Bmal1*	F: GGCAACAGCTGCAGTATCAA
		R: TCCACAGCTAGCCCAAACTC
	Adcy1	F: TTGCTGGAGTGATCGGTGCT
		R: TCCTGCCGTGGGAACTGTTT
	Akap5	F: ACGATCTGGGTTGGGCTTCA
		R: TCCACTCTCCCCTCACACCA
	Drd1a	F: TCTCTTGGTGGCTGTCTTGG
		R: TAAGGATGGATGCCGTGGA
	Drd5	F: ATTTGTGGCTGGGAGGAGGG
		R: AGGCCCTTTGTTCTGCGAGT
	Per2	F: TGCGAGAGTGAGGAGAAAGG
		R: CTTCCGAGCACCGTCTAATG
	Dbp	F: AATGACCTTTGAACCTGATCCCGCT
		R: GCTCCAGTACTTCTCATCCTTCTGT
	GAPDH	F: ATGGCCTTCCGTGTTCCTAC
		R: GCCTGCTTCACCACCTTCTT

Experiment	Gene	Sequence (5' to 3')
ChIP qPCR	*Dbp* promoter [Transcription Start Site (TSS) -503 bp ~ -305 bp containing E-box sequence]	F: CTGTGAACACTCGGCTCCTT
		R: GTGTAGTTGCCTCGCCTTTT
	Clock exon 6	F: AGCTGGGGTCTATGCTTCCT
		R: CGCTGAGAGCCAAGACAAT

Supplementary Table 2.

Statistics and Sample Sizes.

Fig.	Method		Sample size	Statisitical Test	Comparison	Statatistics	P value
1B	Social recognition task	Recognition index	ZT4-4 (n = 27), ZT10-10 (n = 18), ZT4-10 (n = 10) and ZT10-4 (n = 45)	Two-way ANOVA	Factor 1: Training time	F(1, 96) = 0.70	>0.05
					Factor 2: Retrieval time	F(1, 96) = 15.81	<0.05
					Interaction (F1 x F2)	F(1, 96) = 1.04	>0.05
1C	qRT-PCR	BMAL1	n = 6 at each time point	One-way ANOVA		F(3, 20) = 23.75	<0.05
2B	Immunohistochemistry	PER2 (CA1)	All groups (n = 5)	Two-way ANOVA	Factor 1: Genotype	F(1, 16) = 18.21	<0.05
					Factor 2: Time	F(1, 16) = 2.82	>0.05
					Interaction (F1 x F2)	F(1, 16) = 0.85	>0.05
		DBP (CA1)	All groups (n = 5)	Two-way ANOVA	Factor 1: Genotype	F(1, 16) = 21.37	<0.05
					Factor 2: Time	F(1, 16) = 13.77	<0.05
					Interaction (F1 x F2)	F(1, 16) = 0.42	>0.05
2C	Immunohistochemistry	PER2 (SCN)	All groups (n = 5)	Two-way ANOVA	Factor 1: Genotype	F(1, 16) = 0.19	>0.05
					Factor 2: Time	F(1, 16) = 5.96	<0.05
					Interaction (F1 x F2)	F(1, 16) = 0.03	>0.05
		DBP (SCN)	All groups (n = 5)	Two-way ANOVA	Factor 1: Genotype	F(1, 16) = 0.04	>0.05
					Factor 2: Time	F(1, 16) = 39.76	<0.05
					Interaction (F1 x F2)	F(1, 16) = 0.05	>0.05

Fig.	Method		Sample size	Statisitical Test	Comparison	Statatistics	P value
2D	ChIP-qPCR	anti-CLOCK (Dbp promoter)	WT (n = 9), dnBMAL1 (n = 9)	One-way ANOVA		F(1, 16)= 5.97	<0.05
		anti-Rabbit IgG (Dbp promoter)	W (n = 8), dn (n = 8)			F(1, 14)= 0.5	>0.05
		anti-CLOCK (Clock exon6)	W (n = 8), dn (n = 8)			F(1, 14)= 0.36	>0.05
		anti-Rabbit IgG (Clock exon6)	W (n = 8), dn (n = 8)			F(1, 14)= 0.11	>0.05
2E	Quantitation of circadian rhythm parameters and activity levels	Circadian period	W (n = 8), dn (n = 8)	One-way ANOVA		F(1, 14) = 1.9	>0.05
		daily locomotor activity	W (n = 8), dn (n = 8)			F(1, 14) = 1.17	>0.05
3A	Social recognition	Recognition index	ZT4, W (n = 22), dn (n = 18); ZT10, W (n = 17), dn (n = 19)	Two-way ANOVA	Factor 1: Genotype	F(1, 72) = 4.69	<0.05
					Factor 2: Test time	F(1, 72) = 10.77	<0.05
					Interaction (F1 x F2)	F(1, 72) = 4.58	<0.05
3B	Social recognition task	Recognition index	ZT4, W (n = 19), dn (n = 12); ZT8, W (n = 21), dn (n = 9); ZT10, W (n = 28), dn (n = 13); ZT12, W (n = 14), dn (n = 9); ZT16, W (n = 8), dn (n = 9); ZT22, W (n = 14), dn (n = 11)	Two-way ANOVA	Factor 1: Genotype	F(1, 72) = 4.69	<0.05
					Factor 2: Test time	F(1, 72) = 10.77	<0.05
					Interaction (F1 x F2)	F(1, 72) = 4.58	<0.05
		Recognition index (ZT4-4-10)	W (n = 19), dn (n = 12)	Two-way repeated-measure ANOVA	Factor 1: Genotype	F(1, 29) = 4.90	<0.05
					Factor 2: Test time	F(1, 29) = 4.73	<0.05
					Interaction (F1 x F2)	F(1, 29) = 6.39,	<0.05
		Recognition index (ZT10-10-4)	W (n = 28), dn (n = 13)	Two-way repeated-measure ANOVA	Factor 1: Genotype	F(1, 39) = 7.78	<0.05
					Factor 2: Test time	F(1, 39) = 7.04	<0.05
					Interaction (F1 x F2)	F(1, 39) = 5.26	<0.05

Fig.	Method		Sample size	Statisitical Test	Comparison	Statatistics	P value
3C	Social recognition task	Recognition index (ZT4-10)	W (n = 27), dn (n = 15)	One-way ANOVA		F(1, 40) = 19.23	<0.05
		Recognition index (ZT10-4)	W (n = 16), dn (n = 8)			F(1, 22) = 0.13	>0.05
3D	Social recognition task	Recognition index (CT4-4-10)	W (n = 14), dn (n = 11)	Two-way repeated-measure ANOVA	Factor 1: Genotype	F(1, 23)= 12.53	<0.05
					Factor 2: Test time	F(1, 23)= 12.75	<0.05
					Interaction (F1 x F2)	F(1, 23)= 24.16	<0.05
		Recognition index (CT10-10-4)	W (n = 15), dn (n = 12)	Two-way repeated-measure ANOVA	Factor 1: Genotype	F(1, 25)= 7.73	<0.05
					Factor 2: Test time	F(1, 25)= 8.07	<0.05
					Interaction (F1 x F2)	F(1, 25)= 5.25	<0.05
3E	Social recognition task	Recognition index	Dox -, W (n = 24), tTA (n = 16), Line A (n = 6), dn (n = 26); Dox +, W (n = 15), tTA (n = 10), Line A (n = 5), dn (n = 13)	Two-way ANOVA	Factor 1: Genotype	F(3, 107) = 13.39	<0.05
					Factor 2: Dox	F(1, 107) = 5.43	<0.05
					Interaction (F1 x F2)	F(3, 107) = 3.96	<0.05
4A	Novel object recognition task	Discrimination index (ZT4-4-10)	W (n = 7), dn (n = 8)	Two-way repeated-measure ANOVA	Factor 1: Genotype	F(1, 13)= 7.19	<0.05
					Factor 2: Test time	F(1, 13)= 5.20	<0.05
					Interaction (F1 x F2)	F(1, 13)= 6.56	<0.05
		Discrimination index (ZT10-10-4)	W (n = 12), dn (n = 8)	Two-way repeated-measure ANOVA	Factor 1: Genotype	F(1, 18)= 5.29	<0.05
					Factor 2: Test time	F(1, 18)= 8.70	<0.05
					Interaction (F1 x F2)	F(1, 18)= 5.40	<0.05

Fig.	Method		Sample size	Statisitical Test	Comparison	Statatistics	P value
4B	Contextual fear conditioning task	Freezing (ZT4-4)	W (n = 16), dn (n = 9)	One-way ANOVA		F(1, 23)= 0.17	>0.05
		Freezing (ZT10-10)	W (n = 22), dn (n = 21)			F(1, 41)= 9.09	<0.05
		Freezing (ZT4-10)	W (n = 6), dn (n = 6)			F(1, 10)= 11.62	<0.05
		Freezing (ZT10-4)	W (n = 12), dn (n = 10)			F(1, 20)= 0.49	>0.05
4C	Immunohistochemistry	c-fos expression in CA1	All groups (n = 6)	Three-way ANOVA	Factor 1: Genotype	F(1, 40)= 6.27	<0.05
					Factor 2: Test time	F(1, 40)= 5.48	<0.05
					Factor 3: Retrieval	F(1, 40)= 55.23	<0.05
					Interaction (F1 x F2 x F3)	F(1, 40)= 4.74	<0.05
4D	Social recognition task	Recognition index (ZT4-4)	Ctrl (n = 11), dn (n = 14)	One-way ANOVA		F(1, 23)= 0.51	>0.05
		Recognition index (ZT10-10)	Ctrl (n = 11), dn (n = 12)			F(1, 21)= 10.87	<0.05
		Recognition index (ZT4-10)	Ctrl (n = 8), dn (n = 11)			F(1, 17)= 12.33	<0.05
		Recognition index (ZT10-4)	Ctrl (n = 11), dn (n = 12)			F(1, 21)= 0.2	>0.05
5C	qRT-PCR	AC1	All groups (n = 4)	One-way ANOVA		F(1, 6) = 37.71	<0.05
		AKAP5				F(1, 6) = 52.2	<0.05
		D1R				F(1, 6) = 16.8	<0.05
		D5R				F(1, 6) = 44.16	<0.05
5D	Cyclic AMP measurement	cAMP	W (n = 12), dn (n = 11)	One-way ANOVA		F(1, 21) = 6.62	<0.05

Fig.	Method		Sample size	Statisitical Test	Comparison	Statatistics	P value
5E	Social recognition task	Recognition index	WT-Vehicle (WT-VEH, n = 14), WT-rolipram (WT-ROL, n = 14), dnBMAL1-VEH (n = 14), dnBMAL1-ROL (n = 12)	Three-way repeated-measure ANOVA	Factor 1: Genotype	F(1, 50) = 20.42	<0.05
					Factor 2: Drug	F(1, 50) = 4.56	<0.05
					Factor 3: Test	F(1, 50) = 13.42	<0.05
					Interaction (F1 x F2 x F3)	F(1, 50) = 5.55	<0.05
5F	Social recognition task	Recognition index	Control (Ctrl, n = 12), ROL (n = 11)	Two-way repeated-measure ANOVA	Factor 1: Drug	F(1, 21) = 4.42	<0.05
					Factor 2: Test	F(1, 21) = 7.17	<0.05
					Interaction (F1 x F2)	F(1, 21) = 4.68	<0.05
5G	Social recognition task	Recognition index (Rolipram)	WT-VEH (n = 15), WT-ROL (n = 14), dnBMAL1-VEH (n = 16), dnBMAL1-ROL (n = 13)	Three-way repeated-measure ANOVA	Factor 1: Genotype	F(1, 54) = 24.83	<0.05
					Factor 2: Drug	F(1, 54) = 2.09	>0.05
					Factor 3: Test	F(1, 54) = 0.46	>0.05
					Interaction (F1 x F2 x F3)	F(1, 54) = 6.78	<0.05
		Recognition index (SKF38393)	WT-VEH (n = 15), WT-SKF38393 (WT-SKF, n = 13), dnBMAL1-VEH (n = 16), dnBMAL1-SKF (n = 11)	Three-way repeated-measure ANOVA	Factor 1: Genotype	F(1, 51) = 22.06	<0.05
					Factor 2: Drug	F(1, 51) = 4.88	<0.05
					Factor 3: Test	F(1, 51) = 0.13	>0.05
					Interaction (F1 x F2 x F3)	F(1, 51) = 6.42	<0.05

Fig.	Method		Sample size	Statisitical Test	Comparison	Statatistics	P value
5H	Social recognition task	Recognition index (ZT4-4-4)	Vehicle (VEH, n = 12), SKF83566 (SKF, n = 11)	Two-way repeated-mesure ANOVA	Factor 1: Drug	F(1, 21) = 0.91	>0.05
					Factor 2: Test	F(1, 21) = 5.38	<0.05
					Interaction (F1 x F2)	F(1, 21) = 1.32	>0.05
		Recognition index (ZT10-10-10)	VEH (n = 19), SKF (n = 21)	Two-way repeated-mesure ANOVA	Factor 1: Drug	F(1, 38) = 5.79	<0.05
					Factor 2: Test	F(1, 38) = 5.38	<0.05
					Interaction (F1 x F2)	F(1, 38) = 4.24	<0.05
6A	Western blotting	pS845/total GluA1	All groups (n = 8)	One-way ANOVA		F(1, 14) = 5.55	<0.05
		Total GluA1/β-actin	All groups (n = 8)			F(1, 14) = 1.06	>0.05
6B	Social recognition task	Recognition index (ZT4-4)	WT (W, n = 15), S845A (n = 10)	One-way ANOVA		F(1, 23)= 0.04	>0.05
		Recognition index (ZT10-10)	W (n = 11), S845A (n = 13)			F(1, 22)= 5.79	<0.05
		Recognition index (ZT4-10)	W (n = 10), S845A (n = 10)			F(1, 18)=6.97	<0.05
		Recognition index (ZT10-4)	W (n = 14), S845A (n = 11)			F(1, 23)= 0.003	>0.05
6C	Contextual fear conditioning task	Freezing (ZT4-4)	W (n = 9), S845A (n = 6)	One-way ANOVA		F(1, 13)= 0.22	>0.05
		Freezing (ZT10-10)	W (n = 16), S845A (n = 12)			F(1, 26)= 10.31	<0.05
		Freezing (ZT4-10)	W (n = 13), S845A (n = 13)			F(1, 24)= 5.97	<0.05
		Freezing (ZT10-4)	W (n = 11), S845A (n = 13)			F(1, 22)= 1.85	>0.05

Fig.	Method		Sample size	Statisitical Test	Comparison	Statatistics	P value
S1A	Social recognition task	Recognition index	PBS (n = 16), lidocaine (n = 18)	One-way ANOVA		F(1, 32) = 19.5	<0.05
S1B	Social recognition task	Investigation time (PBS)	PBS (n = 16)	paired t test			<0.05
		Investigation time (Lidocaine)	lidocaine (n = 18)	paired t test			>0.05
S1C	Social recognition task	Recognition index	ZT4-4 (n = 14), ZT10-10 (n = 18), ZT4-10 (n = 29), ZT10-4 (n = 19)	Two-way ANOVA	Factor 1: Training time	F(1, 76) = 0.004	>0.05
					Factor 2: Retrieval time	F(1, 76) = 0.694	>0.05
					Interaction (F1 x F2)	F(1, 76) = 0.045	>0.05
S1D	Social recognition task	Investigation time (ZT4-4)	ZT4-4 (n = 14)	paired t test			<0.05
		Investigation time (ZT10-10)	ZT10-10 (n = 18)				<0.05
		Investigation time (ZT4-10)	ZT4-10 (n = 29)				<0.05
		Investigation time (ZT10-4)	ZT10-4 (n = 19)				<0.05
S1E	Social recognition task	Investigation time (ZT4-4)	ZT4-4 (n = 27)	paired t test			<0.05
		Investigation time (ZT10-10)	ZT10-10 (n = 18)				>0.05
		Investigation time (ZT4-10)	ZT4-10 (n = 10)				>0.05
		Investigation time (ZT10-4)	ZT10-4 (n = 45)				<0.05

Fig.	Method		Sample size	Statisitical Test	Comparison	Statatistics	P value
S2G	Immunohistochemistry	PER2 (CA1)	All groups (n = 5)	Two-way ANOVA	Factor 1: Genotype	F(1, 16) = 29.42	<0.05
					Factor 2: Time	F(1, 16) = 4.30	>0.05
					Interaction (F1 x F2)	F(1, 16) = 0.67	>0.05
		DBP (CA1)	All groups (n = 5)		Factor 1: Genotype	F(1, 16) = 31.36	<0.05
					Factor 2: Time	F(1, 16) = 10.15	<0.05
					Interaction (F1 x F2)	F(1, 16) = 1.82	>0.05
S2H	Immunohistochemistry	PER2 (SCN)	All groups (n = 5)	Two-way ANOVA	Factor 1: Genotype	F(1, 16) = 0.05	>0.05
					Factor 2: Time	F(1, 16) = 6.78	<0.05
					Interaction (F1 x F2)	F(1, 16) = 0.01	>0.05
		DBP (SCN)	All groups (n = 5)		Factor 1: Genotype	F(1, 16) = 0.33	>0.05
					Factor 2: Time	F(1, 16) = 43.75	<0.05
					Interaction (F1 x F2)	F(1, 16) = 0.51	>0.05
S2I	qRT-PCR	Per2	WT (n = 4), dnBMAL1 (n = 4)	One-way ANOVA		F(1, 6) = 14.63	<0.05
		Dbp	WT (n = 4), dnBMAL1 (n = 4)			F(1, 6) = 9.538	<0.05

Fig.	Method		Sample size	Statisitical Test	Comparison	Statatistics	P value
S2J	Western blotting	Per2	WT (n = 4), dnBMAL1 (n = 4)	One-way ANOVA		F(1, 6) = 6.029	<0.05
		Dbp	WT (n = 6), dnBMAL1 (n = 6)			F(1, 10) = 10.107	<0.05
S2K	ChIP-qPCR	anti-CLOCK (Dbp promoter)	WT (W, n = 9), dnBMAL1-B (dn-B, n = 9)	One-way ANOVA		F(1, 16)= 8.28	<0.05
		anti-Rabbit IgG (Dbp promoter)	W (n = 8), dn-B (n = 8)			F(1, 14)= 1.96	>0.05
		anti-CLOCK (Clock exon6)				F(1, 14)= 0.15	>0.05
		anti-Rabbit IgG (Clock exon6)				F(1, 14)= 0.35	>0.05
S2L	Quantitation of circadian rhythm parameters and activity levels	Circadian period	W (n = 8), dn-B (n = 8)	One-way ANOVA		F(1, 14) = 3.41	>0.05
		daily locomotor activity	W (n = 8), dn-B (n = 8)	One-way ANOVA		F(1, 14) = 7.4	<0.05
S3A	Social recognition task	Investigation time (WT: ZT4-4)	W (n = 22)	paired t test			<0.05
		Investigation time (dnBMAL: ZT4-4)	dn (n = 18)				>0.05
		Investigation time (WT: ZT10-10)	W (n = 17)				>0.05
		Investigation time (dnBMAL: ZT10-10)	dn (n = 19)				>0.05

Fig.	Method		Sample size	Statisitical Test	Comparison	Statatistics	P value
S3B	Social recognition task	Recognition index (ZT4, 10, 16, 22)	ZT4, W (n = 19), dn-B (n = 14); ZT10, W (n = 28), dn-B (n = 20); ZT16, W (n = 8), dn-B (n = 7); ZT22, W (n = 14), dn-B (n = 9)	Two-way ANOVA	Factor 1: Genotype	F(1, 111) = 7.34	<0.05
					Factor 2: Test time	F(3, 111) = 5.70	<0.05
					Interaction (F1 x F2)	F(3, 111) = 3.45	<0.05
		Recognition index (ZT4-4-10)	W (n = 19), dn-B (n = 14)	Two-way repeated-measure ANOVA	Factor 1: Genotype	F(1, 31) = 7.26	<0.05
					Factor 2: Test time	F(1, 31) = 6.50	<0.05
					Interaction (F1 x F2)	F(1, 31) = 8.28	<0.05
		Recognition index (ZT10-10-4)	W (n = 28), dn-B (n = 20)	Two-way repeated-measure ANOVA	Factor 1: Genotype	F(1, 46) = 13.49	<0.05
					Factor 2: Test time	F(1, 46) = 6.70	<0.05
					Interaction (F1 x F2)	F(1, 46) = 4.35	<0.05

Fig.	Method		Sample size	Statisitical Test	Comparison	Statatistics	P value
S3C	Social recognition task	Investigation time (WT: ZT4-4-10: Test1)	W (n = 19)	paired t test			<0.05
		Investigation time (WT: ZT4-4-10: Test2)					<0.05
		Investigation time (dnBMAL1: ZT4-4-10: Test1)	dn (n = 12)				<0.05
		Investigation time (dnBMAL1: ZT4-4-10: Test2)					>0.05
		Investigation time (dnBMAL1-B: ZT4-4-10: Test1)	dn-B (n = 14)				<0.05
		Investigation time (dnBMAL1-B: ZT4-4-10: Test2)					>0.05
		Investigation time (WT: ZT10-10-4: Test1)	W (n = 28)				<0.05
		Investigation time (WT: ZT10-10-4: Test2)					<0.05
		Investigation time (dnBMAL1: ZT10-10-4: Test1)	dn (n = 13)				>0.05
		Investigation time (dnBMAL1: ZT10-10-4: Test2)					<0.05
		Investigation time (dnBMAL1-B: ZT10-10-4: Test1)	dn-B (n = 20)				>0.05
		Investigation time (dnBMAL1-B: ZT10-10-4: Test2)					<0.05
		Investigation time (WT: ZT8-8)	W (n = 21)				<0.05
		Investigation time (dnBMAL1: ZT8-8)	dn (n = 9)				>0.05
		Investigation time (WT: ZT12-12)	W (n = 14)				<0.05
		Investigation time (dnBMAL1: ZT12-12)	dn (n = 9)				>0.05
		Investigation time (WT: ZT16-16)	W (n = 8)				<0.05
		Investigation time (dnBMAL1: ZT16-16)	dn (n = 9)				<0.05
		Investigation time (dnBMAL1-B: ZT16-16)	dn-B (n = 7)				<0.05
		Investigation time (WT: ZT22-22)	W (n = 14)				<0.05
		Investigation time (dnBMAL1: ZT22-22)	dn (n = 11)				<0.05
		Investigation time (dnBMAL1-B: ZT22-22)	dn-B (n = 9)				<0.05

Fig.	Method		Sample size	Statisitical Test	Comparison	Statatistics	P value
S3D	Social recognition task	Investigation time (WT: ZT4-10)	W (n = 27)	paired t test			<0.05
		Investigation time (dnBMAL1: ZT4-10)	dn (n = 15)				>0.05
		Investigation time (WT: ZT10-4)	W (n = 16)				<0.05
		Investigation time (dnBMAL1: ZT10-4)	dn (n = 8)				<0.05
S3E	Social recognition task	Investigation time (WT: CT4-4-10: Test1)	W (n = 14)	paired t test			<0.05
		Investigation time (WT: CT4-4-10: Test2)					<0.05
		Investigation time (dnBMAL1: CT4-4-10: Test1)	dn (n = 11)				<0.05
		Investigation time (dnBMAL1: CT4-4-10: Test2)					>0.05
		Investigation time (WT: CT10-10-4: Test1)	W (n = 15)				<0.05
		Investigation time (WT: CT10-10-4: Test2)					<0.05
		Investigation time (dnBMAL1: CT10-10-4: Test1)	dn (n = 12)				>0.05
		Investigation time (dnBMAL1: CT10-10-4: Test2)					<0.05
S3F	Social recognition task	Recognition index	Dox -, W (n = 24), tTA (n = 16), Line B (n = 17), dn-B (n = 17); Dox +, W (n = 15), tTA (n = 10), Line B (n = 5), dn-B (n = 6)	Two-way ANOVA	Factor 1: Genotype	F(3, 102) = 4.49	<0.05
					Factor 2: Dox	F(1, 102) = 3.94	<0.05
					Interaction (F1 x F2)	F(3, 102)= 4.30	<0.05

Fig.	Method		Sample size	Statisitical Test	Comparison	Statatistics	P value
S3G	Social recognition task	Investigation time (dnBMAL1 ON: WT)	W (n = 24)	paired t test			<0.05
		Investigation time (dnBMAL1 ON: tTA)	tTA (n = 16)				<0.05
		Investigation time (dnBMAL1 ON: LineA)	Line A (n = 6)				<0.05
		Investigation time (dnBMAL1 ON: dnBMAL1-A)	dn (n = 26)				>0.05
		Investigation time (dnBMAL1 ON: LineB)	Line B (n = 17)				<0.05
		Investigation time (dnBMAL1 ON: dnBMAL1-B)	dn-B (n = 17)				>0.05
		Investigation time (dnBMAL1 OFF: WT)	W (n = 15)				<0.05
		Investigation time (dnBMAL1 OFF: tTA)	tTA (n = 10)				<0.05
		Investigation time (dnBMAL1 OFF: LineA)	Line A (n = 5)				<0.05
		Investigation time (dnBMAL1 OFF: dnBMAL1-A)	dn (n = 13)				<0.05
		Investigation time (dnBMAL1 OFF: LineB)	Line B (n = 5)				<0.05
		Investigation time (dnBMAL1 OFF: dnBMAL1-B)	dn-B (n = 6)				<0.05
S3H	Social recognition task	Recognition index (dnBMAL1 ON/OFF):	W (n = 22), dn (n = 6), dn-B (n = 11)	One-way ANOVA		F(2, 36) = 0.39	>0.05
		Recognition index (dnBMAL1 ON/OFF/ON)	W (n = 22), dn (n = 10), dn-B (n = 17)			F(2, 46) = 9.60	<0.05

Fig.	Method		Sample size	Statisitical Test	Comparison	Statatistics	P value
S3I	Social recognition task	Investigation time (dnBMAL1 ON/OFF: WT)	W (n = 22)	paired t test			<0.05
		Investigation time (dnBMAL1 ON/OFF: dnBMAL1)	dn (n = 6)				<0.05
		Investigation time (dnBMAL1 ON/OFF: dnBMAL-B)	dn-B (n = 11)				<0.05
		Investigation time (dnBMAL1 ON/OFF/ON: WT)	W (n = 22)				<0.05
		Investigation time (dnBMAL1 ON/OFF/ON: dnBMAL1)	dn (n = 10)				>0.05
		Investigation time (dnBMAL1 ON/OFF/ON: dnBMAL-B)	dn-B (n = 17)				>0.05
S3J	Open field test	Total distance	W (n = 15), dn (n = 13)	One-way ANOVA		F(1, 26) = 0.94	>0.05
		Center %	W (n = 15), dn (n = 13)			F(1, 26) = 1.07	>0.05
	Elevated zero maze test	Number of entries	W (n = 10), dn (n = 8)			F(1, 16) = 0.01	>0.05
		Time spent in Open section	W (n = 10), dn (n = 8)			F(1, 16) = 0.08	>0.05
S4A	Contextual fear conditioning task	Freezing	Dox -, W (n = 20), tTA (n = 12), Line A (n = 12), dn (n = 12); Dox +, W (n = 23), tTA (n = 11), Line A (n = 6), dn (n = 9)	Two-way ANOVA	Factor 1: Genotype	F(3, 97) = 2.97	<0.05
					Factor 2: Dox	F(1, 97) = 13.88	<0.05
					Interaction (F1 x F2)	F(3, 97)= 3.16	<0.05
S4B	Contextual fear conditioning task	Freezing	W (n = 9), dn (n = 11)	One-way ANOVA		F(1, 18) = 0.11	>0.05

Fig.	Method		Sample size	Statisitical Test	Comparison	Statatistics	P value
S4C	Pain sensitivity	Vocalization	W (n = 19), tTA (n = 6), Line A (n = 7), dn (n = 10)	One-way ANOVA		F(3, 38) = 1.06	>0.05
		Jump				F(3, 38) = 0.07	>0.05
S4D	Immunohistochemistry	PER2	Ctrl (n = 4), dn (n = 6)	One-way ANOVA		F(1, 8)= 6.84	<0.05
S4E	Social recognition task	Investigation time (ZT4-4: Ctrl)	Ctrl (n = 11)	paired t test			<0.05
		Investigation time (ZT4-4: dnBMAL1)	dn (n = 14)				<0.05
		Investigation time (ZT10-10: Ctrl)	Ctrl (n = 11)				<0.05
		Investigation time (ZT10-10: dnBMAL1)	dn (n = 12)				>0.05
		Investigation time (ZT4-10: Ctrl)	Ctrl (n = 8)				<0.05
		Investigation time (ZT4-10: dnBMAL1)	dn (n = 11)				>0.05
		Investigation time (ZT10-4: Ctrl)	Ctrl (n = 11)				<0.05
		Investigation time (ZT10-4: dnBMAL1)	dn (n = 12)				<0.05
S4F	Open field test	Total distance	Ctrl (n = 12), dn (n = 18)	One-way ANOVA		F(1, 28) = 0.03	>0.05
		Center %				F(1, 28) = 0.06	>0.05

Fig.	Method		Sample size	Statisitical Test	Comparison	Statatistics	P value
S4G	Western blotting	BMAL1	Scramble (n=3), shBMAL1 (n=3)	One-way		F(1, 4) = 13.552	<0.05
		PER2	Scramble (n=3), shBMAL1 (n=3)	One-way		F(1, 4) = 9.194	<0.05
		DBP	Scramble (n=3), shBMAL1 (n=3)	One-way		F(1, 4) = 8.477	<0.05
S4H	Social recognition task	Recognition index (ZT4-4-10)	Scramble (n=13), shBMAL1 (n=12)	Two-way repeated-measure ANOVA	Factor 1: Genotype	F(1, 23) = 7.114	<0.05
					Factor 2: Test time	F(1, 23) = 11.106	<0.05
					Interaction (F1 x F2)	F(1, 23) = 7.858	<0.05
		Recognition index (ZT10-10-4)	Scramble (n=13), shBMAL1 (n=12)	Two-way repeated-measure ANOVA	Factor 1: Genotype	F(1, 23) = 7.051	<0.05
					Factor 2: Test time	F(1, 23) = 6.643	<0.05
					Interaction (F1 x F2)	F(1, 23) = 11.199	<0.05
		Investigation time (Scramble: ZT4-4-10: Test1)	Scramble (n=13)	paired t test			<0.05
		Investigation time (Scramble: ZT4-4-10: Test2)					<0.05
		Investigation time (shBMAL1: ZT4-4-10: Test1)	shBMAL1 (n=12)				<0.05
		Investigation time (shBMAL1: ZT4-4-10: Test2)					>0.05
		Investigation time (Scramble: ZT10-10-4: Test1)	Scramble (n=13)	paired t test			<0.05
		Investigation time (Scramble: ZT10-10-4: Test2)					<0.05
		Investigation time (shBMAL1: ZT10-10-4: Test1)	shBMAL1 (n=12)				>0.05
		Investigation time (shBMAL1: ZT10-10-4: Test2)					<0.05

Fig.	Method		Sample size	Statisitical Test	Comparison	Statatistics	P value
S5A	Social recognition task	Investigation time (WT: VEH: Test1)	WT-VEH (n = 14)	paired t test			<0.05
		Investigation time (WT: VEH: Test2)					<0.05
		Investigation time (WT: ROL: Test1)	WT-ROL (n = 14)				<0.05
		Investigation time (WT: ROL: Test2)					<0.05
		Investigation time (dnBMAL1: VEH: Test1)	dnBMAL1-VEH (n = 14)				>0.05
		Investigation time (dnBMAL1: VEH: Test2)					>0.05
		Investigation time (dnBMAL1: ROL: Test1)	dnBMAL1-ROL (n = 12)				<0.05
		Investigation time (dnBMAL1: ROL: Test2)					>0.05
S5B	Social recognition task	Investigation time (VEH: Test1)	Ctrl (n = 12)	paired t test			>0.05
		Investigation time (VEH: Test2)					>0.05
		Investigation time (ROL: Test1)	ROL (n = 11)				<0.05
		Investigation time (ROL: Test2)					>0.05
S5C	Social recognition task	Recognition index	VEH (n = 8), ROL (n = 10)	Two-way repeated-measure ANOVA	Factor 1: Drug	$F(1, 16) = 0.33$	>0.05
					Factor 2: Test	$F(1, 16) = 1.16$	>0.05
					Interaction (F1 x F2)	$F(1, 16) = 0.88$	>0.05

Fig.	Method		Sample size	Statisitical Test	Comparison	Statatistics	P value
S5D	Social recognition task	Investigation time (VEH: Test1)	VEH (n = 8)	paired t test			>0.05
		Investigation time (VEH: Test2)					>0.05
		Investigation time (ROL: Test1)	ROL (n = 10)				>0.05
		Investigation time (ROL: Test2)					>0.05
S5E	Social recognition task	Investigation time (WT: VEH: Test1)	WT-VEH (n = 15)	paired t test			<0.05
		Investigation time (WT: VEH: Test2)					<0.05
		Investigation time (WT: ROL: Test1)	WT-ROL (n = 14)				<0.05
		Investigation time (WT: ROL: Test2)					<0.05
		Investigation time (WT: SKF: Test1)	WT-SKF38393 (n = 13)				<0.05
		Investigation time (WT: SKF: Test2)					<0.05
		Investigation time (dnBMAL1: VEH: Test1)	dnBMAL1-VEH (n = 16)				>0.05
		Investigation time (dnBMAL1: VEH: Test2)					>0.05
		Investigation time (dnBMAL1: ROL: Test1)	dnBMAL1-ROL (n = 13)				<0.05
		Investigation time (dnBMAL1: ROL: Test2)					>0.05
		Investigation time (dnBMAL1: SKF: Test1)	dnBMAL1-SKF38393 (n = 11)				<0.05
		Investigation time (dnBMAL1: SKF: Test2)					>0.05

Fig.	Method		Sample size	Statisitical Test	Comparison	Statatistics	P value
S5F	Social recognition task	Investigation time (ZT4-4-4: VEH: Test1)	VEH (n = 12)	paired t test			<0.05
		Investigation time (ZT4-4-4: VEH: Test2)					<0.05
		Investigation time (ZT4-4-4: SKF: Test1)	SKF83566 (n = 11)				<0.05
		Investigation time (ZT4-4-4: SKF: Test2)					<0.05
		Investigation time (ZT10-10-10: VEH: Test1)	VEH (n = 19)				<0.05
		Investigation time (ZT10-10-10: VEH: Test2)					<0.05
		Investigation time (ZT10-10-10: SKF: Test1)	SKF83566 (n = 21)				>0.05
		Investigation time (ZT10-10-10: SKF: Test2)					<0.05
S6A	Social recognition task	Investigation time (ZT4-4: WT)	WT (W, n = 15)	paired t test			<0.05
		Investigation time (ZT4-4: S845A)	S845A (n = 10)				<0.05
		Investigation time (ZT10-10: WT)	W (n = 11)				<0.05
		Investigation time (ZT10-10: S845A)	S845A (n = 13)				>0.05
		Investigation time (ZT4-10: WT)	W (n = 10)				<0.05
		Investigation time (ZT4-10: S845A)	S845A (n = 10)				>0.05
		Investigation time (ZT10-4: WT)	W (n = 14)				<0.05
		Investigation time (ZT10-4: S845A)	S845A (n = 11)				<0.05
