# Supplementary material for: Hippocampal clock regulates memory retrieval via Dopamine and PKA-induced GluA1 phosphorylation
Source: Nat Commun. 2019 Dec 18;10:5766. doi: 10.1038/s41467-019-13554-y (PMC6920429; doi:10.1038/s41467-019-13554-y)
Supplement: Supplementary file 3 — Reporting Summary [file 41467_2019_13554_MOESM3_ESM.pdf]

nature research

Corresponding author(s): Satoshi Kida

Last updated by author(s): Nov 7, 2019

Reporting Summary

Nature Research wishes to improve the reproducibility of the work that we publish. This form provides structure for consistency and transparency in reporting. For further information on Nature Research policies, see Authors & Referees and the Editorial Policy Checklist .

Statistics

For all statistical analyses, confirm that the following items are present in the figure legend, table legend, main text, or Methods section.

n/a	Confirmed	
☐	☒	The exact sample size (n) for each experimental group/condition, given as a discrete number and unit of measurement
☐	☒	A statement on whether measurements were taken from distinct samples or whether the same sample was measured repeatedly
☐	☒	The statistical test(s) used AND whether they are one- or two-sided<br>*Only common tests should be described solely by name; describe more complex techniques in the Methods section.*
☒	☐	A description of all covariates tested
☐	☒	A description of any assumptions or corrections, such as tests of normality and adjustment for multiple comparisons
☐	☒	A full description of the statistical parameters including central tendency (e.g. means) or other basic estimates (e.g. regression coefficient) AND variation (e.g. standard deviation) or associated estimates of uncertainty (e.g. confidence intervals)
☐	☒	For null hypothesis testing, the test statistic (e.g. F, t, r) with confidence intervals, effect sizes, degrees of freedom and P value noted<br>*Give P values as exact values whenever suitable.*
☒	☐	For Bayesian analysis, information on the choice of priors and Markov chain Monte Carlo settings
☒	☐	For hierarchical and complex designs, identification of the appropriate level for tests and full reporting of outcomes
☒	☐	Estimates of effect sizes (e.g. Cohen's d, Pearson's r), indicating how they were calculated

Our web collection on statistics for biologists contains articles on many of the points above.

Software and code

Policy information about availability of computer code

Data collection	Gene set collections C2 (curated gene sets: 4731 gene sets) and C5 (GO gene sets: 5917 gene sets) were obtained from Molecular Signature Database (MSigDB) v6.0 [http://software.broadinstitute.org/gsea/msigdb/collections.jsp].
Data analysis	For RNA-sequencing, the following softwares were used:<br>Filtering, mapping, and differential expression analysis were performed using CLC Genomics Workbench software ver. 9.5 (Qiagen).<br>Predictive causal analysis was performed using Ingenuity Pathway Analysis software (IPA, Ingenuity, Redwood City, CA, USA).<br>Gene set enrichment analysis (GSEA) was performed using GSEA ver. 2.0.3.<br><br>Statistical analyses were performed using StatView version 5.0 (SAS Institute Inc., Cary, NC, USA).

For manuscripts utilizing custom algorithms or software that are central to the research but not yet described in published literature, software must be made available to editors/reviewers. We strongly encourage code deposition in a community repository (e.g. GitHub). See the Nature Research guidelines for submitting code & software for further information.

Data

Policy information about availability of data

All manuscripts must include a data availability statement. This statement should provide the following information, where applicable:

- Accession codes, unique identifiers, or web links for publicly available datasets
- A list of figures that have associated raw data
- A description of any restrictions on data availability

The data supporting the findings of this study are available from the corresponding author upon reasonable request.

Field-specific reporting

Please select the one below that is the best fit for your research. If you are not sure, read the appropriate sections before making your selection.

- [x] Life sciences
- [ ] Behavioural & social sciences
- [ ] Ecological, evolutionary & environmental sciences

For a reference copy of the document with all sections, see nature.com/documents/nr-reporting-summary-flat.pdf

Life sciences study design

All studies must disclose on these points even when the disclosure is negative.

Sample size	Sample sizes were based on previous our studies that use similar methods.
Data exclusions	No data were excluded from the analyses.
Replication	All results were successfully confirmed at least twice.
Randomization	Same number of male and female mice with similar age were used.
Blinding	All experiments were performed blind.

Reporting for specific materials, systems and methods

We require information from authors about some types of materials, experimental systems and methods used in many studies. Here, indicate whether each material, system or method listed is relevant to your study. If you are not sure if a list item applies to your research, read the appropriate section before selecting a response.

Materials & experimental systems

n/a	Involved in the study	
[ ]	[x]	Antibodies
[x]	[ ]	Eukaryotic cell lines
[x]	[ ]	Palaeontology
[ ]	[x]	Animals and other organisms
[x]	[ ]	Human research participants
[x]	[ ]	Clinical data

Methods

n/a	Involved in the study	
[x]	[ ]	ChIP-seq
[x]	[ ]	Flow cytometry
[x]	[ ]	MRI-based neuroimaging

Antibodies

Antibodies used

For western blotting, the following antibodies and dilutions were used:
Primary antibody; rabbit polyclonal anti-GluA1 antibody (AB1504, 1:1000; Merck Millipore), anti-GluA1 phospho-Ser845 antibody (AB5849, 1:1000; Merck Millipore), anti-BMAL1 antibody (NB100-2288, 1:1000; Novus Biologicals, Littleton, CO, USA), anti-Per2 antibody (PER21-A, 1:1000; Alpha Diagnostic International, San Antonio, TX, USA), anti-Dbp antibody (PM079, 1:1000; MBL), or mouse monoclonal anti-β-Actin antibody (A1978, 1:10000; Sigma-Aldrich).
Secondary antibody; goat anti-rabbit IgG HRP (sc-2004, Santa Cruz), goat anti-mouse IgG HRP (sc-2005, Santa Cruz).

For ChIP assay, the following antibodies were used:
anti-CLOCK antibody (ab3517, Abcam, Cambridge, UK) or anti-rabbit IgG (ab46540, Abcam).

For immunohistochemistry, the following antibodies and dilutions were used:
Primary antibody; rabbit polyclonal anti-c-fos (ABE457, 1:1000; Merck Millipore), rabbit polyclonal anti-PER2 (PER21-A, 1:1000; Alpha Diagnostic International, San Antonio, TX, USA), rabbit polyclonal anti-DBP (LS-B3479, 1:1000; Lifespan Bioscience, Seattle, WA, USA) or mouse anti-NeuN (MAB377, 1:500; Merck Millipore).
Secondary antibody; HRP-conjugated secondary antibody (711-036-152, 1:500; Jackson Immunoresearch Laboratories, West Grove, PA, USA) for c-fos and NeuN, biotinylated secondary antibody (711-066-152, 1:500; Jackson Immunoresearch Laboratories) for PER2 and DBP (Fig. 2, Supplementary√ Fig. 2), or goat anti-rabbit IgG secondary antibody, Alexa Fluor® 555 conjugate (A-21428, 1:500; Thermo Fisher Scientific) for PER2 (Supplementary√ Fig. 4D), Alexa-Fluor568 conjugated streptavidin (S11226, Invitrogen, Eugene, OR, USA).

Validation

Antibodies were validated on the used species by using a dilution series of the primary and secondary antibodies based on manufacturers recommendations.

Animals and other organisms

Policy information about studies involving animals; ARRIVE guidelines recommended for reporting animal research

Laboratory animals	C57BL/6N (Charles River, Yokohama, Japan) and GluA1 S845A knockin (Jackson Laboratory, stock number 012613) mice were used.
Wild animals	N/A
Field-collected samples	N/A
Ethics oversight	All of the experiments were conducted according to the Guide for the Care and Use of Laboratory Animals, Japan Neuroscience Society and Tokyo University of Agriculture. All the animal experiments were approved by the Animal Care and Use Committee of Tokyo University of Agriculture (authorization number: 280036).

Note that full information on the approval of the study protocol must also be provided in the manuscript.
