# Supplementary material for: Hippocampal clock regulates memory retrieval via Dopamine and PKA-induced GluA1 phosphorylation
Source: Nat Commun. 2019 Dec 18;10:5766. doi: 10.1038/s41467-019-13554-y (PMC6920429; doi:10.1038/s41467-019-13554-y)
Supplement: Supplementary file 2 — Peer Review [file 41467_2019_13554_MOESM2_ESM.pdf]

Reviewers' Comments:

Reviewer #1:
Remarks to the Author:
In “Hippocampal clock regulates memory retrieval via Dopamine and PKA-induced GluA1 phosphorylation”, Hasegawa et al. provide evidence that the circadian transcription factor BMAL1 may be important for memory retrieval. Using inducible expression of a dominant negative-acting BMAL1 in the forebrain, the authors demonstrate a cAMP-dependent inhibition of memory retrieval that is downstream of dopamine receptor signaling. This is a very captivating and understudied area of neuroscience and the attempts to bridge this gap are both interesting and important. Several major issues exist, however, which dampen enthusiasm for the publication of this article in Nature Communications.
Major comments:
1. The test used for figures 1-2 (weaker social recognition task) should be repeated during the active phase. Since the encoding is similar at ZT10 and ZT4, it is unlikely that this task is heavily dependent on sleep-dependent consolidation immediately after the task, as the ZT10 trained animals would soon after be in their active phase. It is difficult to say whether memory formation and retention of this task would be similar during the active phase, but this should be tested, especially considering previous reports indicating that cAMP in the hippocampus is under circadian control.
2. Figure 2B shows “optical density” measurements for PER2 and DBP. These circadian antibodies are notoriously poor and considering the low amplitude expression of Dbp in the hippocampus (compared to some peripheral tissues), both the message and the protein should be considered and the western results shown, not just the measurements of optical density from the western blots.
3. (lines 124-125): There is not sufficient evidence to state that endogenous BMAL1 is reduced at ZT10. The message does appear to be lower (Figure 1C), however, in some peripheral tissues, Bmal1 message is actually anti-phase to the protein’s transcriptional activity and the peak of the chromatin occupancy. Since protein levels and chromatin recruitment aren’t tested throughout the circadian cycle in this region, the conclusion that the deficit at ZT10 corresponds to “low Bmal1” is not appropriate. BMAL1 ChIP-seq should be performed to verify whether there is low BMAL1 recruitment to E boxes at this zeitgeber time.
4. While the restoration of retrieval by increasing cAMP signaling is convincing, the links between RNA-seq data and BMAL1 are very confusing. (This is in part due to the issue brought up in comment #3.) BMAL1 has dual roles- transcriptional and translational- and it is not clear based on the mouse model in which capacity the dominant negative BMAL1 is exerting its effects. cAMP has already been shown to be important for various aspects of memory processing (consolidation, retention, and retrieval), and thus the conceptual advance that this paper is missing is precisely how dnBMAL1 is affecting this pathway. This could be addressed by looking at BMAL1 in different subcellular fractions and at a minimum, by performing ChIP-seq or ChIP-qPCR experiments at different circadian times.

Reviewer #2:
Remarks to the Author:
Various aspects of cognitition including memory consolidation depend on the circadian cycle. For example, memory retrieval declines in the late afternoon-early evening and maintenance of long-term memory depends on the circadian cycle. However, the role of local brain circadian clocks in memory remains unclear. In this paper they convincingly demonstrate that the hippocampal clock regulated by BMAL1 regulates time-of-day retrieval. Inducible transgenic dominant negative BMAL1 (dnBMAL1) expression in the mouse forebrain or hippocampus compromised retrieval of hippocampal memories at Zeitgeber Time 8-12, independent of retention delay, encoding time or Zeitgeber entrainment cue. This altered retrieval profile was apparently due to down regulation of hippocampus dopamine-cAMP

signaling in dnBMAL1 mice. This decreased dopamine receptors and GluA1-S845 phosphorylation by PKA. Pharmacological activation of cAMP-signals or D1/5Rs rescued impaired retrieval efficiency in dnBMAL1 mice. GluA1 S845A knock-in mice showed retrieval deficits with dnBMAL1 mice. These data suggest mechanisms underlying regulation of retrieval efficiency by hippocampal clock through the D1/5R-cAMP-PKA- GluA1 pathway.
This an interesting and novel study suitable for publication in Nature Communications. It provides a new molecular mechanism that explains time-of-time dependence for memory retrieval. The major conclusions are justified by the data, the paper is clearly written and the data is technically solid. I recommend that the paper be accepted for publication.
45

Reviewer #3:
Remarks to the Author:
This manuscript describes clock mechanisms regulating circadian effects on memory retrieval. The authors claim mechanisms underlying time of day effects on cognitive performance and memory are unknown (e.g., see in introduction, line 60: "Although it has been known since experiments by Ebbinghuas (1885) that time-of-day influences cognitive performance and memory…the mechanisms underlying this effect are unknown"). However, circadian and time-of-day effects of learning and memory are known to exist across animal phyla, and there is extensive evidence for core-clock machinery in regulating this process, including the brain regions they describe; their claims should better reflect the current state of the field (see below). Here in this report, the authors focus on the molecular pathways involving the transcription factor BMAL1 and Dopamine-cAMP pathways regulating hippocampus-dependent memory. The authors primarily use a hippocampus-dependent social recognition task where adult mice are exposed to juvenile mice, and the amount of exploration time of the adult mouse during a follow-up test trial with the same juvenile is indexed as memory. Varying the amount of initial exposure time to the juvenile generates a corresponding variance in recognition memory. The authors first describe time-of-day effects of weaker (2min) training that exists when trained and tested at ZT4 (24hr memory) but is blocked when trained and tested at ZT10. They then trained at ZT4 and tested at ZT10 and show impairment, which was not observed when animals were trained at ZT10 and tested at ZT4. The authors have adequate controls showing that stronger training (3min) abolishes this time-of-day effect. The authors then show diurnal changes in hippocampal BMAL1 RNA- although the methods are unclear (see below).
The authors then describe 2 spatial- and temporally-controlled dominant-negative (dn) BMAL mouse models, where they can induce dnBMAL1 in forebrain regions using a CAMKii promoter and the tet (tTA) on-off system. In this model, the removal of Dox treatment induces the dnBMAL1 in hippocampus (and other forebrain regions). These mice show reduced expression of downstream BMAL1 target genes, and normal expression in the SCN region and normal circadian locomotor activity (good controls for brain target specificity). The authors then use this model to test whether time-of-day dependent social-recognition retrieval is affected by dnBMAL in forebrain regions and show deficits at ZT4. Line 131 reads "deficits at both ZT4 and ZT10…" however, figures 3A and S3A show a dnBMAL deficit only at ZT4, with no differences between WT and dnBMAL1 at ZT10; therefore, this statement needs to be corrected (see below). The authors then use a strong training protocol to test dnBMAL mice, and survey many points over the day to show 24hr retrieval differences only at ZT8, ZT10, and ZT12 compared to WT. The authors then report differences in retrieval with training at ZT4 and testing at ZT10 with the strong training protocol. They did not observe differences in these protocols under DD (constant dark) conditions, suggesting they are independent of external light cues. The authors provide supplemental data that describes the dnBMAL effects on social retrieval only when the transgene is turned ON (Figure S3) but the results, legend, and methods all fail to describe adequately the timing of Dox treatment (ON/OFF vs ON/OFF/ON, etc.) which needs to be addressed (see below).

The authors also test dnBMAL mice in object recognition and contextual fear conditioning and show the time-of-day dependent effects of dnBMAL are generalizable to different forms of memory. The data presented are confusing to what is described in the results (see below). The authors then performed RNA-seq on dnBMAL1 mice and report the identification reductions in RNA for adenylate cyclase and dopamine receptors (D1R and D5R), along with reductions in cAMP levels, compared to WT mice. They go on to show blocking hippocampal cAMP degradation (PDE4) activity with microinfusion of rolipram rescues the dnBMAL effects following strong training at ZT10, as well as retrieval following weak training in WT mice at ZT10. IP injection of a D1/5R agonist rescued dnBMAL1 retrieval deficits at ZT10, with no effects on WT, but another D1/5R agonist impaired WT retrieval at ZT10 but not ZT4. This last observation is challenging to understand and deserves attention, either experimentally, or minimally in the discussion (see below).
Lastly, the authors rationalize that D1/5R activation phosphorylates AMPA receptor GluA1 via PKA, so they examined activity of the phosphorylation site on GluA1 (S845) in the synaptosomal fractions of hippocampus at ZT10 in dnBMAL mice compared to WT mice, and found a reduction in dnBMAL mice. The authors then used GluA1 S845A knock-in mice to test the effects on time-of-day dependent social recognition and contextual fear conditioning. They observed impairments at ZT10 testing but not ZT4 in S845A knock-in mice compared to WT; mimicking the effects of dnBMAL mice.

Minor concerns:

1) The authors make the claim that the role of mechanisms in time-of-day memory/cognitive performance is unknown. This is not an accurate reflection of the current state of the field, and several statements of the manuscript should be rephrased to better represent this. For example, in the introduction, in line 63 should be edited from "…this effect are unknown" to read "…this effect are not well understood" – similarly, in line 258 of the discussion, the authors state that "…no study has examined this directly" while citing Shimizu et al., 2016 (Nature Comm.) in reference to circadian transcriptional clocks in the forebrain regulating circadian memory processes. Shimizu et al., (Nature Comm. 2016) tested the effects of BMAL conditional knock-out mice on circadian gene expression and long-term NOR memory. These mice have BMAL expression preserved in the SCN, but not in the hippocampus and other brain regions, which is important since this determined whether circadian expression of core clock genes in the SCN or forebrain/hippocampal circuits conferred the circadian effects on long-term NOR memory. Shimizu et al. showed that BMAL cKO expression blocks circadian regulation of genes in the hippocampus, and blocks circadian changes in NOR memory, which already described circadian regulation of clock genes in the hippocampus are required for circadian regulation of NOR long-term memory. Shimizu et al. also show it is the time of training, and not the time of testing, that appears to affect this temporal difference in long-term memory formation. This contrasts with what the current authors observed here in the case of their dnBMAL1 model, where instead it is the time of retrieval. The authors should dedicate space within the discussion to address this discrepancy with previous studies.

2) The authors need to better describe the differences in hippocampal changes in BMAL expression (Fig. 1C)- which technique was used, qPCR or FISH? While the methods describe both techniques, it is not clear which is used in Figure 1C, as it is not mentioned in the results, legend, or RNA analysis section of the methods. How was the hippocampus isolated for RNA work? What region(s) – CA1, CA3, Dentate gyrus, or all?

3) The authors need to describe the conditions (timing/etc.) for what dnBMAL ON/OFF and ON/OFF/ON mean in terms of training/testing in social recognition task (Fig S3). This should be clearly laid out minimally in methods.

4) In Figure4A (right panel), the X-axis looks to be reversed for Testing at ZT10 versus ZT4; the current X-axis shows ZT4 has differences and ZT10 does not- but the results section explains ZT10 has the differences (lines 170-173).

5) Figure 5G shows the effects of D1/5R agonist SKF38393 does not influence strong social recognition test in WT mice at ZT10, however, Figure 5H shows the effects of D1/5R agonist SKF83566 on social recognition retrieval following strong training in WT mice at ZT10. At ZT4, there does not appear to be any effect of SKF83566, however, there is a significant deficit at ZT10 in WT mice. In the discussion, line 282-285 reads "Importantly, pharmacologically restoring cAMP levels or D1/5R activities rescued retrieval deficits of strong and weak memory observed in dnBMAL1 mice and WT mice, respectively, at ZT10." This statement is not supported by these data. There are multiple issues. Please parse out these observations and handle them in the discussion separately. For example, rephrase the statement in line 282-285 to something like "Importantly, pharmacologically restoring cAMP levels or D1/5R activities rescued retrieval deficits of strong memory observed in dnBMAL1 mice at ZT10" and then return to the issues with SKFs and WT mice: "However, D1/5R activity in WT mice was able to impair strong recognition at ZT10 with SKF83566, but not with SKF38393. " <then explain why you think this is>. Also, why was this second agonist performed in WT and not dnBMAL mice? The authors may choose perform additional experiments on SKF83566 on dnBMAL mice with strong training to complement the WT studies, or omit the WT studies with SKF83566 all together. The better experiment testing D1/5R agonist (SKF38393) was already performed with dnBMAL and WT at ZT10 following strong training (Figure 5G).

6) It is unclear why the authors use the term "efficiency" to describe the circadian effects retrieval memory. What exactly does this mean? How is the audience to interpret this term? The term is never defined, remains ambiguous, and should be removed from the manuscript. It detracts from the substantial enthusiasm of this report. Discarding this "efficiency" term from the manuscript will avoid confusion.

7) It would greatly help the reader if the authors label in the Figures "Strong" or "Weak" where applicable.

Reviewers' comments:

Reviewer #1 (Remarks to the Author):

In “Hippocampal clock regulates memory retrieval via Dopamine and PKA-induced GluA1 phosphorylation”, Hasegawa et al. provide evidence that the circadian transcription factor BMAL1 may be important for memory retrieval. Using inducible expression of a dominant negative-acting BMAL1 in the forebrain, the authors demonstrate a cAMP-dependent inhibition of memory retrieval that is downstream of dopamine receptor signaling. This is a very captivating and understudied area of neuroscience and the attempts to bridge this gap are both interesting and important. Several major issues exist, however, which dampen enthusiasm for the publication of this article in Nature Communications.

Major comments:

Point 1)

The test used for figures 1-2 (weaker social recognition task) should be repeated during the active phase. Since the encoding is similar at ZT10 and ZT4, it is unlikely that this task is heavily dependent on sleep-dependent consolidation immediately after the task, as the ZT10 trained animals would soon after be in their active phase. It is difficult to say whether memory formation and retention of this task would be similar during the active phase, but this should be tested, especially considering previous reports indicating that cAMP in the hippocampus is under circadian control.

Response) Thank you for your important comment. We performed additional experiments during the active phase (**Additional data 1** as shown below; data not shown in the manuscript). WT and dnBMAL1 mice were trained and tested during active phase (ZT16-16, ZT22-22) in weaker training condition of social recognition task. Similar with the results at ZT4-ZT4 and ZT10-ZT10, dnBMAL1 mice showed retrieval deficits of weak social recognition memory at ZT16-ZT16 and ZT22-ZT22 although WT mice show normal retrieval of this memory at both these time points. These results support our conclusion that dnBMAL1 mice show lower efficiency of memory retrieval compared to WT mice.

Additional data 1

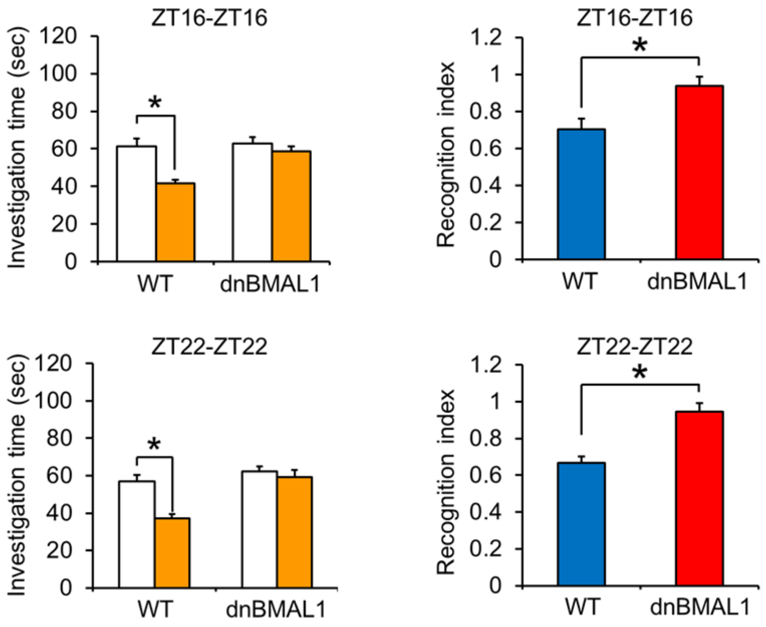

WT_ZT16, n=14; dnBMAL1_ZT16, n=20; WT_ZT22, n=12; dnBMAL1_ZT22, n=19.
Mean ± SEM. Asterisk represents significant difference at $P < 0.05$.

Point 2)
Figure 2B shows "optical density" measurements for PER2 and DBP. These circadian antibodies are notoriously poor and considering the low amplitude expression of Dbp in the hippocampus (compared to some peripheral tissues), both the message and the protein should be considered and the western results shown, not just the measurements of optical density from the western blots.

Response) Thank you for your important comment. We performed quantitative RT-PCR experiment and western blotting. dnBMAL1 mice showed significantly reduced Per2 and Dbp mRNA levels in the hippocampus at ZT10 (**Additional data 2** as shown below**; Figure S2I**). Our results of western blotting showed that expression of dnBMAL1 significantly reduced expression of Per2 and Dbp proteins in the hippocampus at ZT10, confirming the results of immunohistochemistry using dnBMAL1 mice (**Additional data 3** as shown below; data not shown in the manuscript). It is important to note that we performed western blotting using mice micro-infused AAV-dnBMAL1 into dorsal hippocampus since we could not prepare enough number of dnBMAL1 mice (although we are continuing to breed so that we can repeat this experiment in dnMBAL1 mice if necessary).

Furthermore, we examined the effects of knockdown of endogenous BMAL1 using micro-infusion of AAV expressing shBMAL1 into dorsal hippocampus. The results of western blotting showed that shBMAL1 reduced expressions of BMAL1, Per2 and Dbp at the protein levels in the hippocampus at ZT10 (**Additional data 4** as shown below**; Figure S4G**).

These results suggest that expression of dnBMAL1 efficiently blocks the function of endogenous BMAL1 function as knockdown of BMAL1 does it.

Additional data 2

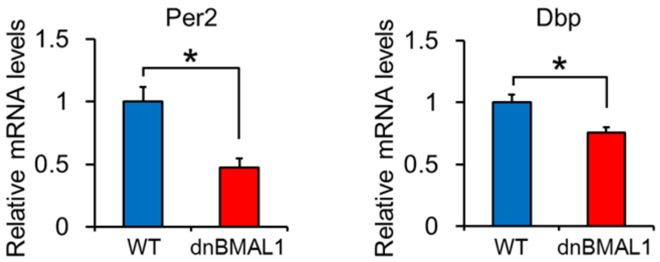

WT, n=4; dnBMAL1, n=4.
Mean ± SEM. Asterisk represents significant difference at $P < 0.05$.

Additional data 3

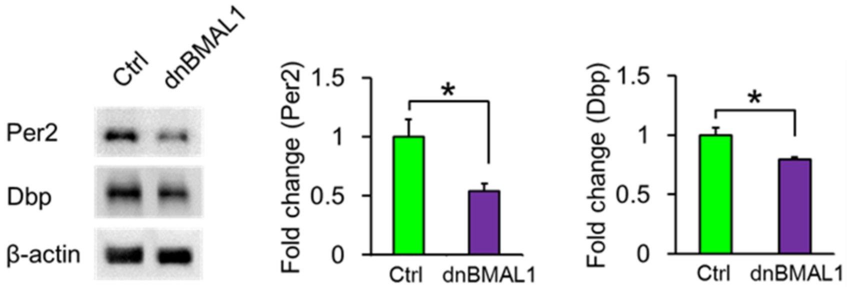

Ctrl, n=3; dnBMAL1, n=3.
Mean ± SEM. Asterisk represents significant difference at $P < 0.05$.

Additional data 4

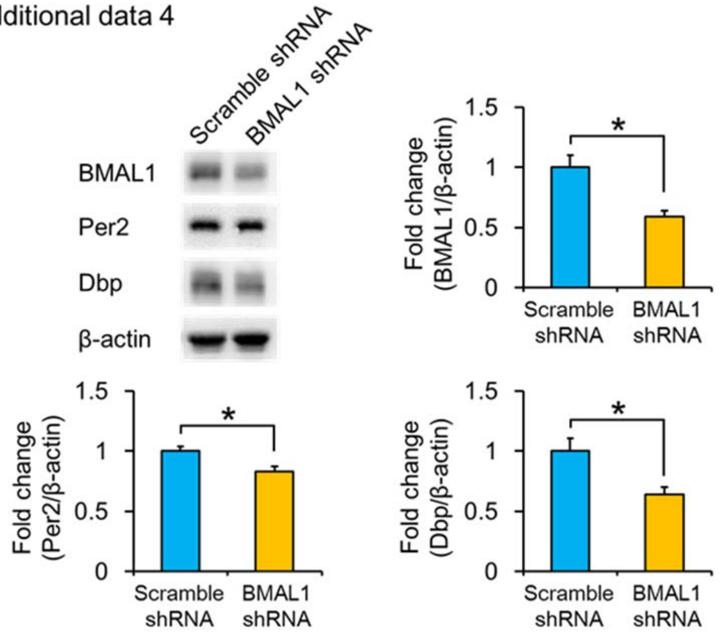

Scramble shRNA, n=3; BMAL1 shRNA, n=3.
Mean ± SEM. Asterisk represents significant difference at P < 0.05.

We added the **Additional data 2** and **4** into the revised supplemental figure 2I and 4G, respectively, and revised the supplemental figure legend as shown below (changed sentences are in red). We also present the primer sequences used to quantify the levels of Per2 and Dbp mRNA in Table S1.

Figure legends
(Figure S2I)
"(I) Quantification of Per2 and Dbp expression in the hippocampus (quantitative RT-PCR). Expressions of Per2 and Dbp are reduced in the hippocampus in dnBMAL1 mice at ZT10. The graph represents fold changes compared to expression levels in WT at ZT10. * $p < 0.05$."

(Figure S4G)
"(G) (Left panel) Representative images of BMAL1, Per2 and Dbp expressions in the dorsal hippocampus of control (scramble shRNA) and shBMAL1 (BMAL1 shRNA) groups 4-5 weeks after AAV injection (western blotting). (Right panels) Quantification of BMAL1, Per2 and Dbp expressions. One-way ANOVA with group reveals significant decreases of BMAL1, Per2 and Dbp expressions in the dorsal hippocampus of shBMAL1 group at ZT10 compared to control group. * $p < 0.05$. The graph represents fold changes compared to expression levels in control group."

Point 3)
(lines 124-125): There is not sufficient evidence to state that endogenous BMAL1 is reduced at ZT10. The message does appear to be lower (Figure 1C), however, in some peripheral tissues, Bmal1 message is actually anti-phase to the protein's transcriptional

activity and the peak of the chromatin occupancy. Since protein levels and chromatin recruitment aren't tested throughout the circadian cycle in this region, the conclusion that the deficit at ZT10 corresponds to "low Bmal1" is not appropriate. BMAL1 ChIP-seq should be performed to verify whether there is low BMAL1 recruitment to E boxes at this zeitgeber time.

Response) Thank you for your important comment. As suggested, we performed chromatin immunoprecipitation using anti-BMAL1 and anti-CLOCK antibody and examined binding to BMAL1/CLOCK to Dbp and Per2 promoters. Similar to Figure 2D, we observed blocking the BMAL1 and CLOCK binding to Dbp and Per2 promoter in the hippocampus of dnBMAL1 mice (**Additional data 5** as shown below; data not shown in the manuscript). More importantly, dnBMAL1 more efficiently blocked these bindings at ZT10 compared to ZT4. These results suggest that dnBMAL1 strongly inhibits the function of endogenous BMAL1 at ZT10, thereby leading to deficits of memory retrieval at ZT8-12.

However, it is important to note that WT mice showed more bindings of BMAL1 and CLOCK to Dbp and Per2 promoter at ZT10 compared to ZT4. Additionally, it is difficult to measure the time course activity of BMAL1 itself since BMAL1-mediated transcription is regulated not only by expression level of BMAL1 but also by abundant interacting factors with BMAL1 such as CLOCK, PERs, CRYs, TIMELESS and other transcription factors. Therefore, we decided to examine effects of BMAL1 knockdown using AAV expressing shBMAL1 (AAV-shBMAL1). We performed behavioral experiments using mice micro-infused AAV-shBMAL1 into dorsal hippocampus. Importantly, mice expressing shBMAL1 show similar retrieval deficits with dnBMAL1 mice; these BMAL1 knockdown mice show retrieval deficits in social recognition memory at ZT10, but not ZT4 (**Additional data 6** as shown below; **Figure S4H**). As shown above, AAV-shBMAL1 mice showed significantly reduced BMAL1, Dbp and PER2 protein levels in the hippocampus at ZT10, indicating that knockdown of BMAL1 expression reduced expression of BMAL1 and its target genes. These results suggest that the loss-of-BMAL1 function significantly impairs memory retrieval at ZT10 but not ZT4 and that more BMAL1 activities are required for memory retrieval at ZT10 compared to ZT4.

Taken together, these results suggest that loss-of-BMAL1 function showed greater impacts at ZT10 than at ZT4, supporting our conclusion that dnBMAL1 blocks endogenous BMAL1 activities more at ZT10 than at ZT4.

However, we do not still show direct evidence showing that ZT10 shows "low BMAL1". Therefore, we toned down our statements in the discussion. To understand mechanisms for impairments of memory retrieval by dnBMAL1, future studies are required to identify downstream genes of BMAL1 whose expressions directly reflect activities of BMAL1 and contribute to retrieval efficiency.

Additional data 5

Dbp promoter (E-box region)

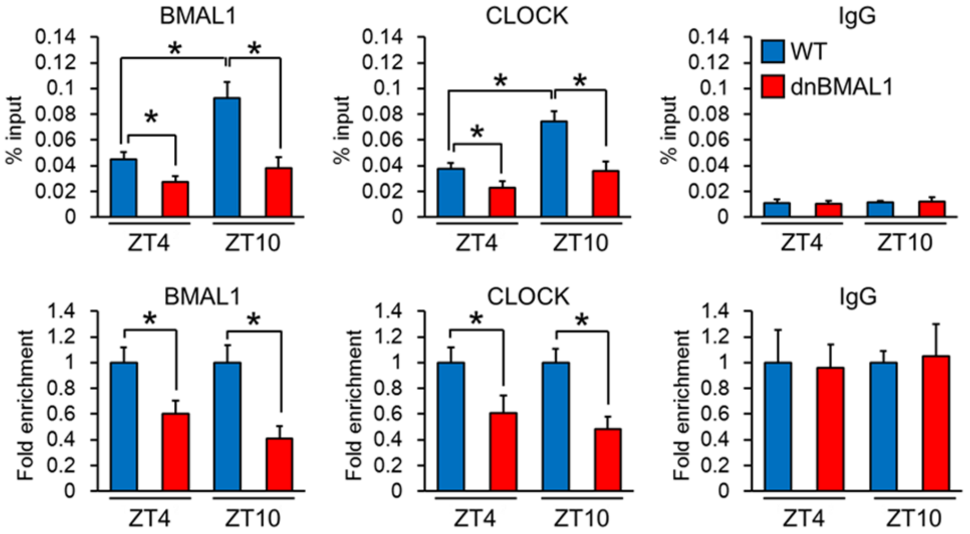

Per2 promoter (E-box region)

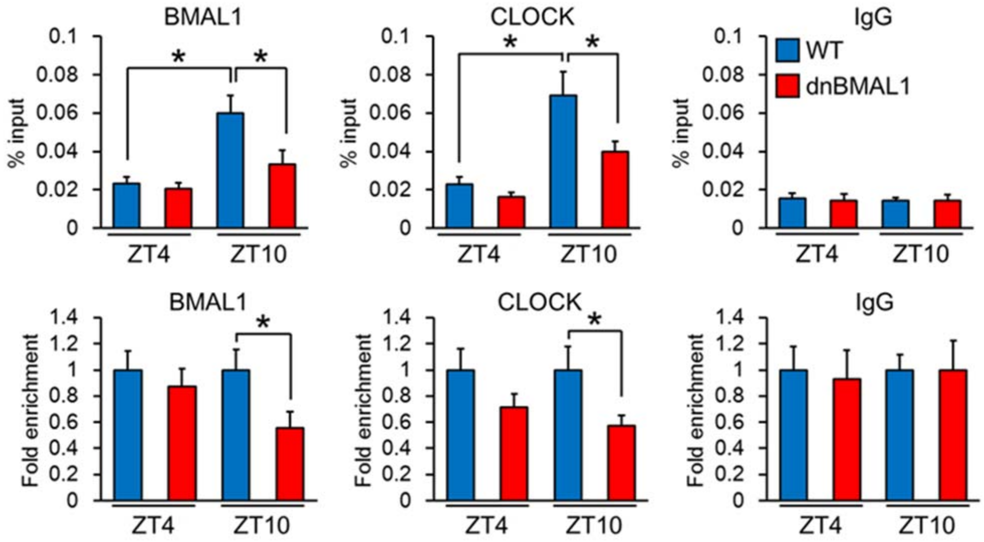

WT_ZT4, n=9; dnBMAL1_ZT4, n=9; WT_ZT10, n=9; dnBMAL1_ZT10, n=9.
Mean ± SEM. Asterisk represents significant difference at $P < 0.05$.

Additional data 6

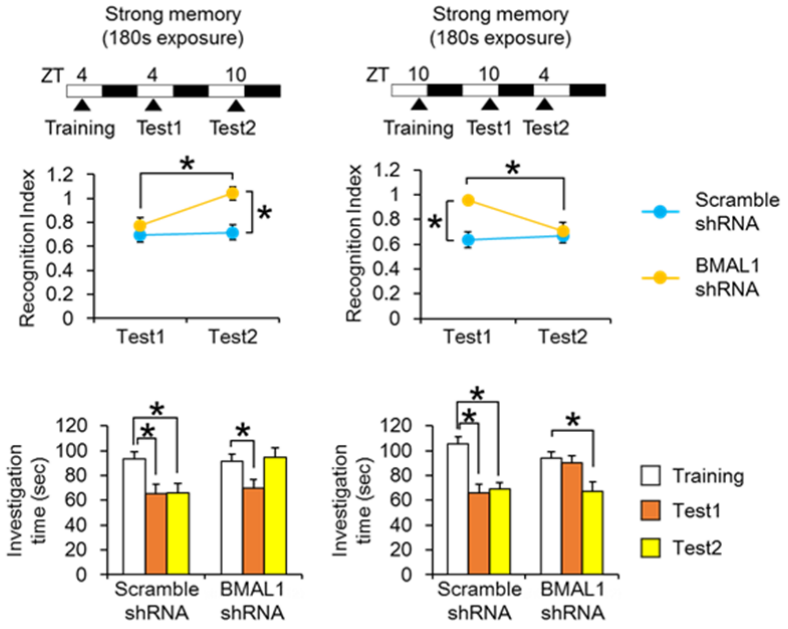

Scramble shRNA, n=13; BMAL1 shRNA, n=12.

Mean ± SEM. Asterisk represents significant difference at $P < 0.05$.

We added the **Additional data 6** into the revised supplemental figure 4H and revised supplemental figure legend as follows (changed sentences are in red).

Results

Expression of dnBMAL1 only in hippocampus disrupts memory retrieval

The retrieval impairments of hippocampus-dependent memories by forebrain expression of dnBMAL1 suggests that a hippocampal circadian clock regulates retrieval. To test this possibility, we examined effects of hippocampus-specific expression of dnBMAL1 using adeno-associated virus (AAV). We observed expression of EGFP co-expressed with dnBMAL1 in the dorsal hippocampus and found that dnBMAL1 expression reduced expression level of PER2 in the CA1 region of the hippocampus compared to control group (Figure S4D), suggesting that similar with dnBMAL1 mice (Figure 2B), viral expression of dnBMAL1 impairs hippocampal circadian clock. Importantly, mice expressing dnBMAL1, but not control group, showed impaired social recognition memory at ZT10, but not ZT4 (Figure 4D and Figure S4E). In contrast, dnBMAL1 group trained at ZT10 and tested at ZT4 showed normal recognition memory (Figure 4D and Figure S4E). It is important to note that dnBMAL1 group showed normal locomotor activity and anxiety-related behavior (Figure S4F). Importantly, we observed similar patterns of changes in retrieval performance by knockdown of BMAL1 expression in the hippocampus (Figure S4H). These observations indicate that hippocampal expression of dnBMAL is sufficient to impair memory retrieval at ZT10, suggesting that retrieval deficits observed in dnBMAL1 mice are due to impaired hippocampal clock.

Figure legends

(Figure S4H)

"(H) (Upper panels) shBMAL1 expression in the hippocampus impairs retrieval of social recognition memory at ZT10, but not ZT4 (Recognition index). (Lower left panel) Control and shBMAL1 mice significantly reduce investigation time in Test1 (ZT4, $p < 0.05$). shBMAL1, but not control, mice fail to reduce investigation time in Test2 (ZT10, $p > 0.05$). (Lower right panel) shBMAL1, but not control, mice fail to reduce investigation time in Test1 (ZT10, $p > 0.05$). Control and shBMAL1 mice significantly reduce investigation time in Test2 (ZT4, $p < 0.05$). Error bars represent SEM. * $p < 0.05$. "

We revised 1st paragraph of discussion as follows.

"Circadian rhythm of gene expression controlled by transcription factor BMAL1/CLOCK is observed in the SCN, thought to be the master circadian clock in mammals[1,2,4,5], and also in non-SCN regions [5-7]. Recent studies show that local peripheral circadian transcriptional clocks help regulate tissue-specific physiological and biological processes[1,8,9,14,21]. Although the results of several studies are consistent with the notion that circadian transcriptional clocks in the forebrain may also regulate circadian processes involved in memory[16-19,33-35], functional roles of local brain circadian clocks in memory performance remains unclear. Here, we show that fluctuations in forebrain (and, in particular, hippocampal) levels of the key clock transcription factor regulate retrieval efficiency. In WT mice, at times when, perhaps, BMAL1 activity is reduced, (e.g., ZT10) memory retrieval is impaired following weak (but not strong) training. Artificially blocking BMAL1 function (by expressing a dnBMAL1) in forebrain impaired memory retrieval at all times of day following weak training. These deficits were partially reversed when dnBMAL1 mice were trained with a strong protocol, but only at time points when, perhaps, BMAL1 activity was elevated (e.g., ZT4). Furthermore, a similar pattern of retrieval deficit (at ZT10) was observed when dnBMAL1 is expressed specifically in the hippocampus. Together, these findings suggest that efficiency of memory retrieval is regulated by a local circadian clock in the hippocampus.

Point 4)

While the restoration of retrieval by increasing cAMP signaling is convincing, the links between RNA-seq data and BMAL1 are very confusing. (This is in part due to the issue brought up in comment #3.) BMAL1 has dual roles- transcriptional and translational- and it is not clear based on the mouse model in which capacity the dominant negative BMAL1 is exerting its effects. cAMP has already been shown to be important for various aspects of memory processing (consolidation, retention, and retrieval), and thus the conceptual advance that this paper is missing is precisely how dnBMAL1 is affecting this pathway. This could be addressed by looking at BMAL1 in different subcellular fractions and at a minimum, by performing ChIP-seq or ChIP-qPCR experiments at different circadian times.

Response) Thank you for your important comment. We performed ChIP-qPCR and immunohistochemistry. We described these results of ChIP-qPCR at ZT10 as above (Response to point 3). These results showed that dnBMAL1 blocked binding of BMAL1 and CLOCK to Dbp and Per2 promoters (at ZT4 and ZT10), suggesting that dnBMAL1 functions at the transcriptional level (see above). Additionally, to examine the localization of BMAL1 in the neuron, we performed immunohistochemistry using anti-BMAL1 antibody and found that BMAL1 is localized in the nucleus in the CA1 region of the hippocampus of both WT and dnBMAL1 mice at ZT4 and ZT10 (**Additional data 7** as shown below; data not shown in the manuscript). This result suggests that BMAL1 is mainly localized in the nucleus, but not in the cytosol and other compartments and regulates transcription of target genes. Taken together, these

observations suggest that dnBMAL1 show dominant negative effects in the nucleus at the transcription level.

As reviewer suggested, we need to understand mechanisms for expression regulation of cAMP-related genes by BMAL1. This is a significant undertaking, and we will pursue these studies in the future. However, we feel that they fall beyond the scope of the current paper.

Additional data 7

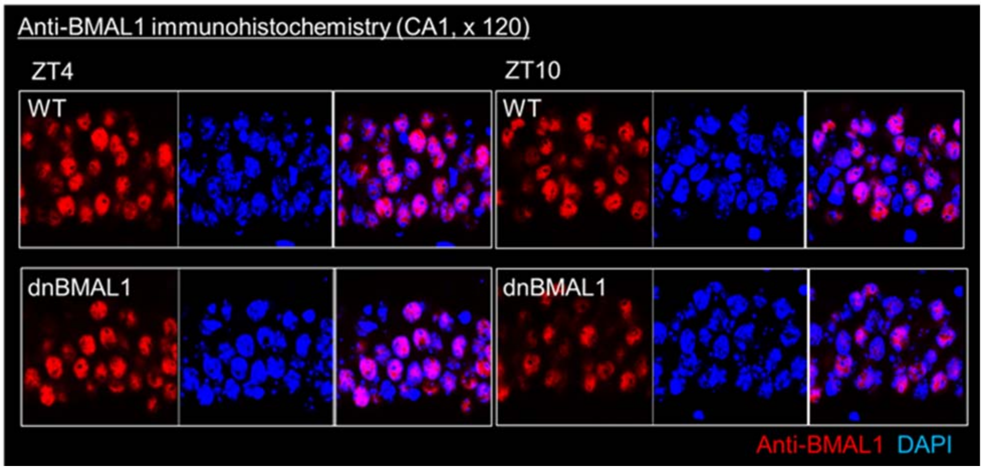

Reviewer #2 (Remarks to the Author):

Various aspects of cognitition including memory consolidation depend on the circadian cycle. For example, memory retrieval declines in the late afternoon-early evening and maintenance of long-term memory depends on the circadian cycle. However, the role of local brain circadian clocks in memory remains unclear. In this paper they convincingly demonstrate that the hippocampal clock regulated by BMAL1 regulates time-of-day retrieval. Inducible transgenic dominant negative BMAL1 (dnBMAL1) expression in the mouse forebrain or hippocampus compromised retrieval of hippocampal memories at Zeitgeber Time 8-12, independent of retention delay, encoding time or Zeitgeber entrainment cue. This altered retrieval profile was apparently due to down regulation of hippocampus dopamine-cAMP signaling in dnBMAL1 mice. This decreased dopamine receptors and GluA1-S845 phosphorylation by PKA. Pharmacological activation of cAMP-signals or D1/5Rs rescued impaired retrieval efficiency in dnBMAL1 mice. GluA1 S845A knock-in mice showed retrieval deficits with dnBMAL1 mice. These data suggest mechanisms underlying regulation of retrieval efficiency by hippocampal clock through the D1/5R-cAMP-PKA- GluA1 pathway.

This an interesting and novel study suitable for publication in Nature Communications. It provides a new molecular mechanism that explains time-of-time dependence for memory retrieval. The major conclusions are justified by the data, the paper is clearly written and the data is technically solid. I recommend that the paper be accepted for publication.

Reviewer #3 (Remarks to the Author):

This manuscript describes clock mechanisms regulating circadian effects on memory retrieval. The authors claim mechanisms underlying time of day effects on cognitive performance and memory are unknown (e.g., see in introduction, line 60: "Although it has been known since experiments by Ebbinghuas (1885) that time-of-day influences cognitive performance and memory…the mechanisms underlying this effect are unknown"). However, circadian and time-of-day effects of learning and memory are known to exist across animal phyla, and there is extensive evidence for core-clock machinery in regulating this process, including the brain regions they describe; their claims should better reflect the current state of the field (see below). Here in this report, the authors focus on the molecular pathways involving the transcription factor BMAL1 and Dopamine-cAMP pathways regulating hippocampus-dependent memory. The authors primarily use a hippocampus-dependent social recognition task where adult mice are exposed to juvenile mice, and the amount of exploration time of the adult mouse during a follow-up test trial with the same juvenile is indexed as memory. Varying the amount of initial exposure time to the juvenile generates a corresponding variance in recognition memory. The authors first describe time-of-day effects of weaker (2min) training that exists when trained and tested at ZT4 (24hr memory) but is blocked when trained and tested at ZT10. They then trained at ZT4 and tested at ZT10 and show impairment, which was not observed when animals were trained at ZT10 and tested at ZT4. The authors have adequate controls showing that stronger training (3min) abolishes this time-of-day effect. The authors then show diurnal changes in hippocampal BMAL1 RNA- although the methods are unclear (see below).

The authors then describe 2 spatial- and temporally-controlled dominant-negative (dn) BMAL mouse models, where they can induce dnBMAL1 in forebrain regions using a CAMKii promoter and the tet (tTA) on-off system. In this model, the removal of Dox treatment induces the dnBMAL1 in hippocampus (and other forebrain regions). These mice show reduced expression of downstream BMAL1 target genes, and normal expression in the SCN region and normal circadian locomotor activity (good controls for brain target specificity). The authors then use this model to test whether time-of-day dependent social-recognition retrieval is affected by dnBMAL in forebrain regions and show deficits at ZT4. Line 131 reads "deficits at both ZT4 and ZT10…" however, figures 3A and S3A show a dnBMAL deficit only at ZT4, with no differences between WT and dnBMAL1 at ZT10; therefore, this statement needs to be corrected (see below). The authors then use a strong training protocol to test dnBMAL mice, and survey many points over the day to show 24hr retrieval differences only at ZT8, ZT10, and ZT12 compared to WT. The authors then report differences in retrieval with training at ZT4 and testing at ZT10 with the strong training protocol. They did not observe differences in these protocols under DD (constant dark) conditions, suggesting they are independent of external light cues. The authors provide supplemental data that describes the dnBMAL effects on social retrieval only when the transgene is turned ON (Figure S3) but the results, legend, and methods all fail to describe adequately the timing of Dox treatment (ON/OFF vs ON/OFF/ON, etc.) which needs to be addressed (see below).

The authors also test dnBMAL mice in object recognition and contextual fear conditioning and show the time-of-day dependent effects of dnBMAL are generalizable to different forms of memory. The data presented are confusing to what is described in the results (see below). The authors then performed RNA-seq on dnBMAL1 mice and report the identification reductions in RNA for adenylate cyclase and dopamine receptors (D1R and D5R), along with reductions in cAMP levels, compared to WT mice. They go on to show blocking hippocampal cAMP degradation (PDE4) activity with microinfusion of rolipram rescues the dnBMAL effects following strong training at ZT10, as well as retrieval following weak training in WT mice at ZT10. IP injection of a D1/5R agonist rescued dnBMAL1 retrieval deficits at ZT10, with no effects on WT, but another D1/5R agonist impaired WT retrieval at ZT10 but not ZT4. This last observation is challenging

to understand and deserves attention, either experimentally, or minimally in the discussion (see below).

Lastly, the authors rationalize that D1/5R activation phosphorylates AMPA receptor GluA1 via PKA, so they examined activity of the phosphorylation site on GluA1 (S845) in the synaptosomal fractions of hippocampus at ZT10 in dnBMAL mice compared to WT mice, and found a reduction in dnBMAL mice. The authors then used GluA1 S845A knock-in mice to test the effects on time-of-day dependent social recognition and contextual fear conditioning. They observed impairments at ZT10 testing but not ZT4 in S845A knock-in mice compared to WT; mimicking the effects of dnBMAL mice.

Minor concerns:

Point 1)

The authors make the claim that the role of mechanisms in time-of-day memory/cognitive performance is unknown. This is not an accurate reflection of the current state of the field, and several statements of the manuscript should be rephrased to better represent this. For example, in the introduction, in line 63 should be edited from "…this effect are unknown" to read "…this effect are not well understood " – similarly, in line 258 of the discussion, the authors state that "…no study has examined this directly" while citing Shimizu et al., 2016 (Nature Comm.) in reference to circadian transcriptional clocks in the forebrain regulating circadian memory processes. Shimizu et al., (Nature Comm. 2016) tested the effects of BMAL conditional knock-out mice on circadian gene expression and long-term NOR memory. These mice have BMAL expression preserved in the SCN, but not in the hippocampus and other brain regions, which is important since this determined whether circadian expression of core clock genes in the SCN or forebrain/hippocampal circuits conferred the circadian effects on long-term NOR memory. Shimizu et al. showed that BMAL cKO expression blocks circadian regulation of genes in the hippocampus, and blocks circadian changes in NOR memory, which already described circadian regulation of clock genes in the hippocampus are required for circadian regulation of NOR long-term memory. Shimizu et al. also show it is the time of training, and not the time of testing, that appears to affect this temporal difference in long-term memory formation. This contrasts with what the current authors observed here in the case of their dnBMAL1 model, where instead it is the time of retrieval. The authors should dedicate space within the discussion to address this discrepancy with previous studies.

Response) Thank you for your important, critical and helpful suggestions. We revised introduction and discussion sessions as follows (changed sentences are in red).

Introduction 2nd paragraph

Interestingly, in addition to the SCN, BMAL1/CLOCK-mediated transcriptional rhythms are observed in other parts of the brain, as well as peripheral tissues including, muscle and liver[5-7]. The SCN generates circadian behavioral rhythms and synchronizes the local clocks in peripheral tissues[1,2]. Importantly, there is increasing evidence that molecular clocks in these extra-SCN regions exert autonomous circadian regulation of tissue-specific functions[1,8-14]. Although it has been known since experiments by Ebbinghaus (1885) that time-of-day influences cognitive performance and memory (wherein performance declines in the late morning to early evening[15]), the mechanisms underlying this effect are not well understood[16-19]. Here, we investigated the local role of this molecular clock in the hippocampus in memory processes using mice.

Discussion 1st -3rd paragraph

Circadian rhythm of gene expression controlled by transcription factor BMAL1/CLOCK is observed in the SCN, thought to be the master circadian clock in mammals[1,2,4,5], and also in non-SCN regions[5-7]. Recent studies show that local peripheral circadian transcriptional clocks help regulate tissue-specific physiological and biological

processes[1,8,9,14,21]. Although the results of several studies are consistent with the notion that circadian transcriptional clocks in the forebrain may also regulate circadian processes involved in memory[16-19,33-35], functional roles of local brain circadian clocks in memory performance remains unclear. Here, we show that fluctuations in forebrain (and, in particular, hippocampal) levels of the key clock transcription factor regulate retrieval efficiency. In WT mice, at times when BMAL1 levels are reduced (e.g., ZT10) memory retrieval is impaired following weak (but not strong) training. Artificially reducing BMAL1 levels (by expressing a dnBMAL1) in forebrain impaired memory retrieval at all times of day following weak training. These deficits were partially reversed when dnBMAL1 mice were trained with a strong protocol, but only at time points when endogenous levels of BMAL1 were elevated (e.g., ZT4). Furthermore, a similar pattern of retrieval deficit (at ZT10) was observed when dnBMAL1 is expressed specifically in the hippocampus. Together, these findings suggest that efficiency of memory retrieval is regulated by a local circadian clock in the hippocampus.

In humans, time-of-day can influence the efficiency of cognitive processing, including memory retrieval[1]. While this effect has been recognized for over a century, the underlying neurobiological mechanisms are not understood. Strikingly, our findings suggest that the rates of BMAL1-mediated transcription determine retrieval success (Figure 7). When BMAL1 activity is upregulated (e.g., at ZT4), retrieval is enhanced whereas when BMAL1 activity are downregulated (e.g., ZT10) retrieval is impaired. These data identify a role for BMAL1 in memory processing, and indicate that time-of-day effects on memory retrieval are mediated by local, hippocampal cell-autonomous oscillators.

Importantly, a previous study showed that WT mice could form long-term object recognition memory when trained only at ZT8-16 (but no long-term memory when trained at other zeitgeber times), whereas BMAL1 conditional KO (cKO) mice showed deficits of this type of memory at ZT4 and ZT16[34]. In contrast, our results indicated that dnBMAL1 mice showed retrieval deficit of object recognition memory at ZT10, but not ZT4, in experimental conditions where WT mice show normal recognition memory at both time points. In order to reconcile these findings, future work should directly compare WT, dnBMAL1 and BMAL1 cKO mice using stronger and weaker training conditions. Such experiments will help to clarify time-of-day regulation of memory performance (memory formation VS retrieval) by local clocks (brain regions-specific roles).

Point 2)
The authors need to better describe the differences in hippocampal changes in BMAL expression (Fig. 1C)- which technique was used, qPCR or FISH? While the methods describe both techniques, it is not clear which is used in Figure 1C, as it is not mentioned in the results, legend, or RNA analysis section of the methods. How was the hippocampus isolated for RNA work? What region(s) – CA1, CA3, Dentate gyrus, or all?

Response) Thank you for your helpful suggestion. We revised in Figure legend as follows (changed sentences are in red). We also showed the primer sequences to quantify the level of BMAL1 mRNA in Table S1.

Figure legends
Figure 1. Retrieval of social recognition memory is impaired at ZT10 following weak training in wild–type (WT) mice
(C) BMAL1 mRNA levels in the whole hippocampus are reduced at ZT10 compared to other time points (quantitative RT-PCR). The graph represents fold changes compared to expression levels at ZT4. * $p < 0.05$, compared to the other time points. Error bars represent SEM. * $p < 0.05$.

Point 3)
The authors need to describe the conditions (timing/etc.) for what dnBMAL ON/OFF and ON/OFF/ON mean in terms of training/testing in social recognition task (Fig S3). This should be clearly laid out minimally in methods.

Response) Thank you for your helpful suggestion. We revised Method section as follows (changed sentences are in red).

Administration of doxycycline (Dox)
TRE promoter-dependent transgene expression was regulated using the animal's (dnBMAL1 and WT mice) drinking water containing 100 µg/mL doxycycline (Dox, Sigma-Aldrich, St Louis, MO, USA) dissolved in 5% sucrose to mask the bitter taste of Dox. To minimize potential effects of dnBMAL1 expression during development, mice were treated with Dox until 8 weeks of age (transgene "OFF") at which time Dox was removed (transgene "ON") to induce dnBMAL1 expression (dnBMAL1 OFF/ON mice). dnBMAL1 ON/OFF mice were not treated with Dox until 8 weeks of age (transgene "ON") at which time Dox treatment was started (transgene OFF). dnBMAL1 ON/OFF/ON mice were treated with Dox from 4 to 8 weeks of age (see Figure S2B).

Point 4)
In Figure4A (right panel), the X-axis looks to be reversed for Testing at ZT10 versus ZT4; the current X-axis shows ZT4 has differences and ZT10 does not- but the results section explains ZT10 has the differences (lines 170-173).

Response) Thank you for your helpful suggestion. We corrected the graph in Figure 4A as follows.

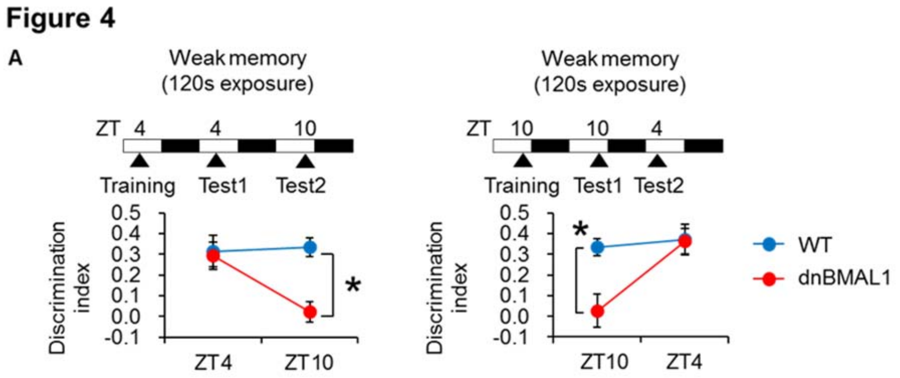

Point 5)
Figure 5G shows the effects of D1/5R agonist SKF38393 does not influence strong social recognition test in WT mice at ZT10, however, Figure 5H shows the effects of D1/5R agonist SKF83566 on social recognition retrieval following strong training in WT mice at ZT10. At ZT4, there does not appear to be any effect of SKF83566, however, there is a significant deficit at ZT10 in WT mice. In the discussion, line 282-285 reads "Importantly, pharmacologically restoring cAMP levels or D1/5R activities rescued retrieval deficits of strong and weak memory observed in dnBMAL1 mice and WT mice, respectively, at

ZT10." This statement is not supported by these data. There are multiple issues. Please parse out these observations and handle them in the discussion separately. For example, rephrase the statement in line 282-285 to something like "Importantly, pharmacologically restoring cAMP levels or D1/5R activities rescued retrieval deficits of strong memory observed in dnBMAL1 mice at ZT10" and then return to the issues with SKFs and WT mice: "However, D1/5R activity in WT mice was able to impair strong recognition at ZT10 with SKF83566, but not with SKF38393. " <then explain why you think this is>. Also, why was this second agonist performed in WT and not dnBMAL mice? The authors may choose perform additional experiments on SKF83566 on dnBMAL mice with strong training to complement the WT studies, or omit the WT studies with SKF83566 all together. The better experiment testing D1/5R agonist (SKF38393) was already performed with dnBMAL and WT at ZT10 following strong training (Figure 5G).

Response) It is important to note that "SKF83566" is an "antagonist" (not agonist) for D1/5R and that therefore, as might be expected, a SKF83566 impairs retrieval of (strong) memory only at ZT10, but not ZT4, in WT mice (Figure 5H). This result indicated that blocking D1/5R activity by low dose of antagonist is sufficient for impairments in retrieval at ZT10 in WT mice, suggesting that WT mice show impaired retrieval at ZT10 compared to ZT4.

In addition, we showed that pharmacologically restoring cAMP levels rescued retrieval deficits of weak memory in WT mice at ZT10 (Figure 5F). Taken together of this observation with results of dnBMAL1 mice, we stated that "pharmacologically restoring cAMP levels or D1/5R activities rescued retrieval deficits of strong and weak memory observed in dnBMAL1 mice and WT mice, respectively, at ZT10." However, this sentence may have been confusing.

Therefore, to make sure, we revised this sentence as follows (changed sentences are in red).

"pharmacologically restoring cAMP levels or D1/5R activities rescued retrieval deficits of strong memory observed in dnBMAL1 mice at ZT10 (following strong training), whereas similarly restoring cAMP levels rescued deficits in weak memory retrieval in WT mice at ZT10 (following weak training)."

Results

Activation of Dopamine-cAMP signaling rescues retrieval deficits in dnBMAL 1 mice

Taken together with previous findings that suggested the important roles of cAMP signaling pathways for memory retrieval[29], we hypothesized that the decrease in cAMP production mediated by Dopamine-cAMP-mediated signal transduction impairs retrieval efficiency (Figure 5D). Therefore, we first asked whether increasing cAMP levels or activation of D1/5R would rescue the retrieval deficits at ZT10 in dnBMAL1 mice. WT and dnBMAL1 mice were trained at ZT10 and tested twice every 24 h (Test 1 and 2) after the strong social recognition training. Prior to Test 1, mice received a micro-infusion of rolipram[30] (a PDE4 inhibitor) into dorsal hippocampus (Figure 5E and S5A). This micro-infusion did not affect memory retrieval at Test 1 and 2 in WT mice. However, dnBMAL1 mice treated with rolipram showed normal memory retrieval in the presence (Test1), but not absence (Test2), of rolipram infusion although these mutant mice treated with vehicle showed retrieval impairments at Test 1 and 2. (Figure 5E and S5A). These observations indicate that rolipram rescued the retrieval deficit at ZT10 in dnBMAL1 mice. Furthermore, this micro-infusion of rolipram into dorsal hippocampus, but not mPFC, of WT mice rescued the retrieval deficit of weak social recognition memory at ZT10 (Figure 5F, Figure S5B, S5C and S5D). A similar pattern of retrieval rescue at ZT10 was observed when dnBMAL1 mice were systemically injected with rolipram (Figure 5G and S5E). Importantly, pharmacological activation of Dopamine Receptor D1/5R by systemic injection of D1/5R agonist (SKF38393) rescued the retrieval deficit in

dnBMAL1 mice (Figure 5G and S5E). Conversely, the inactivation of D1/5 receptors by D1/5R antagonist (SKF83566) impaired the retrieval in WT mice at ZT10, but not ZT4 (Figure 5H and S5F), supporting our conclusion that WT mice show lower retrieval efficiency at ZT10 compared to ZT4. Taken together, these observations suggest that retrieval deficits observed in dnBMAL1 (strong memory) and WT mice (weak memory) are mediated by impaired Dopamine D1/5R-cAMP signal transduction.

Discussion 4th paragraph

The molecular mechanisms by which memory retrieval is regulated have remained unclear. Our studies indicate that BMAL1 modulates retrieval efficiency via regulation of Dopamine/cAMP signaling. Our RNA-seq analyses identified downregulation of cAMP-mediated signaling related gene expressions in the hippocampus of dnBMAL1 mice. Consistently, dnBMAL1 mice show reduced AC1 mRNA, target of BMAL1/NPAS2 in the retina[36], and cAMP levels in the hippocampus of dnBMAL1 mice at ZT10 when memory retrieval efficiency dips in these mutant mice (Figure 3). Moreover, mRNA levels of D1R and D5R, activators of cAMP signals, are decreased in the hippocampus of dnBMAL1 mice. Importantly, pharmacologically restoring cAMP levels or D1/5R activities rescued retrieval deficits of strong memory observed in dnBMAL1 mice at ZT10 (following strong training), whereas similarly restoring cAMP levels rescued deficits in weak memory retrieval in WT mice at ZT10 (following weak training). Therefore, our findings suggest that hippocampal BMAL1 upregulates retrieval efficiency by activating Dopamine-D1/5R-cAMP signaling in the hippocampus. These findings are consistent with previous observations that cAMP levels show circadian rhythms in hippocampus[16] and that cAMP-signaling pathway modulates memory retrieval[29].

Point 6)

It is unclear why the authors use the term "efficiency" to describe the circadian effects retrieval memory. What exactly does this mean? How is the audience to interpret this term? The term is never defined, remains ambiguous, and should be removed from the manuscript. It detracts from the substantial enthusiasm of this report. Discarding this "efficiency" term from the manuscript will avoid confusion.

Response) Thank you for your helpful suggestion. We agree with this comment. We revised the manuscript; all of "retrieval efficiency" were revised (changed sentences are in red).

Point 7)

It would greatly help the reader if the authors label in the Figures "Strong" or "Weak" where applicable.

Response) Thank you for your helpful suggestion. We added "Strong memory" or "Weak memory" in each Figure.

Reviewers' Comments:

Reviewer #1:
Remarks to the Author:
In the revision of “Hippocampal clock regulates memory retrieval via Dopamine and PKA-induced GluA1Phosphorylation” Hasega et al. address several of the concerns of mine related to the relationship between BMAL1 activity at chromatin vs. expression, its relationship to cAMP signaling and oscillations, as well as a number of other experiments critical to interpretation of the data. The authors did a substantial amount of new work (thought the choice of what to put in the paper was in places questionable to me). However, I admit that the authors did a substantial amount of work and it may be difficult to put all of the data in the manuscript.

I have one remaining concern, and that relates to the lack of PER2 and Dbp expression data in the dnBMAL1. The authors state that they do not have sufficient animals to perform the western, but it seems that this piece of data is key to the manuscript since so much behavior is performed in dnBMAL1 mice. Since they show the optical density data, they must have the westerns for the quantification. I think that these westerns still need to be presented in the manuscript and not just the optical density from the quantification.

Other than this point, I appreciate the great pains the authors have gone to in order to satisfy my original critique. The manuscript is greatly improved.

Reviewer #2:
Remarks to the Author:
The authors of this paper have extensively revised this manuscript and provided a large amount of additional data.The paper has been appropriately revised and is now suitable for publication in NNS.

Reviewer #3:
Remarks to the Author:
The authors have adequately addressed concerns.
JRGerstner

Reviewer #1 (Remarks to the Author):

In the revision of “Hippocampal clock regulates memory retrieval via Dopamine and PKA-induced GluA1Phosphorylation” Hasega et al. address several of the concerns of mine related to the relationship between BMAL1 activity at chromatin vs. expression, its relationship to cAMP signaling and oscillations, as well as a number of other experiments critical to interpretation of the data. The authors did a substantial amount of new work (thought the choice of what to put in the paper was in places questionable to me). However, I admit that the authors did a substantial amount of work and it may be difficult to put all of the data in the manuscript.

I have one remaining concern, and that relates to the lack of PER2 and Dbp expression data in the dnBMAL1. The authors state that they do not have sufficient animals to perform the western, but it seems that this piece of data is key to the manuscript since so much behavior is performed in dnBMAL1 mice. Since they show the optical density data, they must have the westerns for the quantification. I think that these westerns still need to be presented in the manuscript and not just the optical density from the quantification.

Other than this point, I appreciate the great pains the authors have gone to in order to satisfy my original critique. The manuscript is greatly improved.

Response) Thank you for your important comment. We performed western blotting and found that dnBMAL1 mice showed significantly reduced Per2 and Dbp protein levels in the hippocampus at ZT10, confirming the results of immunohistochemistry (Figure 2B) and quantitative RT-PCR (Figure S2I) using dnBMAL1 mice (**Additional data 1** as shown below; **Figure S2J**).

Additional data 1

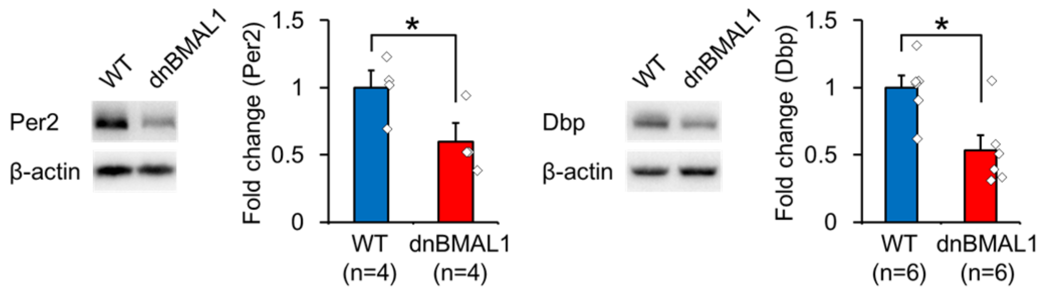

Mean ± SEM. Asterisk represents significant difference at P < 0.05.

We added the **Additional data 1** into the revised supplemental figure 2J and revised the supplemental figure legend as shown below (changed sentences are in red).

Figure legend

(Figure S2J)

“(J) (Left panel) Representative images of Per2 and Dbp expressions in the hippocampus of WT and dnBMAL1 mice (western blotting). (Right panels) Quantification of Per2 and Dbp expressions. One-way ANOVA with group reveals

significant decreases of Per2 and Dbp expressions in the hippocampus of dnBMAL1 mice at ZT10 compared to WT mice. The graph represents fold changes compared to expression levels in WT at ZT10. * $p < 0.05$.”

Reviewer #2 (Remarks to the Author):

The authors of this paper have extensively revised this manuscript and provided a large amount of additional data. The paper has been appropriately revised and is now suitable for publication in NNS.

Reviewer #3 (Remarks to the Author):

The authors have adequately addressed concerns.
